# Smiling across borders: Host culture members' reactions to happiness expressed by immigrants

**DOI:** 10.1111/bjso.70030

**Published:** 2026-01-07

**Authors:** Magdalena Bobowik, José J. Pizarro, Patrycja Slawuta, Nekane Basabe

**Affiliations:** ^1^ Department of Social Psychology University of the Basque Country Donostia/San Sebastián Spain; ^2^ IKERBASQUE Basque Foundation of Science Bilbao Spain; ^3^ Universidad Católica del Norte Antofagasta Chile; ^4^ New School of Social Research New York New York USA

**Keywords:** disadvantaged groups, emotions, happiness display, helping, immigrants, smiling, social interaction intentions, stereotype content model

## Abstract

Smiling is widely recognized for facilitating interpersonal relationships, yet its effects in intergroup, non‐cooperative contexts remain underexplored. Across four experiments and six samples (*N*_total_ = 2074) in Spain and the US, we explored how displays of happiness by immigrants from various ethnocultural groups (vs. non‐immigrants) influence host culture members' emotions, perceptions, social interaction and avoidance intentions and helping intentions. Results showed that representations of smiling immigrants were associated with higher perceptions of warmth and competence, eliciting positive emotions like joy, admiration and feeling moved, while reducing negative emotions like anger. Host culture perceivers were more willing to engage with, and less likely to avoid, smiling immigrants, largely due to increased perceptions of warmth and likability. However, these representations did not significantly increase helping intentions. The role of group membership of the expresser in moderating these effects was limited. The findings suggest that immigrants' displays of positive emotions in awareness‐raising social campaigns and mass media could serve as a counter‐strategy to prevalent negative stereotypes. However, our research also indicates that host societies may condition the acceptance and naturalization of immigrants on the emotions they express, their perceived ‘successful’ adaptation or demonstrated agency.

## INTRODUCTION

Facial expressions profoundly influence our social interactions and relationships. In diverse social environments, group membership dynamics affect emotional communication. Studies reveal challenges in recognizing and mimicking outgroup members' emotional expressions (e.g. Hess et al., 2022). In parallel, interactions with outgroups typically have lower frequency, quality and positivity compared to ingroups (Koudenburg, 2018), also reflected in cooperative behaviours (Balliet et al., 2014). Emotional cues in these interactions can either diminish or intensify such intergroup biases.

Smiling, as a specific emotional cue, may significantly shape social outcomes in intercultural contexts. There is a notable bias where positive expressions like happiness are more readily attributed to, or recognized in, ingroup than outgroup faces (Beaupré & Hess, 2003; Bijlstra et al., 2010; Martin et al., 2024). Further, while smiles might signal affiliation or dominance (Martin et al., 2017; Rychlowska et al., 2017), genuine smiles typically occur in cooperative interactions among equals, where smiles encourage further interaction (Bernstein et al., 2010; Mussel et al., 2013; Scharlemann et al., 2001) and promote helping (Guéguen & De Gail, 2003; Vrugt & Vet, 2009). In diverse cultural environments, conversely, changes in emotional patterns can present both challenges and opportunities for emotional expression and recognition (De Leersnyder, 2017) –even for more universally recognizable emotions like smiling (Niedenthal et al., 2018). Research has also shown that context can affect how smiles are evaluated (Day et al., 2023; Olszanowski & Tołopiło, 2024) but understanding how smiles are perceived and responded to in complex, asymmetric social relationships remains limited.

Clarifying the role of emotion expression in an asymmetrical intergroup context, particularly at the crossing of immigrants and host cultures, has relevant practical implications. Mass media often depict immigrants negatively, highlighting negative emotions like anger and portraying them in large groups without visible facial features (e.g. Bleiker et al., 2013), which results in their dehumanization (Azevedo et al., 2021). There is also a general propensity to attribute negative emotions, such as anger or sadness, more to outgroup than ingroup faces (e.g. Hugenberg & Bodenhausen, 2003), including in ethnocultural groups stereotypically representing immigrants (e.g. Bijlstra et al., 2010, 2014). Counter‐stereotypical and personalized representations, where immigrants show positive emotions with distinct facial features, could help reduce discrimination and prejudice. Enhancing positive representations of immigrants can promote their inclusion and help address systemic obstacles they face in critical domains such as housing, employment, education and health services.

We acknowledge, however, that positively framed representations of immigrants can be a double‐edged sword. Such portrayals may prescribe particular ways of emotionally assimilating into the host culture, effectively producing a ‘smile or die’ scenario. This perpetuates the neoliberal notion that optimal functioning and happiness result from individual effort and personal life choices (van Zyl et al., 2024). Furthermore, positive contact with the socially advantaged groups in society can demobilize the disadvantaged (Hässler et al., 2020). Consequently, smiling representations might lead the host culture members, and the immigrants themselves, to view them as submissive and disempowered. These portrayals risk reinforcing the status quo and perpetuating unequal relationships, rather than fostering genuine inclusion and empowerment.

In four experiments across five distinct samples, we investigated how representations of immigrants expressing happiness affect intergroup dynamics with host culture members. Guided by the Emotions as Social Information (EASI) Model (van Kleef, 2009), which proposes that emotional expressions shape perceivers' inferences, emotional reactions and behaviours, and the Behaviours from Intergroup Affect and Stereotypes (BIAS) Map Model (Cuddy et al., 2007), which suggests that stereotypes and intergroup emotions predict specific behavioural tendencies, we examined whether immigrants' expressions of happiness could promote host culture members' positive cognitive, emotional and behavioural responses—including greater willingness to interact, reduced avoidance and increased help provision.

Additionally, informed by the Meaning of Emotion Expressions in Context (MEEC) Model (Hess & Hareli, 2017), which highlights the importance of group membership and power asymmetries in interpreting emotional expressions, we focused on asymmetrical intergroup contexts by analysing host culture members' reactions to immigrants' smiles. Specifically, in Studies 3 and 4, we directly compared responses to smiles expressed by immigrants versus host culture members across different ethnocultural groups.

Finally, in line with the EASI and BIAS Map models, we explored psychological mechanisms underlying host culture members' affiliative intentions towards immigrants. We tested the mediating roles of perceived warmth and competence, as well as emotional responses (joy, liking, admiration, *kama muta*—being moved by love—and anger).

### Smiling and cognitive inferences

Facial expressions play a pivotal role in social communication, shaping perceivers' cognitive appraisals, emotional reactions and behavioural responses (e.g. van Kleef, 2009; van Kleef & Côté, 2022). A simple smile can substantially influence how individuals are perceived, often enhancing judgements of warmth, competence and trustworthiness. Given that immigrants and other outgroup members are typically perceived as less warm and competent than ingroup members (Fiske et al., 2002; Lee & Fiske, 2006), it is crucial to examine whether smiling can mitigate such biases.

Perceptions of warmth—involving traits such as friendliness and kindness—and competence—linked to intelligence and ability—are central to how individuals evaluate members of different ethnocultural groups (Fiske et al., 2002). Smiling generally promotes more favourable impressions across both dimensions. It is commonly interpreted as a signal of cooperative intent, social openness and acceptance (Harker & Keltner, 2001; Heerdink et al., 2015; Reed et al., 2012; Scharlemann et al., 2001), even in contexts that are not overtly cooperative (Rychlowska et al., 2021).

Extensive research shows that individuals who smile are rated as more sincere, moral, sociable, approachable and friendly (Belkin & Rothman, 2017; Hess et al., 2002; Matsumoto & Kudoh, 1993; Senft et al., 2016). Smiles may also enhance perceptions of competence and confidence (Belkin & Rothman, 2017; Hess et al., 2002) and, in some cultural contexts, even intelligence (Krys et al., 2016). Moreover, recent studies confirm that immigrants' visual and emotional expressions influence cognitive inferences drawn by observers (Azevedo et al., 2021; Bobowik et al., 2023).

### Smiling and felt emotions

Emotional expressions—especially smiles—elicit affective responses in observers and shape interpersonal dynamics (van Kleef, 2009; van Kleef & Côté, 2022). These responses differ depending on group membership: people are more likely to mimic expressions from ingroup members (e.g. Hess et al., 2022; van der Schalk et al., 2011) and to express more negative emotions towards outgroup members (Smith & Mackie, 2015). Further, emotional (vs. neutral) depictions of migrants have been shown to elicit stronger empathic reactions (Bobowik et al., 2023), highlighting the importance of understanding how emotional cues function in intergroup contexts.

Smiling, in particular, tends to evoke positive self‐focused emotions via facial mimicry (Hess & Fischer, 2013). It is among the most effective nonverbal signals for triggering expressions of happiness in others (Patterson et al., 2002). Affiliative smiles function to foster liking and trust (Martin et al., 2017; Rychlowska et al., 2017; van Kleef & Côté, 2022), and people generally report greater liking for individuals who smile (Belkin & Rothman, 2017; Oosterhof & Todorov, 2009; Scharlemann et al., 2001).

Smiles can also elicit other‐focused emotional responses—those rooted in feelings of social connection (Stellar et al., 2017; Yaden et al., 2017). Through appraisals of shared humanity or communal bonds, smiles can foster emotions like *kama muta*, a positive, sudden feeling of being moved or touched by closeness (Fiske et al., 2019). This emotion, often experienced as warmth in the chest, tears or goosebumps, is frequently reported when witnessing expressions of kindness or affection (e.g. Zickfeld et al., 2019), which might include smiling. Smiles may activate relational models based on solidarity and equality, promoting belonging and trust.

In parallel, smiles can be appraised hierarchically—as signals of prestige or competence (e.g. Witkower et al., 2020)—evoking admiration or elevation (Onu et al., 2016). Elevation can also arise in response to perceived moral beauty, kindness or love (Janicke & Oliver, 2017), often conveyed through smiling. Thus, to the extent that host culture members feel a sense of connection with immigrants, a smile may elicit emotions such as admiration or *kama muta* (Lizarazo‐Pereira et al., 2022).

Finally, smiling may help mitigate negative emotional responses such as anger. By eliciting emotional mimicry and facial feedback, positive expressions like smiling can foster more positive emotional states in observers and reduce hostility (Hess & Fischer, 2013). Smiling may also counteract negative stereotypes commonly associated with immigrants. According to the Stereotype Content Model, immigrants are often perceived as low in warmth and high in competitiveness—traits that can elicit anger (Fiske et al., 2002). A smile may reduce perceived threat or competition, thereby lowering anger. Supporting this, Penton‐Voak et al. (2013) found that perceiving happiness in ambiguous facial expressions led to reduced self‐reported anger.

### Smiling and behavioural intentions

Smiling, a widely recognized catalyst for affiliative behaviour in cooperative settings (van Kleef, 2009; van Kleef & Côté, 2022), may play a crucial role in shaping interactions between immigrants and host culture members. People generally respond more favourably to happy expressions, approaching smiling individuals more quickly than those displaying anger (Nikitin & Freund, 2019; Seidel et al., 2010; Stins et al., 2011). Smilers are also more likely to elicit cooperative responses (Krumhuber et al., 2007; Mussel et al., 2013; Reed et al., 2012; Scharlemann et al., 2001) and are often preferred in roles that depend on trust and rapport, such as healthcare professionals (Hall et al., 2020) and co‐workers (Bernstein et al., 2010). Additionally, smiling has been shown to enhance prosocial behaviour, including helping (Guéguen & De Gail, 2003; Vrugt & Vet, 2009), yet research on its impact on helping intentions is still limited.

### The role of group membership

While smiling typically signals positive intent, it remains uncertain whether such expressions elicit the same psychological responses when displayed by immigrants as when expressed by members of the host culture. We propose that smiles from immigrant expressers may have a stronger impact on cognitive inferences, emotional reactions and affiliative behavioural intentions than smiles from host culture expressers. This hypothesis builds on the idea that smiling immigrants convey stereotype‐incongruent information—disconfirming dominant narratives that associate immigrant or minority group members with threat, coldness or social distance (Beaupré & Hess, 2003; Bijlstra et al., 2010). Such incongruent cues can disrupt automatic categorization and promote individuated processing, a mechanism shown to reduce prejudice (e.g. Crisp & Nicel, 2004; Dasgupta & Greenwald, 2001). For instance, Kubota and Ito (2014) found that when a facial expression (smiling or angry) was paired with a racial cue, smiling faces reduced implicit stereotyping, suggesting that especially positive emotional cues can attenuate automatic bias.

Several studies suggest that positive emotional expressions can attenuate the impact of group membership on social perception and responses to others, often producing stronger effects for outgroup than ingroup members. Happy expressions can override group‐based inferences under certain conditions (Ambady & Rosenthal, 1992; Matsumoto & Kudoh, 1993). For instance, Senft et al. (2016) found that smiling had a greater impact on impressions of extraversion and agreeableness for individuals who were perceived more negatively on these traits when inexpressive. White men, for example, were rated as significantly less agreeable than most other targets when inexpressive, but their ratings increased more than others' when smiling. Similarly, Japanese women were perceived as significantly less extrovert when inexpressive and smiling had a larger positive impact on their extraversion ratings compared to other groups. Hareli et al. (2009) showed that expressions of both anger and happiness increased perceived dominance for women compared to inexpressive faces, suggesting that both counter‐stereotypical (anger), but also stereotypical (happiness), emotional displays can amplify or shift stereotype‐inconsistent trait attributions. In Matsumoto and Kudoh's (1993) study, smiling by Japanese expressers led to higher ratings of sociability from US‐born participants—traits that may be seen as stereotype‐inconsistent—and these effects were descriptively stronger than for US‐born expressers. Similarly, Cooley et al. (2018) found that smiles increased perceived femininity and positive expectations for interaction for faces of Black women more than White women, suggesting that emotional expressions can shift perceivers' focus from ethnicity to other social categories like gender.

Beyond impression formation, emotional expressions by outgroup members can influence affiliative behaviour. Yabar and Hess (2007) found that empathic facial expressions elicited stronger approach tendencies towards outgroup members than ingroup members. Zickfeld et al. (2021) reported that tearful expressions had slightly stronger effects on social support intentions for perceived outgroup members.

However, some studies suggest that positive expressions by outgroup members may not elicit the same positive responses as those from ingroup members. The social message account (Weisbuch & Ambady, 2008) proposes that emotional expressions are interpreted through the lens of group membership. A smile from an ingroup member is generally read as affiliative, facilitating approach, while the same expression from an outgroup member may be interpreted as signalling dominance or superiority, leading to avoidance (Gurbuz et al., 2024; Paulus & Wentura, 2014)—although some replications found only independent effects of group membership and emotion (Paulus & Wentura, 2018).

Our approach diverges from this latter work by emphasizing the potential of smiles as counter‐stereotypical social messages from immigrant expressers. Rather than assuming that outgroup smiles are inevitably filtered and interpreted through hostile or dominance‐related stereotypes, we propose that they may instead function as cues that disconfirm negative expectations and shift attention towards shared humanity or positive individual traits and will have stronger effects on perceptions, emotions and behavioural intentions than smiles from host culture expressers.

### Explanatory mechanisms

We proposed that cognitive and emotional reactions to smiling would explain its effects on social interaction and helping intentions. In line with the EASI model (van Kleef, 2009), cognitive inferences and affective reactions to emotion expression are expected to positively correlate with a greater inclination to respond prosocially.

Perceptions of warmth and competence are integral to fostering positive intergroup behaviours (see BIAS Map Model; Cuddy et al., 2007). Evidence indicates that attributing warmth and competence to immigrants correlates with facilitative behaviours such as helping and cooperation (e.g. Becker et al., 2019). Positive emotions, in parallel, expand our behavioural repertoire, enhancing the diversity of actions (Fredrickson, 2013). These emotions, encompassing self‐directed joy, liking, *kama muta* and admiration, are theorized to serve an affiliative function, fostering social connection. The BIAS Model reinforces this viewpoint, indicating that positive emotions drive facilitative behaviours (Cuddy et al., 2007). For instance, feeling joy might ameliorate intergroup biases (Johnson & Fredrickson, 2005) and even improve outgroup‐directed attitudes (Pittinsky & Montoya, 2016). Particularly, self‐transcendent emotions are believed to be key in nurturing relationships and guiding cooperation and caregiving behaviours (e.g. Stellar et al., 2017). Focusing on *kama muta* (or compassion), research indicates its effectiveness in reducing prejudice and promoting prosocial behaviour towards outgroups, including immigrants (e.g. Alonso‐Arbiol et al., 2024; Bobowik et al., 2023; Lizarazo‐Pereira et al., 2022). Similarly, admiration has been identified as a key emotion in fostering affiliative intergroup behaviour and favourable attitudes towards diverse groups (Alonso‐Arbiol et al., 2024; Oliver et al., 2015; Seger et al., 2017).

### Overview of the present research

Across four experiments using five samples, from Spain and the United States, we tested whether smiling influences cognitive (perceived warmth and competence), affective (felt joy, liking, admiration, feeling moved—*kama muta*— and reduced anger) and behavioural responses (approach and avoidance tendencies, and —in two out of four studies—donation and volunteering intentions) towards immigrants with different ethnocultural backgrounds who are already settled in the host culture. In two of the four studies, we also compare the strength of these effects with the effects of similar expressions by host culture members. All hypotheses are summarized in Table 1.

**TABLE 1:** Predictions across Studies 1–4.

Hypothesis	Expected effect	Study
Main effects		
H1	Smiling individuals will be perceived more positively than non‐smiling individuals, reflected in higher ratings of warmth and competence.	Studies 1–4
H2a	Smiling individuals will elicit stronger positive emotional responses—specifically, greater joy, liking, admiration, and feeling moved (kama muta)—compared to non‐smiling individuals.	Studies 1–4
H2b	Smiling individuals will elicit less anger than non‐smiling individuals.	Studies 1–4
H3	Smiling individuals will evoke greater affiliative intentions—namely, increased approach and helping intentions, and reduced avoidance intentions—compared to non‐smiling individuals.	Studies 1–4
Interaction effects		
H4	The positive impact of smiling on perceived warmth and competence will be stronger for immigrant expressers than for host‐culture expresser.	Studies 3–4
H5	The emotional effects of smiling (increased admiration, kama muta, and liking; reduced anger) will be stronger for immigrant expresser than for host‐culture expresser.	Studies 3–4
H6	The effect of smiling on affiliative intentions (approach vs. avoidance and helping) will be stronger for immigrant expressers than for host‐culture expresser.	Studies 3–4
Indirect effects		
H7	The relationship between smiling and affiliative intentions will be mediated by perceptions of warmth and competence.	Studies 1–4
H8	The relationship between smiling and affiliative intentions will also be mediated by emotional responses—stronger positive emotions (admiration, kama muta, liking) and reduced anger.	Studies 1–4
H9	These indirect effects will be moderated by group membership; that is, the strength of the mediation pathways will vary depending on whether the expresser is an immigrant or from the host culture.	Studies 3–4

Study 1 was the first preliminary study in which we compared the effects of smiling on an observer's responses towards a Senegalese immigrant man with three other emotional displays (neutral, angry and sad expression). In Studies 2a and 2b, we focused specifically on the effects of smiling (vs. a neutral display) by a Moroccan and Pakistani man in Spain and the US, respectively. Considering a relatively small sample size, we analysed the data from these two experiments using the Integrative Data Analysis approach (IDA; Curran & Hussong, 2009; see Analytical Strategy section for more details on this procedure), which allows for controlling for the between‐study heterogeneity and increases the statistical power. In Study 3, we examined the effects of smiling by members of a different disadvantaged ethnocultural group (a Romanian immigrant) compared to a control advantaged ingroup. We additionally measured a wider variety of affective reactions and intentions to help the individual (donating and volunteering intentions). Study 4 was a robust, pre‐registered experiment involving multiple stimuli per each condition, as well as three different groups of immigrants. Finally, we carried out a meta‐analytical integration of our findings, especially to test hypotheses on indirect effects via affective and cognitive mechanisms. These effects were tested using the meta‐analytical integration to ensure sufficient statistical power. Across all studies, we relied on images of men because research suggests that especially outgroup men are perceived as displaying positive emotions less intensively (Beaupré & Hess, 2003). Conversely, women are argued to smile more to signal social intent (Hess & Bourgeois, 2010). These studies were approved by the Research Ethics Committee for Studies Involving Humans of the University of the Basque Country. Informed consent was obtained from all voluntary participants. None of the hypotheses were pre‐registered in Studies 1–3. Study 4 was pre‐registered (https://osf.io/9gh5w/files/dnwf5).

All data and the full reproducible code, as well as supplementary analyses and the questionnaires in English and Spanish are available at the OSF Project website (https://osf.io/9gh5w/). Experimental stimuli are available upon request. The Supplementary Online Material (SOM)  include additional results from each study, such as bivariate correlations and descriptive statistics, detailed results from path analyses, as well as stimuli validation results from Study 4.

## STUDY 1

### Participants

One hundred and twenty‐five native‐born Spanish undergraduate students (all Caucasian; 85.6% identified as women; no non‐binary options were offered in Studies 1–3; *M*_age_ = 21.82, SD = 3.79) filled in a web‐based questionnaire during an introductory social psychology class for course credit. A post hoc sensitivity analysis using G*Power 3.1 (Faul et al., 2009) showed that with *α* = 0.05 and 1−*β* prob. = .80, a total sample of 125 participants was sufficient to detect an effect size of *f* = 0.30 in a one‐way ANOVA with four groups.

### Procedure and materials

Participants were informed that the study examined their opinions about immigration and that their participation was voluntary and anonymous. They were randomly assigned to one of four experimental conditions in which they were exposed to a photograph of a man presented as a Senegalese immigrant with either a neutral, smiling, angry or sad facial expression and read brief contextual information about the person: ‘Mustafa was 27 years old and had been living in Spain for four years’. The emotional stimuli applied in the study were images of the same individual selected by a group of experts from a standardized Montreal Set of Facial Displays of Emotions (Beaupré et al., 2000) as an image representative of a prototypical immigrant from Senegal.

Participants observed the stimulus at their own pace and filled in the questionnaire when ready. The questionnaire covered manipulation checks, measures of perceived warmth and competence, felt emotions and behavioural intentions. All measures, with sample items, response formats and reliabilities are presented in Table 2. Finally, participants reported their socio‐demographic information, were probed for suspicion (none revealed knowledge about the purpose of the experiment), debriefed and thanked for their participation in the study. The procedures involving manipulation checks and the measurement of perceptions of warmth and competence, affective reactions and behavioural intentions were constant across all studies unless indicated otherwise.

**TABLE 2:** Measures across Studies 1−4.

Variable	Items	# Items and Reliability			
		Study 1	Study 2a/2b	Study 3	Study 4
Manipulation Checks: Emotion Expression Response scale: 1 (*not at all*)*–*7 (*a lot*)					
Perceived happiness	‘happiness’, ‘joy’	1 item	1 item	1 item	2 items *r* = 0.92
Perceived neutrality	‘neutral’, ‘no emotion’	NA	1 item	1 item	2 items *r* = 0.55
Perceived anger	‘anger’	1 item	NA	NA	NA
Perceived sadness	‘sadness’	1 item	NA	NA	NA
Manipulation Checks: Group Membership Response scale: *1 (strongly disagree)–7 (strongly agree)*					
Group membership	‘He belongs to the same ethnic or cultural group as me’, ‘He is from the same ethnic or cultural group as me’, ‘He is a member of another ethnic or cultural group than my own’, ‘He is representative of another ethnic or cultural group than my own’	NA	NA	4 items *α* = 0.92	4 items *α* = 0.94
Cognitive inferences Response scale in Studies 1–2: 1 (*not at all*)–10 (*a lot*) Response scale in Studies 3–4: 1 (*not at all*)–7 (*a lot*)					
Warmth	‘warm’ ‘nice’ ‘likeable’	1 item	1 item	3 items *α* = 0.93	3 items *α* = 0.91
Competence	‘competent’ ‘skilled’ ‘intelligent’	1 item	1 item	3 items *α* = 0.77	3items *α* = 0.89
Felt emotions Response scale: 1 (*not at all*)–7 (*a lot*)					
Joy	‘joyful’, ‘interested’	1	2 items *r* = 0.44/ *r* = 0.65	NA	NA
Liking	‘trusting’, ‘liking’	2 items *r* = 0.71	2, items *r* = 0.78/ *r* = 0.92	2 items *r* = 0.79	3 items *α* = 0.92
Anger	‘angry’, ‘outraged’, ‘irritated’	2 items *r* = 0.68	2 items *r* = 0.52/ *r* = 0.46	3 items *α* = 0.87	3 items *α* = 0.94
Admiration	‘admiring’, ‘inspired’, ‘fascinated’	NA	NA	1 item	3 items *α* = 0.94
Feeling moved (‘kama muta’)	‘moved’, ‘touched’, ‘tender‐hearted’	NA	NA	2 items *r* = 0.64	3 items *α* = 0.93
Affiliative intentions Response sale: *1 (strongly disagree)–7 (strongly agree)*					
Approach intentions	‘I’d like to approach or establish contact with him’, ‘I’d like to feel close to him, talk to him and listen to him’, ‘I would like to spend time with him in a social activity’, ‘I would like to include him in my group of friends or acquaintances’	2 items *r* = 0.90	2 items *r* = 0.87 / *r* = 0.88	2 items *r* = 0.84	4 items *α* = 0.97
Avoidance intentions	‘I feel I should protect myself from him’, ‘I would rather not have anything to do with him’, ‘I would prefer to distance myself from him/to keep my distance and not interact with him’, ‘I would avoid being near him in an everyday situation’ In Study 1 items ‘I would prefer not to cross his path’, ‘I would rather not have to take care of him or look after him’ were used instead of the fourth item	5 items *α* = 0.73	2 items *r* = 0.59 / *r* = 0.77	2 items *r* = 0.47	4 items *α* = 0.96
Helping intentions Response sale: 1 (*not willing at all*)–7 (*very much willing*)					
Donation intentions	‘donate money to fund your vocational training’ ‘donate money to financial aid to help him get hired quickly’, ‘donate money to support organizations that fights discrimination against immigrants’, ‘donate to support education scholarships for immigrants’	NA	NA	2 items *r* = 0.81	4 items *α* = 0.93
Volunteering intentions	‘help him with bank procedures’, ‘help him with official business’, ‘help him to register to facilitate his installation in your city’, ‘accompany him to a job interview’, ‘participate in mentorship programs to help him adapt to his new environment’	NA	NA	3 items *α* = 0.92	5 items *α* = 0.95

*Note:* All correlations presented are *p* < .01.

### Analytical strategy

We conducted one‐way analysis of variance (ANOVA) with Tukey's HSD post hoc test to analyse the effects of smiling on the manipulation checks and the dependent variables (i.e. cognitive inferences, emotions and behavioural intentions). Next, we tested the indirect effects of experimental manipulation on behavioural intentions via observer's inferences and emotions, using path analyses and bias‐corrected bootstrapping with 10,000 samples for indirect effect confidence intervals. We tested the role of cognitive and affective mechanisms in independent mediation models. All analyses across studies were conducted in R (R Core Team, 2014) through RStudio (RStudio Team, 2015), using the packages *lavaan* (Rosseel, 2014) and *apaTables* (Stanley, 2018).

### Results

#### Descriptive findings and manipulation checks

Correlations among variables, as well as their means and standard deviations, can be found in SOM, in Table S1.1. ANOVA tests revealed that participants attributed more happiness and less sadness to the expresser with a happy display compared to the neutral, angry and sad displays (Table 3). Therefore, we considered the manipulation successful.

**TABLE 3:** Means, standard deviations and ANOVAS results: Impact of smiling depictions of immigrants on host culture members' cognitive inferences, affective reactions and behavioural intentions (Study 1, *N* = 125).

Variable	Condition				Comparison		
	Neutral display *M* (SD)	Smiling display *M* (SD)	Angry display *M* (SD)	Sad display *M* (SD)	*F*_(3,121)_	*p*	$\eta_{p}^{2}$ [90% CI]
Manipulation check							
Happiness	1.51 (0.85)^a^	5.26 (1.39)^b^	1.35 (0.71)^a^	1.31 (0.64)^a^	114.25	<.001	0.75 [0.67, 0.79]
Sadness	5.89 (1.13)^c^	2.12 (1.09)^a^	3.39 (1.54)^b^	6.03 (1.22)^c^	68.39	<.001	0.63 [0.53, 0.68]
Anger	1.34 (0.76)^a^	1.08 (0.28)^a^	4.03 (1.96)^b^	1.32 (0.88)^a^	44.67	<.001	0.53 [0.41, 0.59]
Cognitive inferences							
Warmth	4.69 (2.21)^a^	6.76 (1.36)^b^	3.52 (1.79)^a^	4.18 (1.88)^a^	15.15	<.001	0.27 [0.15, 0.36]
Competence	5.32 (1.61)^a^	6.52 (1.71)^b^	5.03 (1.45)^a^	4.85 (1.76)^a^	5.70	.001	0.12 [0.03, 0.20]
Affective reactions							
Joy	1.71 (1.23)^a^	4.96 (1.43)^b^	2.00 (1.22)^a^	1.38 (0.74)^a^	43.10	<.001	0.52 [0.41, 0.59]
Liking	3.70 (1.43)^a^	5.46 (1.22)^b^	2.95 (1.46)^a^	3.06 (1.34)^a^	19.23	<.001	0.32 [0.20, 0.41]
Anger	1.69 (1.34)^a,b^	1.02 (0.10)^a^	2.15 (1.27)^b^	1.88 (1.29)^b^	4.62	.004	0.10 [0.02, 0.18]
Behavioral intentions							
Approach	5.06 (1.67)	5.01 (1.45)	4.83 (1.55)	4.25 (1.48)	2.74	.046	0.06 [0.00, 0.13]
Avoidance	1.25 (0.43)	1.18 (0.39)	1.37 (0.61)	1.47 (0.65)	1.74	.163	0.04 [0.00, 0.09]

*Note:* Approach = Approach intentions; Avoidance = Avoidance intentions. *M* (SD) represent means and standard deviations, respectively. $\eta_{p}^{2}$ [90% CI] represent eta squared and the lower‐ and upper‐limit of its confidence interval. Different superscript letters represent differences across conditions (*p* < .05), based on Tukey's HSD tests. Number of participants: Neutral: *n* = 35; Smile: *n =* 25; Anger: *n =* 31; Sadness: *n =* 34.

#### Main effects

**FIGURE 1:**
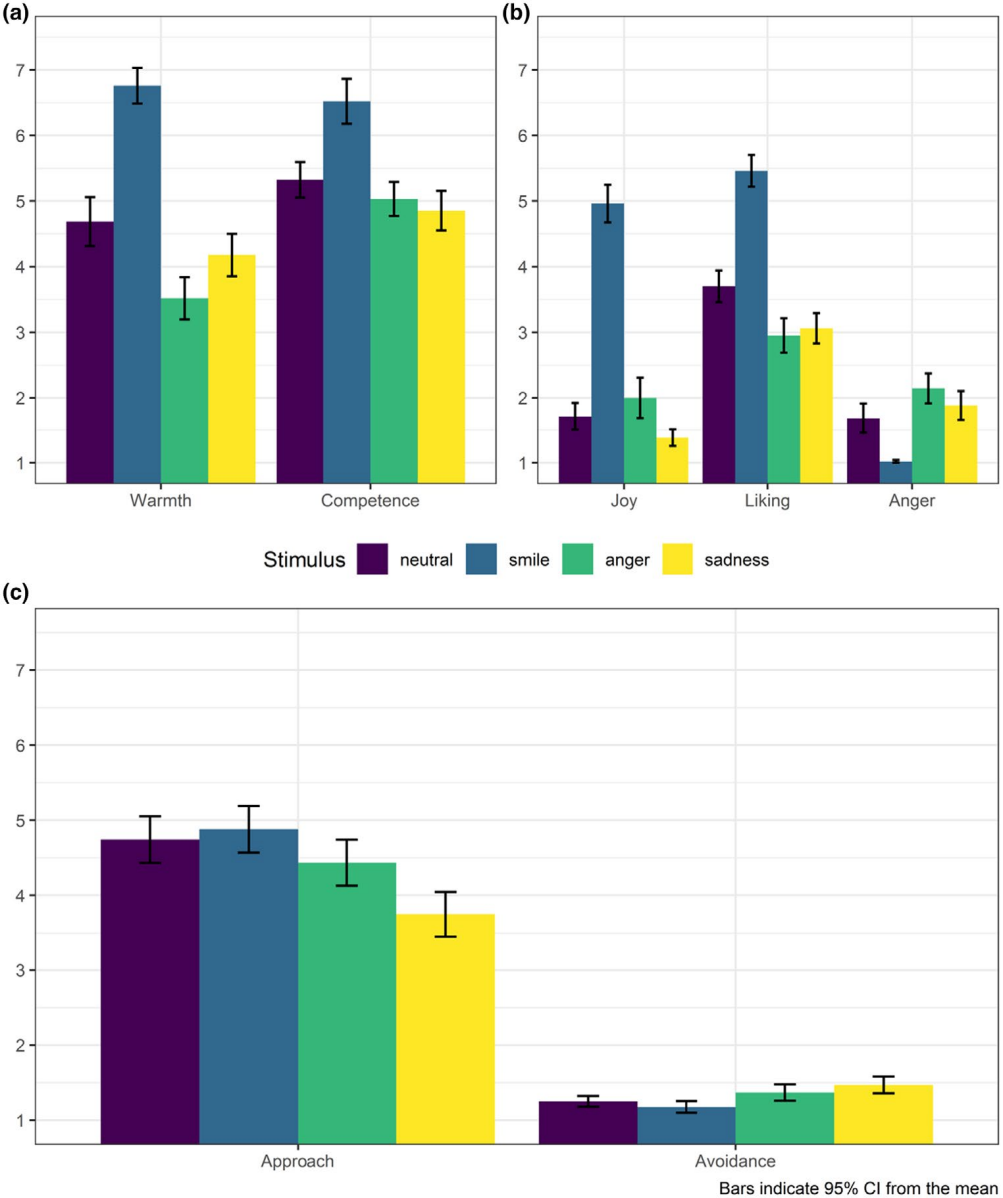
Impact of smiling depictions of immigrants on host culture members' cognitive inferences, affective reactions and behavioural intentions (Study 1, *N* = 125).

The analyses revealed that participants rated the smiling individual warmer and more competent (Figure 1a), as well as indicated having felt less anger, and more joy and liking towards him (Figure 1b), compared to those in the remaining conditions. We did not observe significant differences between the conditions in their behavioural intentions (Figure 1c). All statistics are reported in Table 3.

#### Indirect effects

Smiling increased approach intentions indirectly via perceived warmth (*B* = 0.71, SE = 0.32, *p* = .027, 95% CI [0.08, 1.35]) and felt liking (*B* = 1.46, SE = 0.40, *p* < .001, 95% CI [0.69, 2.23]), but not through competence or other emotions. No significant indirect effects emerged for avoidance intentions (see Tables S1.2 and S1.3 in SOM for complete path analysis results). These effects warrant cautious interpretation due to the limited sample size (see Table 8 for the meta‐analytical integration of indirect effects).

## STUDY 2a AND 2b

In Study 2, we analysed responses to smiling faces of immigrant men across two contexts: Spain (Study 2a) and the United States (Study 2b). In Spain, we chose Moroccans as a target immigrant group because they are evaluated lower on warmth and competence compared to other immigrants (López‐Rodríguez et al., 2014). In the United States, we used South Asian (Pakistani) immigrants as a group of interest, also because Muslim migrants are a group perceived as threatening in the United States (Fiske et al., 2002).

### Participants

In Study 2a, 88 native‐born Spanish undergraduate students (all Caucasian; 83% identified as women; *M*_age_ = 22.80, SD = 1.66) filled in a web‐based questionnaire during an introductory social psychology class for course credit. In Study 2b, participants were 63 US‐born Mturk workers (59% identified as women, *M*_age_ = 36.35, SD = 12.74). Data from Study 2a and 2b were analysed in an integrative manner (IDA; Curran & Hussong, 2009; see Analytical Strategy section for more details on this procedure). A post hoc sensitivity analysis in G*Power 3.1 (Faul et al., 2009) indicated that a total sample of 151 participants was enough to detect a medium effect size (*f* = 0.33), considering *α* prob. = .05 and 1−*β* prob. = .80 in a 2 × 2 analysis of variance.

### Procedure and materials

We randomly assigned participants to observe an image of a Moroccan/Pakistani man with either a happy (smiling) or a neutral facial expression, presented as a person who had been living in Spain/the US for the last couple of years. In Study 2a, we used natural photographs of a Moroccan collaborator that we took for this study.^1^ The emotional stimuli applied in Study 2b were images from the Radboud Faces Database (Langner et al., 2010).^2^ After observing the photographs at their own pace, participants filled in an online survey. All measures, with sample items, response formats and reliabilities are presented in Table 2.

### Analytical strategy

To examine the effects of smiling on dependent variables while controlling for the experiment and considering small sample sizes across experiments, we used the Integrative Data Analysis (Curran & Hussong, 2009). This strategy provides several advantages—most notably greater statistical power—and has been recently widely used in social psychological research (e.g. Teixeira et al., 2020). It also controls for between‐study heterogeneity resulting from characteristics unique to each study, such as differences in sampling techniques, design or measurement tools, thus testing the generalizability and reliability of findings. For this purpose, the effects of assignment in each study and its interactions with the rest of the independent variables are additionally estimated in the model. Specifically, we conducted two‐way ANOVAs with the smiling (vs. neutral) display and the experiment (Study 2a vs. Study 2b) as two independent variables and an interaction effect between the two factors. We tested indirect using multi‐group path analyses and indirect effects as in Study 1.

### Results

#### Descriptive findings and manipulation checks

Correlations, means and standard deviations across both experiments can be found in SOM (Tables S2.1 and S2.2). The ANOVA showed that the smiling expresser was perceived as happier and less neutral than the non‐smiling expresser, and we therefore considered our experimental manipulation successful (Table 4). We did not observe significant interaction effects, which indicate that the effect of manipulation was comparable across both samples. There was a small main effect of the experiment on perceived neutrality of the expression, with the Moroccan expresser perceived as overall less neutral compared to the Pakistani expresser.

**TABLE 4:** Means, standard deviations and ANOVAs results: Impact of smiling depictions of immigrants on host culture members‘ cognitive inferences, affective reactions and behavioural intentions (Study 2ab, *N* = 151).

Variable	Study	Stimulus		Comparison			
		Non‐smiling display	Smiling display	Test^a^	*F*_(1,145)_	*p*	$\eta_{p}^{2}$ [90% CI]
		*M* (SD)	*M*(SD)				
Manipulation check							
Neutral	Study 2a	4.20 (1.65)	1.87 (1.41)	SE	4.55	.035	0.03 [0.00, 0.09]
				CE	98.28	<.001	0.40 [0.30, 0.49]
	Study 2b	5.07 (1.87)	2.16 (1.42)	IE	1.23	.270	0.01 [0.00, 0.05]
			
Happy	Study 2a	2.02 (1.00)	5.64 (1.34)	SE	0.35	.553	0.00 [0.00, 0.03]
				CE	439.16	<.001	0.75 [0.70, 0.79]
	Study 2b	1.63 (0.96)	5.78 (1.01)	IE	2.07	.152	0.01 [0.00, 0.06]
			
Cognitive Inferences							
Warmth	Study 2a	3.40 (1.81)	6.10 (1.75)	SE	15.89	<.001	0.10 [0.03, 0.18]
				CE	115.44	<.001	0.44 [.34, .52]
	Study 2b	4.07 (2.07)	7.70 (1.38)	IE	2.52	.115	0.02 [0.00, 0.07]
			
Competence	Study 2a	4.72 (1.95)	5.48 (2.06)	SE	24.03	<.001	0.14 [0.06, 0.23]
				CE	16.85	<.001	0.10 [0.04, 0.18]
	Study 2b	5.60 (2.16)	7.67 (1.51)	IE	4.16	.043	0.03 [0.00, 0.08]
			
Affective Reactions							
Joy	Study 2a	2.25 (1.03)	3.74 (1.46)	SE	0.52	.473	0.00 [0.00, 0.04]
				CE	62.47	<.001	0.30 [0.20, 0.39]
	Study 2b	2.13 (1.09)	4.14 (1.59)	IE	1.38	.242	0.01 [0.00, 0.05]
			
Liking	Study 2a	2.74 (1.39)	4.52 (1.41)	SE	1.31	.254	0.01 [0.00, 0.05]
				CE	77.65	<.001	0.35 [0.24, 0.43]
	Study 2b	2.13 (1.40)	4.55 (1.50)	IE	1.78	.184	0.01 [0.00, 0.06]
			
Anger	Study 2a	1.30 (0.67)	1.20 (0.58)	SE	1.86	.175	0.01 [0.00, 0.06]
				CE	0.90	.344	0.01 [0.00, 0.04]
	Study 2b	1.47 (0.81)	1.35 (0.80)	IE	0.00	.945	0.00 [0.00, 1.00]
			
Behavioural intentions							
Approach	Study 2a	2.64 (1.10)	3.09 (1.56)	SE	5.51	.020	0.04 [0.00, 0.10]
				CE	12.11	.001	0.08 [0.02, 0.15]
	Study 2b	2.73 (1.63)	4.06 (1.46)	IE	3.32	.071	0.02 [0.00, 0.07]
			
Avoidance	Study 2a	2.26 (1.32)	1.53 (0.82)	SE	0.95	.332	0.01 [0.00, 0.04]
				CE	8.86	.003	0.06 [0.01, 0.13]
	Study 2b	2.35 (1.60)	1.85 (1.55)	IE	0.28	.596	0.00 [0.00, 0.03]
			

*Note*: Approach = Approach intentions; Avoidance = Avoidance intentions. Study 1a: a Moroccan man (Spain); Study 1b: a Pakistani man (the US). *M* (SD) represent means and standard deviations, respectively. $\eta_{p}^{2}$ [90% CI] represent eta squared and the lower‐ and upper‐limit of its confidence interval.

^a^ The ANOVA contrasts: SE = Study effects; CE = Condition effects; and IE = Interaction effects (Study * Condition). Number of participants by study and condition: Study 2a‐Neutral: *n* = 40; Study 2a‐Smile: *n* = 48; Study 2b‐Neutral: *n* = 30; Study 2b‐Smile: *n* = 33.

#### Main and interaction effects

**FIGURE 2:**
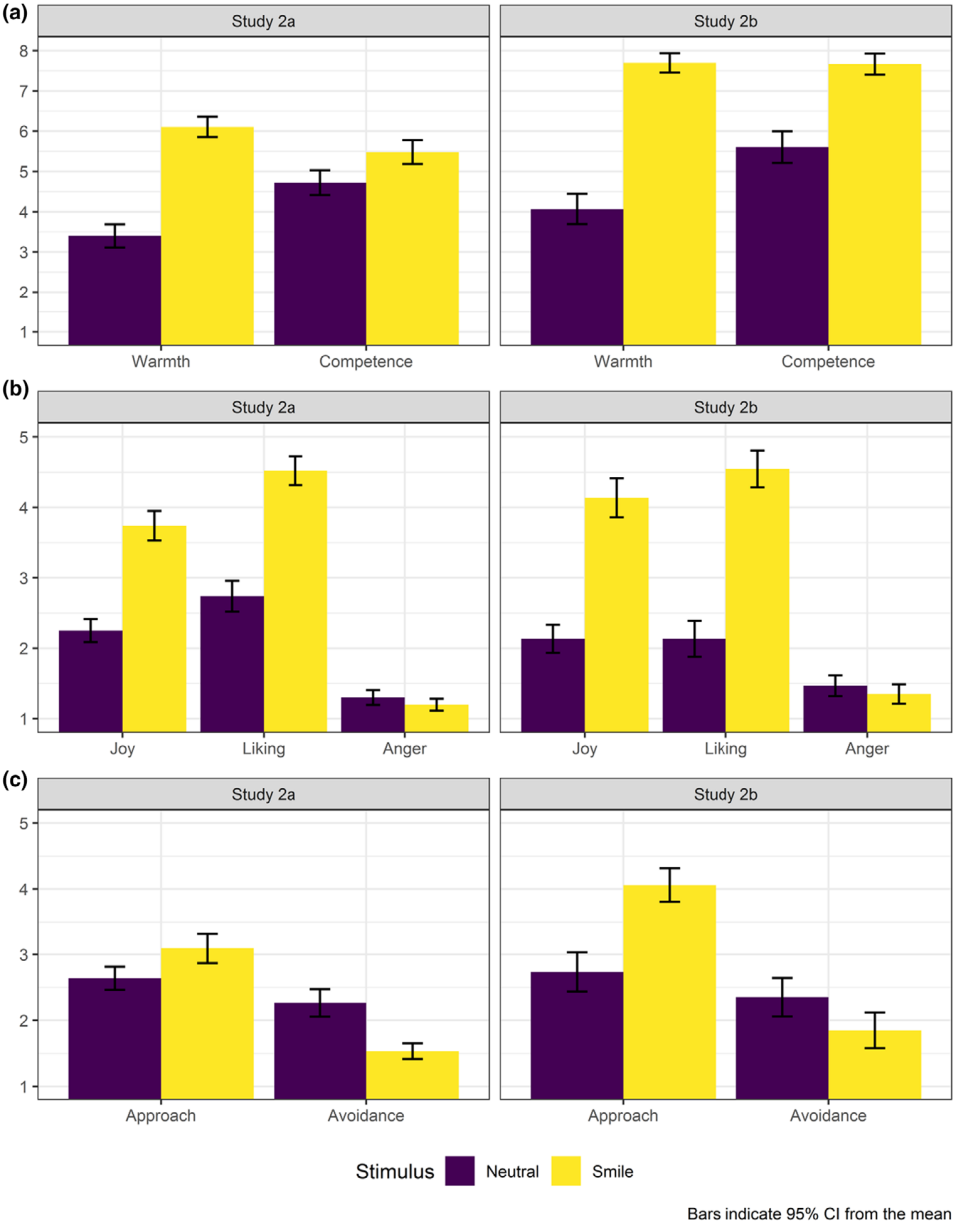
Impact of smiling depictions of immigrants on host culture members' cognitive inferences, affective reactions and behavioural intentions (Study 2ab, *N* = 151).

Compared with the non‐smiling display, participants rated the smiling expresser as warmer and as more competent (Figure 2a). In addition, participants felt more joy and liking (but not less anger, Figure 2b), more intentions to engage in interaction and fewer intentions to avoid contact (Figure 2c) with the smiling versus non‐smiling expresser. Additionally, we observed a significant interaction effect only in the case of perceived competence. When comparing the two samples, the effect of smiling on perceptions of competence was statistically significant only in the case of the Pakistani expresser, whereas it was in the expected direction yet did not reach statistical significance in the case of the Moroccan expresser (Figure 2a).

#### Indirect effects

We observed statistically significant indirect effects of smiling on approach intentions via perceived warmth (Study 2a: *B* = 0.71, SE = 0.25, *p* = .005, 95% CI [0.21, 1.21]; non‐significant in Study 2b due to limited statistical power) and felt liking (Study 2a: *B* = 0.83, SE = 0.27, *p* = .002, 95% CI [0.30, 1.36]; Study 2b: *B* = 1.46, SE = 0.46, *p* < .001, 95% CI [0.57, 2.35]). The indirect effects via perceived competence or other emotions were not statistically significant.

Significant indirect effects of smiling on avoidance intentions were also observed via warmth (Study 2b only; *B* = −1.38, SE = 0.55, *p* = .012, 95% CI [−2.45, −0.31]) and via felt liking (Study 2a: *B* = −0.67, SE = 0.25, *p* = .008, 95% CI [−1.16, −0.18]; Study 2b: *B* = −1.32, SE = 0.47, *p* = .005, 95% CI [−2.25, −0.40]). The indirect effects via perceived competence or other emotions were not statistically significant (see Tables S2.3 and S2.4 in SOM for complete path analyses).

Replicating Study 1, smiling again increased approach intentions via perceived warmth and felt liking. It also newly reduced avoidance intentions through these same mediators, while effects via competence or other emotions remained non‐significant. These effects should be interpreted with caution given the limited sample size (see Table 8 for the meta‐analytical integration of indirect effects).

## STUDY 3

One possible source of uncertainty in Study 2ab is the lack of a group category control condition. Therefore, it is unclear whether the effects of smiling among members of socially disadvantaged ethnocultural groups differ from the effects of smiling by ingroup members. In Study 3, using a 2 by 2 ANOVA design (smiling by group membership), we compared the effects of smiling by members of one's group and by immigrants. We additionally measured helping intentions: disposition to make a monetary donation and to volunteer time in an organization that helps immigrants. We also examined the impact of smiling on a broader array of emotions, including *kama muta* and admiration. Finally, we ensured the reliability of warmth and competence by using multiple‐item scales and included an a priori power analysis to estimate the desired sample size.

### Participants

We recruited 169 native‐born Spanish adults (66.9% identified as women, *M*_age_ = 23.66, SD = 8.82) through social media. To establish the desired sample size, we conducted a power analysis with G*Power 3.1 (Faul et al., 2009), considering the results of Study 1, those of Study 2a and 2b and added an independent study (Krys et al., 2014). Weighing the effects (i.e. effects of smiling on warmth and competence) and considering the extended practices (i.e. alpha at 0.05 and 1‐*β* prob. = .95), the analysis revealed that we would need a sample of 17 participants per group to achieve an effect on warmth of *d* = 1.29 (i.e. *f* = 0.64) and 41 per group for the effect on competence of *d* = 0.81 (i.e. *f* = 0.40). For that reason, we decided to follow the criteria for competence (*N* = 164) and stop the data gathering once this number was surpassed.

### Procedure and materials

Participants were invited to fill in a web‐based questionnaire and randomly assigned to a 2 (stimulus: neutral vs. smile) by 2 (group: ingroup vs. outgroup) between‐subject factorial design, where they were asked to observe at their own pace a photograph of a Caucasian man. We applied images of the same individual extracted from a standardized Karolinska Directed Emotional Faces dataset (Lundqvist et al., 1998).^3^ In the context of Spain, it was possible to frame the same photograph of a Caucasian men as an image of an immigrant and non‐immigrant (see Stürmer et al., 2006; van der Schalk et al., 2011 for similar procedures). The following description accompanied the photograph: ‘Pablo is from the same province as you/Pablo is from Romania. He has just moved to the city where you live and is settling down’. Afterwards, participants were asked to fill in a series of measures. All measures, with sample items, response formats and reliabilities are presented in Table 2.

### Analytical strategy data analyses

We conducted two‐way ANOVAs to examine the effects of group membership and smiling on dependent variables, following the analytical strategy from Study 2. Indirect effects were also tested as in Study 2, using multi‐group path analyses.

### Results

#### Descriptive findings and manipulation checks

All correlations among variables, as well as means and standard deviations, can be found in Tables S3.1–S3.3 in SOM. The ingroup member was perceived as more belonging to one's group than the outgroup member (i.e. the Romanian man, see Table 5). As in Studies 1 and 2ab, the smiling display activated more attributions of happiness and was perceived as less neutral than the non‐smiling expression.

**TABLE 5:** Means, standard deviations and ANOVAs results: Impact of smiling depictions of immigrants (outgroup) versus non‐immigrants (ingroup) on host culture members‘ cognitive inferences, affective reactions and behavioural intentions (Study 3, *N* = 169).

Variable	Group	Stimulus		Comparison			
		Non‐smiling display	Smiling display	Test^a^	*F*_(1,165)_	*p*	$\eta_{p}^{2}$ [90% CI]
		*M* (SD)	*M* (SD)				
Manipulation check							
Group Membership	Ingroup	4.92 (1.60)	5.19 (1.22)	GE	16.21	<.001	0.09 [0.03, 0.16]
				CE	1.13	.289	0.01 [0.00, 0.04]
	Outgroup	4.00 (1.66)	4.23 (1.48)	IE	0.01	.940	0.00 [0.00, 0.01]
			
Neutral	Ingroup	4.87 (1.58)	2.14 (1.49)	GE	0.09	.760	0.00 [0.00, 0.02]
				CE	118.71	<.001	0.42 [0.32, 0.50]
	Outgroup	4.74 (1.73)	2.12 (1.52)	IE	0.05	.816	0.00 [0.00, 0.02]
			
Happy	Ingroup	1.80 (1.01)	6.03 (1.09)	GE	0.08	.775	0.00 [0.00, 0.02]
				CE	794.07	<.001	0.83 [0.79, 0.85]
	Outgroup	1.72 (1.00)	6.22 (0.91)	IE	0.79	.377	0.00 [0.00, 0.04]
			
Cognitive inferences							
Warmth	Ingroup	3.23 (1.01)	5.20 (0.86)	GE	4.26	.041	0.03 [0.00, 0.08]
				CE	194.33	<.001	0.54 [0.46, 0.61]
	Outgroup	3.43 (1.08)	5.63 (0.88)	IE	0.55	.458	0.00 [0.00, 0.03]
			
Competence	Ingroup	4.05 (0.85)	4.21 (0.93)	GE	5.12	.025	0.03 [0.00, 0.08]
				CE	11.51	.001	0.07 [0.02, 0.13]
	Outgroup	4.08 (1.10)	4.88 (0.81)	IE	5.00	.027	0.03 [0.00, 0.08]
			
Affective reactions							
Liking	Ingroup	2.38 (1.22)	3.01 (1.31)	GE	5.22	.024	0.03 [0.00, 0.09]
				CE	16.45	<.001	0.09 [0.03, 0.16]
	Outgroup	2.66 (1.07)	3.72 (1.79)	IE	1.00	.319	0.01 [0.00, 0.04]
			
Anger	Ingroup	1.60 (1.01)	1.22 (0.60)	GE	0.00	.980	0.00 [0.00, 1.00]
				CE	10.75	.001	0.06 [0.02, 0.13]
	Outgroup	1.64 (1.05)	1.17 (0.55)	IE	0.12	.731	0.00 [0.00, 0.02]
			
*Kama muta*	Ingroup	2.56 (1.36)	2.35 (1.49)	GE	0.35	.557	0.00 [0.00, 0.03]
				CE	3.72	.055	.02 [0.00, 0.07]
	Outgroup	2.12 (1.22)	3.15 (1.72)	IE	7.57	.007	0.04 [0.01, 0.10]
			
Admiration	Ingroup	2.13 (1.29)	2.54 (1.61)	GE	9.02	.003	0.05 [0.01, 0.12]
				CE	16.07	.001	0.09 [0.03, 0.16]
	Outgroup	3.35 (1.65)	3.95 (2.02)	IE	5.47	.021	0.03 [0.00, 0.09]
			
Behavioural intentions							
Approach	Ingroup	3.41 (1.35)	3.76 (1.46)	GE	11.36	.001	0.06 [0.02, 0.13]
				CE	10.81	.001	0.06 [0.02, 0.13]
	Outgroup	3.82 (1.53)	4.91 (1.45)	IE	2.84	.094	0.02 [0.00, 0.06]
			
Avoidance	Ingroup	2.21 (1.12)	1.69 (0.90)	GE	2.71	.102	0.02 [0.00, 0.06]
				CE	12.28	.001	0.07 [0.02, 0.14]
	Outgroup	1.98 (1.23)	1.40 (0.66)	IE	0.03	.864	0.00 [0.00, 0.01]
			
Donation	Ingroup	2.93 (1.52)	2.78 (1.65)	GE	0.00	.999	0.00 [0.00, 1.00]
				CE	0.03	.874	0.00 [0.00, 0.01]
	Outgroup	2.76 (1.50)	2.98 (1.49)	IE	0.59	.444	0.00 [0.00, 0.03]
			
Volunteering	Ingroup	3.97 (1.72)	4.02 (1.94)	GE	1.69	.196	0.01 [0.00, 0.05]
				CE	3.23	.074	0.02 [0.00, 0.07]
	Outgroup	3.92 (1.59)	4.82 (1.59)	IE	2.46	.119	0.02 [0.00, 0.06]
			

*Note*: Approach = Approach intentions; Avoidance = Avoidance intentions; Donation = Donation intentions; Volunteering = Volunteering intentions; Ingroup = the Spanish man; Outgroup = the Romanian man. *M* (SD) represents means and standard deviations, respectively. $\eta_{p}^{2}$ [90% CI] represents eta squared and the lower‐ and upper‐limit of its confidence interval.

^a^ The ANOVA contrasts: GE = Group membership effects; CE = Condition effects (smiling vs. non‐smiling display); and IE = Interaction effects (Group membership * Condition). Number of participants by condition: Neutral‐Ingroup: *n* = 45; Neutral‐Outgroup: *n* = 46; Smile‐Ingroup: *n* = 37; Smile‐Outgroup: *n* = 41.

#### Main and interaction effects

**FIGURE 3:**
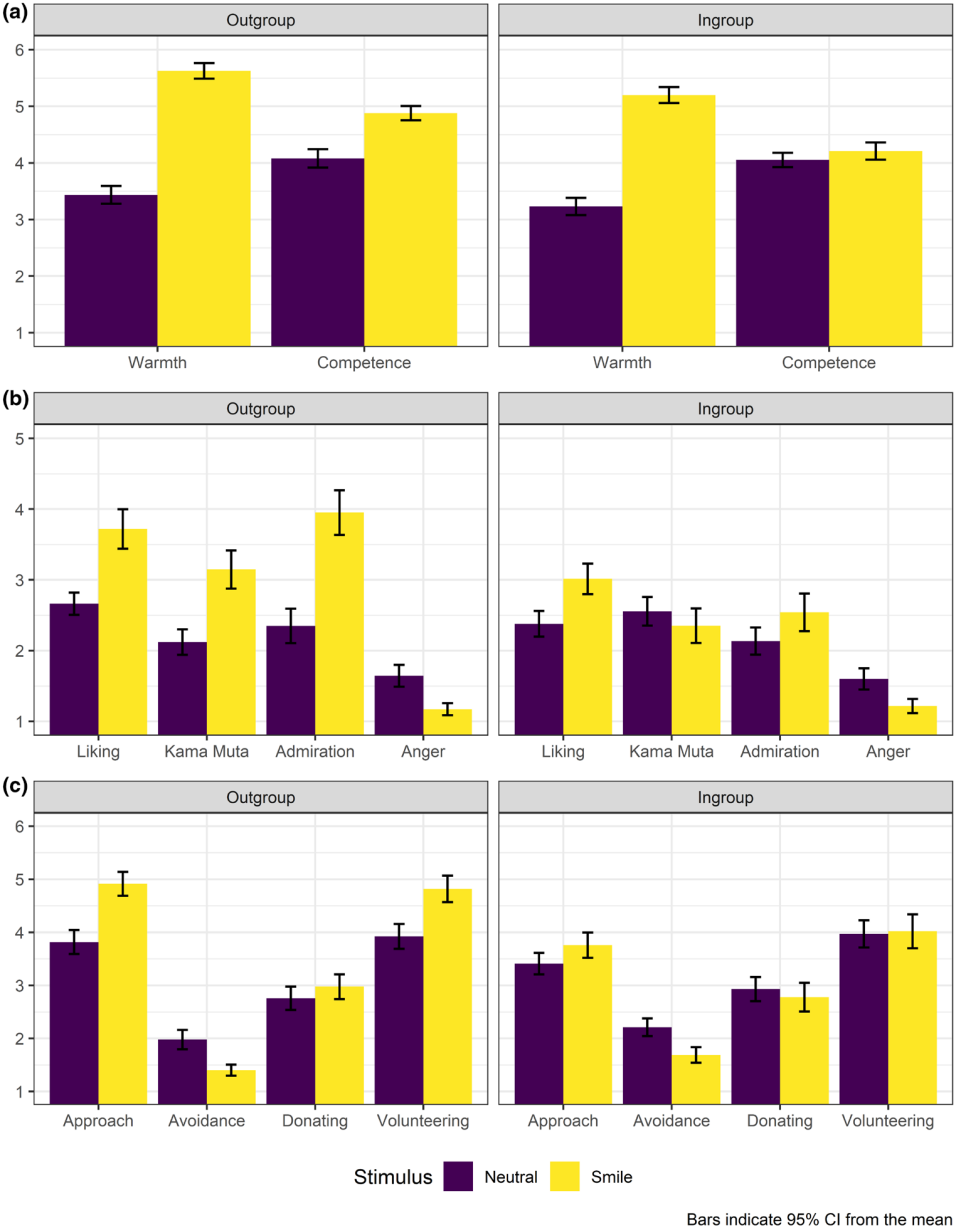
Means, standard deviations and ANOVA results: impact of smiling depictions of immigrants (outgroup) versus non‐immigrants (ingroup) on host culture members' cognitive inferences, affective reactions and behavioural intentions (Study 3, *N* = 169).

The two‐way ANOVA results revealed the main effects of smiling display (vs. the neutral one) and group membership on inferences of warmth and competence. Participants perceived the smiling expresser as warmer and more competent than the expresser with a non‐smiling display. These evaluations were also higher for the Romanian expresser than for the ingroup member. In the case of perceived competence, we also found a statistically significant interaction effect. The smiling expresser was perceived as more competent than the non‐smiling one, but only when participants were told he was from Romania, whereas this effect was not significant for the non‐immigrant expresser (see Figure 3).

Regarding emotions, we observed a significant main effect of smiling, with the smiling expression inciting more liking and less anger than the non‐smiling display. We also found a significant main effect of group membership, where the Romanian expresser provoked more admiration and liking than the ingroup expresser. Moreover, we detected significant interaction effects for *kama muta* and admiration. That is, the smiling Romanian expresser activated more feelings of *kama muta* and admiration than the non‐smiling Romanian. We did not find similar effects for the ingroup category.

Finally, host culture members were more willing to engage in interaction and less prone to avoid contact with the smiling (vs. non‐smiling) immigrant. Participants also reported more intentions to establish contact when the expresser was from Romania, but we did not detect significant interaction effects. We found no significant effects of neither smiling nor group membership on donation or volunteering intentions. However, it is worth mentioning that we detected a marginally significant result in the effect of smiling on volunteering intentions (Figure 3).

#### Indirect effects

Replicating the pattern observed in Studies 1–2, smiling increased approach intentions via perceived warmth (ingroup: *B* = 1.34, SE = 0.31, *p* < .001, 95% CI [0.74, 1.94]; outgroup: *B* = 1.65, SE = 0.40, *p* < .001, 95% CI [0.87, 2.44]) and felt liking (ingroup: *B* = 0.32, SE = 0.16, *p* = .047, 95% CI [0.004, 0.65]; outgroup: *B* = 0.39, SE = 0.18, *p* = .029, 95% CI [0.04, 0.74]). The effects were comparable in magnitude across ingroup and outgroup member conditions. Indirect effects on approach intentions via perceived competence or other emotions were not statistically significant.

Smiling also reduced avoidance intentions via perceived warmth for outgroup members only (*B* = −0.68, SE = 0.29, *p* = .018, 95% CI [−1.25, −0.12]). Indirect effects on avoidance intentions via perceived competence or emotions were not statistically significant.

Regarding donation intentions, a significant indirect effect of smiling emerged via perceived warmth for ingroup but not outgroup members (*B* = 0.80, SE = 0.39, *p* = .042, 95% CI [0.03, 1.56]). No similar significant effects were observed via competence or via emotions. We did not observe indirect effects of smiling on volunteering intentions via cognitive inferences or affective reactions.

Replicating the pattern from Studies 1–2, smiling enhanced approach intentions via warmth and liking for both ingroup and outgroup members. It further extended prior findings by showing reduced avoidance intentions via warmth (outgroup only) and increased donation intentions via warmth (ingroup only). However, indirect effects via competence, other emotions and on volunteering intentions were not significant. These effects should be interpreted with caution, given limited sample size (please see Table 8 for meta‐analytical integration of indirect effects). Path analyses testing indirect effects via cognitive inferences and affective reactions in Study 3 are presented in Tables S3.4–S3.7 in SOM, and also include models that test indirect effects of smiling on *kama muta* and admiration via warmth and competence (Figure S3.1).

## STUDY 4

### Participants

Participants were recruited via Netquest, a research company that manages online panels in Spain. Those who did not meet the inclusion criteria were automatically excluded (*N* = 192), as were those who failed the attention check (*N* = 669). In total, 1629 adults participated in the study (63.2% women, 36.6% men and 0.1% non‐binary or other; ages from 18 to 84 years; *M* = 47.17, SD = 13.13) all of whom were born in Spain to Spanish‐born parents. Among them, 68.9% of participants indicated being working actively, 13% pensioned, 10.3% unemployed and the sample leaned to the political left (from a scale with a mid‐point being 5, *M* = 4.28, SD = 2.55).

We determined the required sample size based on the assumption that the smallest effect size of interest for detecting both main and interaction effects in a 2 × 4 factorial ANOVA would be *f* = 0.10. This decision was informed by prior experimental research on the effects of emotional cues on helping intentions towards immigrants, which reported modest effect sizes (table I, Study 3, in the SOM of Bobowik et al., 2023). Power analyses using G*Power 3.1 (Faul et al., 2009) indicated that approximately 1096 participants would be needed to detect such an effect with 1−*β* prob. = .80 and *α* = 0.05. However, we also ensured that the sample size would allow testing indirect effects in a multiple mediation model within each cultural group. As no specific expected size for the indirect effect was available, we used a commonly observed effect in social psychology (*r* = 0.25; Lovakov & Agadullina, 2021) as the smallest effect of interest. Using the ShinyApp for mediation power estimation (Schoemann et al., 2017), we determined that for a model with three parallel mediators, a sample of 400 participants per group (total *N* = 1600) would be required to achieve 1−*β* prob. = .80 to detect the indirect effect at *α* = 0.05. Inclusion criteria were being at least 18 years old, born in Spain and having both parents also born in Spain.

### Procedure and materials

The experimental stimuli consisted of 20 facial images of men derived from the Chicago Face Database (CFD; Ma et al., 2015). These images were selected following a pre‐study validation conducted with a larger pool of 40 original CFD images (see details and results in the Data S1, Section IV). The purpose of the validation study was to assess whether the neutral‐expression faces were perceived by Spanish host culture participants as representative of one of four ethnocultural groups: Spanish, Moroccan, Ecuadorian or Romanian. Based on participants' ratings of group representativeness, we selected the five highest‐rated neutral faces for each group (totalling 20).^4^ While all neutral‐expression images are original photographs from the CFD, we generated corresponding smiling versions using an AI tool (openart.ai), ensuring that each smiling image was based on the same individual shown in the neutral condition. We used the following prompt: ‘This is the face of a person with a neutral expression, and I need the same photo but with a smile. The smile must look authentic and subtle (e.g. without showing teeth and without being overly pronounced), and you must keep everything else the same, including the background colours, clothing, photo size, person's proportions, skin and hair colours, as well as characteristic features, etc’.

We experimentally manipulated emotional expression and group membership. Specifically, participants were randomly shown an image of a man, varying by two factors: facial expression (neutral vs. happy) and ethnocultural/migrant background (Spain, Morocco, Ecuador, Romania) from a set of 4 pairs of neutral and happy faces, with the same description as in Study 3. The procedure was as in Study 3.

### Analytical strategy analyses

To examine the effects of the experimental manipulation and test our main hypotheses, we conducted 2 (expression) × 4 (group category) analyses of variance (ANOVAs) on each dependent variable: emotion expressed, group membership, perceived warmth and competence, *kama muta*, admiration, liking, anger, approach and avoidance intentions, as well as donation and volunteering intentions. We tested the main effects of both factors and the interaction effects between them. To test our mediation hypotheses, we conducted multiple mediation models with one predictor (emotion expression), two to four mediators and one outcome variable (either affiliative or helping intentions). We used bias‐corrected and accelerated (BCa) bootstrapping with 10,000 resamples to estimate confidence intervals for the indirect effects as in previous studies.

### Results

#### Descriptive findings and manipulation checks

Descriptive statistics and correlations among all variables are presented in the Tables S4.1–S4.3 in SOM.

##### Confirmatory analyses

Consistent with findings from Study 3, the immigrant men were perceived as less belonging to the participant's group compared to the men from the host culture (Table 6). Replicating Studies 1–3, the smiling expression elicited higher attributions of happiness and was perceived as less neutral than the non‐smiling expression. No significant interaction effects were detected.

**TABLE 6:** Means, standard deviations and ANOVA results: Impact of smiling depictions of immigrants (utgroup) versus non‐immigrants (ingroup) on host culture members‘ cognitive inferences, affective reactions and behavioural intentions (Study 4, N = 1629).

Variable	Group	Stimulus		Comparison			
		Non‐smiling display	Smiling display	Test^a^	*F*_(1,165)_	*p*	$\eta_{p}^{2}$ [90% CI]
		*M* (SD)	*M* (SD)				
Manipulation check							
Group Membership	Ingroup	2.40 (1.19)	2.29 (1.20)	GE	753.63	<.001	0.32 [0.29, 0.35]
				CE	1.81	.178	0.00 [0.00, 0.01]
	Outgroup	4.97 (1.77)	4.86 (1.79)	IE	0.00	.990	0.00 [0.00, 1.00]
			
Neutral	Ingroup	4.97 (1.40)	3.35 (1.40)	GE	3.03	.082	0.00 [0.00, 0.01]
				CE	648.00	<.001	0.29 [0.26, 0.31]
	Outgroup	4.91 (1.37)	3.13 (1.38)	IE	1.04	.307	0.00 [0.00, 0.00]
			
Happy	Ingroup	3.04 (1.35)	5.09 (0.99)	GE	1.96	.162	0.00 [0.00, 0.01]
				CE	1179.07	<.001	0.42 [0.39, 0.45]
	Outgroup	2.95 (1.28)	4.99 (1.12)	IE	0.03	.873	0.00 [0.00, 0.00]
			
Cognitive inferences							
Warmth	Ingroup	3.86 (1.17)	5.03 (1.01)	GE	0.46	.496	0.00 [0.00, 0.00]
				CE	329.47	<.001	0.17 [0.14, 0.20]
	Outgroup	4.02 (1.22)	4.96 (1.02)	IE	3.29	.070	0.00 [0.00, 0.01]
			
Competence	Ingroup	4.25 (0.97)	4.75 (1.03)	GE	0.11	.741	0.00 [0.00, 0.00]
				CE	45.57	<.001	0.03 [0.02, 0.04]
	Outgroup	4.34 (1.07)	4.63 (0.99)	IE	3.46	.063	0.00 [0.00, 0.01]
			
Affective reactions							
Liking	Ingroup	2.94 (1.39)	3.84 (1.60)	GE	0.57	.452	0.00 [0.00, 0.00]
				CE	107.21	<.001	0.06 [0.04, 0.08]
	Outgroup	3.10 (1.46)	3.81 (1.47)	IE	1.22	.269	0.00 [0.00, 0.00]
			
Anger	Ingroup	1.97 (1.24)	1.46 (0.89)	GE	1.44	.231	0.00 [0.00, 0.00]
				CE	37.23	<.001	0.02 [0.01, 0.04]
	Outgroup	1.94 (1.28)	1.64 (1.11)	IE	2.45	.118	0.00 [0.01, 0.01]
			
*Kama muta*	Ingroup	2.55 (1.35)	2.99 (1.50)	GE	3.20	.074	0.00 [0.00, 0.01]
				CE	39.29	<.001	0.02 [0.01, 0.04]
	Outgroup	2.69 (1.39)	3.13 (1.41)	IE	0.00	.982	0.00 [0.00, 1.00]
			
Admiration	Ingroup	2.40 (1.34)	2.92 (1.53)	GE	3.64	.057	0.00 [0.00, 0.01]
				CE	52.08	<.001	0.03 [0.02, 0.05]
	Outgroup	2.56 (1.38)	3.06 (1.43)	IE	0.01	.932	0.00 [0.00, 0.00]
			
Behavioural intentions							
Approach	Ingroup	3.19 (1.49)	3.82 (1.53)	GE	2.73	.099	0.00 [0.00, 0.01]
				CE	40.93	<.001	0.02 [0.01, 0.04]
	Outgroup	3.43 (1.58)	3.86 (1.47)	IE	1.49	.223	0.00 [0.00, 0.01]
			
Avoidance	Ingroup	2.71 (1.44)	2.35 (1.29)	GE	2.33	.127	0.00 [0.00, 0.01]
				CE	21.53	<.001	0.01 [0.01, 0.02]
	Outgroup	2.56 (1.53)	2.24 (1.39)	IE	0.05	.827	0.00 [0.00, 0.00]
			
Donation	Ingroup	2.62 (1.39)	2.57 (1.50)	GE	0.01	.906	0.00 [0.00, 0.00]
				CE	0.08	.782	0.00 [0.00, 0.00]
	Outgroup	2.61 (1.58)	2.60 (1.47)	IE	0.05	.832	0.00 [0.00, 0.00]
			
Volunteering	Ingroup	3.72 (1.65)	3.54 (1.61)	GE	0.04	.845	0.00 [0.00, 0.00]
				CE	0.01	.929	0.00 [0.00, 0.00]
	Outgroup	3.57 (1.75)	3.64 (1.68)	IE	1.68	.195	0.00 [0.00, 0.01]
			

*Note*: Approach = Approach intentions; Avoidance = Avoidance intentions; Donation = Donation intentions; Volunteering = Volunteering intentions; Ingroup = the Spanish man; Outgroup = the Romanian man. *M* (SD) represent means and standard deviations, respectively. $\eta_{p}^{2}$ [90% CI] represent eta squared and the lower‐ and upper‐limit of its confidence interval.

^a^ The ANOVA contrasts: GE = Group membership effects; CE = Condition effects (smiling vs. non‐smiling display); and IE = Interaction effects (Group membership * Condition). Number of participants by condition: Neutral‐Ingroup: *n* = 207; Neutral‐Outgroup: *n* = 595; Smile‐Ingroup: *n* = 212; Smile‐Outgroup: *n* = 615.

##### Exploratory analyses

We also performed similar manipulation checks in a 2 × 4 ANOVA, using four group categories as a second factor. These results are reported in Table S4.4 in SOM, confirming the successful manipulation of happiness expression and group membership.

#### Main and interaction effects

##### Confirmatory analyses

**FIGURE 4:**
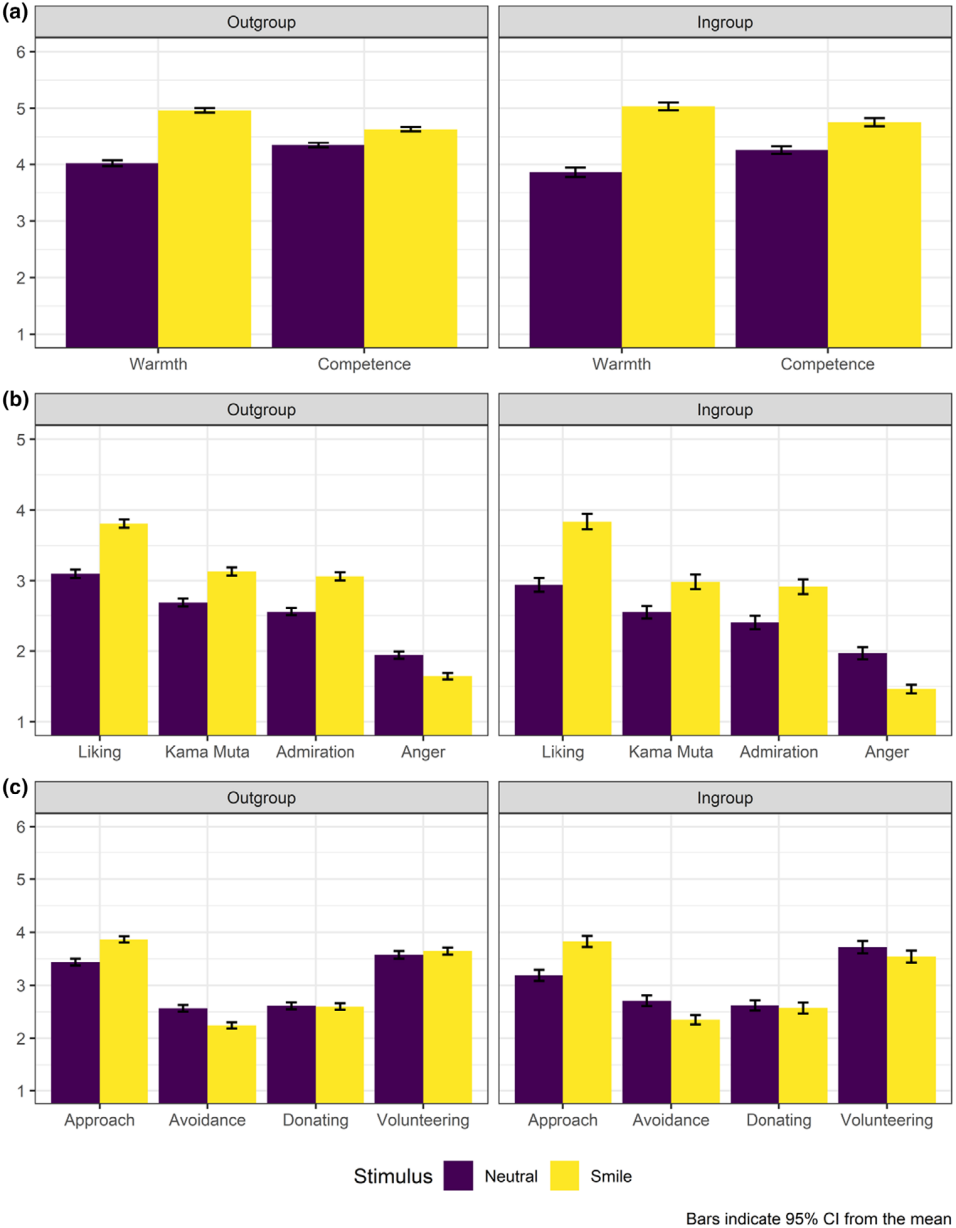
Means, standard deviations and ANOVA results: Impact of smiling depictions of immigrants (outgroup) versus non‐immigrants (ingroup) on host culture members' cognitive inferences, affective reactions and behavioural intentions (Study 4, *N* = 1629).

A two‐way ANOVA revealed significant main effects of emotion expression (smiling vs. neutral) on inferences of warmth and competence. Participants rated the smiling men as both warmer and more competent than the non‐smiling men. There were no statistically significant main effects of group category, nor any interaction effects, indicating that the impact of the smiling expression was comparable for immigrant and host culture men (Figure 4). Regarding emotions, we observed a significant main effect of smiling, with the smiling expression inciting more liking, admiration and *kama muta*, as well as less anger than the non‐smiling display. We did not observe any significant interaction effects. Finally, participants reported greater willingness to engage in interaction and lower avoidance tendencies towards smiling men compared to non‐smiling men. However, smiling did not significantly affect helping intentions. No significant interaction effects were observed for behavioural intentions.

##### Exploratory analyses

We also performed similar analyses in a 2 × 4 ANOVA, using four group categories as a second factor. These results have replicated the effects of smiling on all dependent variables (Table S4.4). We also observed the independent effect of group membership on anger, *kama muta*, admiration, as well as approach and avoidance intentions. We did not detect any significant interaction effects, disconfirming the results regarding *kama muta* and admiration found in Study 3.

#### Indirect effects

Study 4 offered an opportunity to test the indirect effects with a more robust sample size. We found statistically significant indirect effects of smiling on approach intentions via perceived warmth (ingroup: *B* = 0.59, SE = 0.11, *p* < .001, 95% CI [0.38, 0.80]; outgroup: *B* = 0.58, SE = 0.06, *p* < .001, 95% CI [0.46, 0.70]) and competence (ingroup: *B* = 0.22, SE = 0.06, *p* < .001, 95% CI [0.10, 0.34]; outgroup: *B* = 0.09, SE = 0.02, *p* < .001, 95% CI [0.04, 0.13]). Similar effects were obtained via felt liking (ingroup: *B* = 0.46, SE = 0.09, *p* < .001, 95% CI [0.28, 0.63]; outgroup: *B* = 0.40, SE = 0.05, *p* < .001, 95% CI [0.29, 0.50]), admiration (ingroup: *B* = 0.14, SE = 0.06, *p* = .017, 95% CI [0.03, 0.25]; outgroup: *B* = 0.08, SE = 0.03, *p* = .003, 95% CI [0.03, 0.13]) and reduced anger (ingroup: *B* = 0.06, SE = 0.03, *p* = .037, 95% CI [0.004, 0.12]; outgroup: *B* = 0.06, SE = 0.02, *p* < .001, 95% CI [0.03, 0.08]). All effects were relatively small and similar in strength for both ingroup and outgroup members.

We also found significant indirect effects of smiling on avoidance intentions via warmth (ingroup: *B* = −0.64, SE = 0.12, *p* < .001, 95% CI [−0.86, −0.41]; outgroup: *B* = −0.54, SE = 0.06, *p* < .001, 95% CI [−0.67, −0.42]) and competence (ingroup only: *B* = 0.11, SE = 0.05, *p* = .029, 95% CI [0.01, 0.21]). Similar effects were found via liking (ingroup: *B* = −0.25, SE = 0.07, *p* < .001, 95% CI [−0.39, −0.11]; outgroup: *B* = −0.27, SE = 0.04, *p* < .001, 95% CI [−0.35, −0.19]) and reduced anger (ingroup: *B* = −0.24, SE = 0.06, *p* < .001, 95% CI [−0.36, −0.13]; outgroup: *B* = −0.16, SE = 0.04, *p* < .001, 95% CI [−0.24, −0.09]).

Regarding donation intentions, there were significant indirect effects of smiling for outgroup members via perceived warmth (*B* = 0.23, SE = 0.06, *p* < .001, 95% CI [0.12, 0.34]), perceived competence (*B* = 0.10, SE = 0.03, *p* < .001, 95% CI [0.04, 0.15]) and felt admiration (*B* = 0.08, SE = 0.03, *p* = .009, 95% CI [0.02, 0.15]). For ingroup members, there was an indirect effect via reduced anger (*B* = −0.09, SE = 0.04, *p* = .014, 95% CI [−0.17, −0.02]), although this effect unexpectedly inhibited donation intentions. We additionally observed that smiling had similar indirect effects on donation intentions via felt liking across both groups (ingroup: *B* = 0.20, SE = 0.07, *p* = .006, 95% CI [0.06, 034]; outgroup: *B* = 0.25, SE = 0.04, *p* < .001, 95% CI [0.17, 0.33]), replicating previous studies.

Regarding volunteering intentions, there were significant indirect effects of smiling for outgroup members via perceived competence (*B* = 0.11, SE = 0.03, *p* < .001, 95% CI [0.05, 0.17]) and felt admiration (*B* = 0.08, SE = 0.03, *p* = .022, 95% CI [0.01, 0.14]). We also found that perceived warmth (ingroup: *B* = 0.48, SE = 0.13, *p* < .001, 95% CI [0.23, 0.73]; outgroup: *B* = 0.42, SE = 0.07, *p* < .001, 95% CI [0.29, 0.54]) and felt liking (ingroup: *B* = 0.35, SE = 0.09, *p* < .001, 95% CI [0.17, 0.52], outgroup: *B* = 0.35, SE = 0.05, *p* < .001, 95% CI [0.25, 0.46]) had indirect effects on increasing volunteering intentions across both group categories.

Using a larger and more powered sample, Study 4 replicated the indirect effects of smiling via warmth and liking observed in Studies 1–3 for both ingroup and outgroup members, and extended them to additional mediators—competence, admiration and reduced anger, which showed comparable effects across groups. For both groups, smiling increased helping intentions through felt liking and perceived warmth, except for perceived warmth in donation intentions for ingroup members. For outgroup members only, smiling also enhanced helping intentions indirectly through higher perceived competence and felt admiration, in addition to warmth. In contrast, for ingroup members only, smiling had an indirect effect via reduced anger, which unexpectedly led to lower donation intentions. Path analyses testing indirect effects via cognitive inferences and affective reactions in Study 4 are presented in Tables S4.5–S4.8 in SOM. Please see meta‐analytical integration of indirect effects in Table 8.

## META‐ANALYTICAL INTEGRATION

To obtain more robust integrative effects, we conducted a meta‐analytical integration of all studies, following the criteria of Cumming (2013) and Del Re (2015).

### Pooled main effects

**FIGURE 5:**
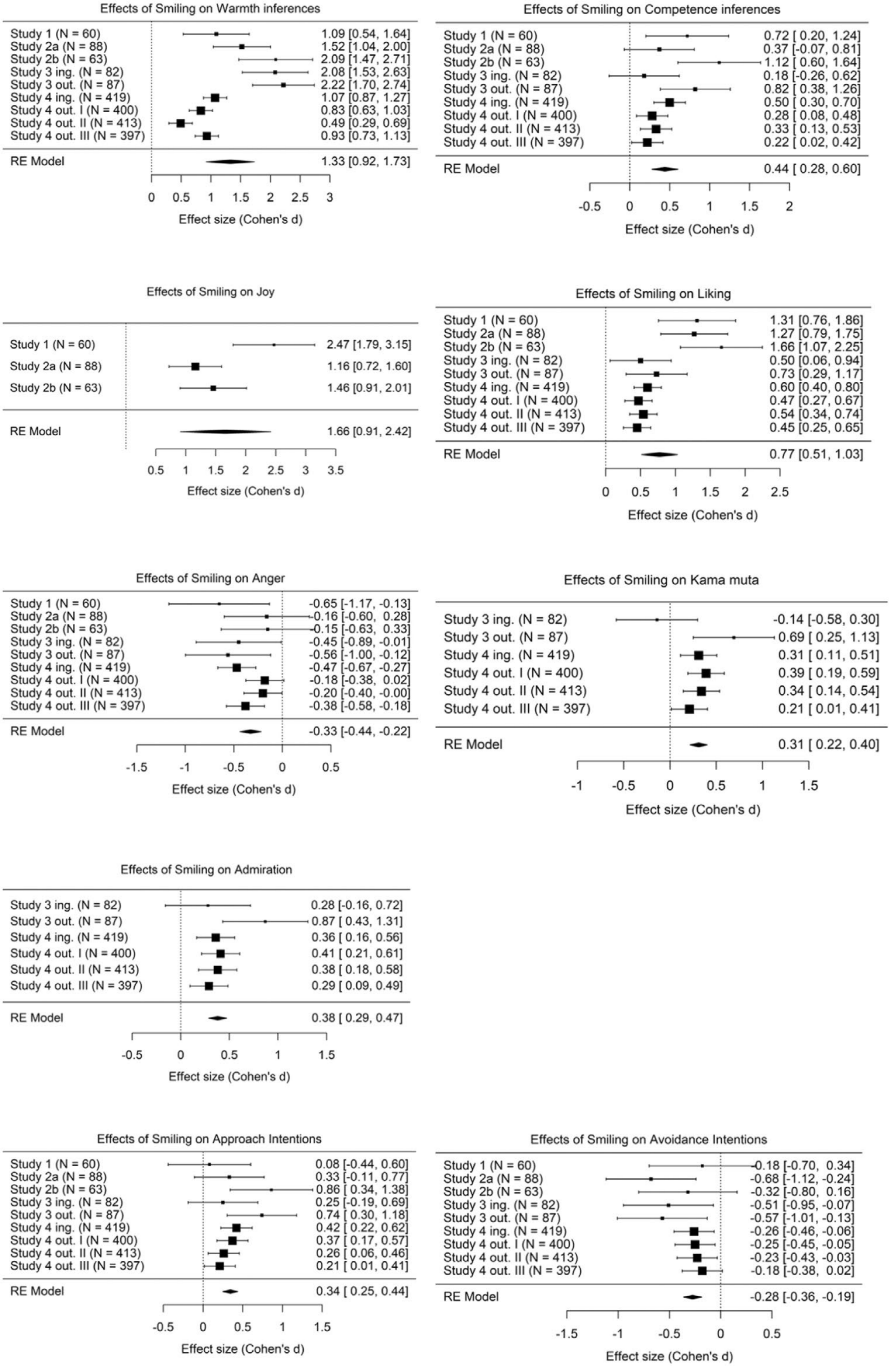
Forest plots of the effects of smiling depictions on host culture members’ cognitive inferences, affective reactions, and behavioural intentions across studies 1–4. Note. The expressers per each sample are: Study 1: Senegalese man; Study 2a: Moroccan man; Study 2b: Pakistani man; Study 3: Spanish (ingroup) vs. Romanian man (outgroup); Study 4: Spanish (ingroup) vs. Moroccan (outgroup I) vs. Ecuadorian (outgroup II) vs. Romanian (outgroup III).

The analyses showed an overall significant effect of an exposure to smiling (versus neutral) depictions of an immigrant on the observer's inferences of warmth and competence, self‐reported joy, liking, admiration, *kama muta* and anger, as well as approach and avoidance intentions (Table 7, Figure 5). We did not integrate the effects for donation and volunteering intentions given the lack of significant main effects of smiling in both Study 3 and 4.

**TABLE 7:** Meta‐analytical integration of the effects of smiling depictions on host culture members' cognitive inferences, affective reactions and behavioural intentions across studies 1–4.

Variable	Random Model		Heterogeneity Tests			
	*d*_*pooled*_ [95% CI]	*p*	Q	df	*p*	*I* ^2^
Warmth	1.32 [0.92, 1.73]	<.001	*Q =* 81.64	8	<.001	94.22%
Competence	0.44 [0.28, 0.60]	<.001	*Q =* 19.40	8	.013	62.99%
Joy	1.66 [0.91, 2.42]	<.001	*Q =* 10.16	2	.006	82.21%
Liking	0.77 [0.51, 1.03]	<.001	*Q =* 31.10	8	<.001	85.77%
Anger	−0.33 [−0.44, −0.22]	<.001	*Q =* 10.05	8	.261	25.85%
Kama muta	0.31 [0.22, 0.40]	<.001	*Q*_=_ 8.66	5	.123	0.04%
Admiration	0.38 [0.29, 0.47]	<.001	*Q =* 5.94	5	.312	0.004%
Approach	0.34 [0.25, 0.44]	<.001	*Q =* 11.25	8	.188	7.59%
Avoidance	−0.28 [−0.36, −0.19]	<.001	*Q =* 7.48	8	.486	0.00%

*Note:* Approach = Approach intentions; Avoidance = Avoidance intentions. *d*_*pooled*_ and [95% CI] represent the pooled Cohen's *d* and the 95% confident interval, respectively. *Q*(*df*) represent the *Q* statistic of heterogeneity and its degrees of freedom. *I* ^2^ and τ^2^ represent the percentage of variation across studies due to heterogeneity and the true variance in terms of the scale of the effect size (Cohen's *d*). For a visual representation of these tests, see SOM. * and ** indicate *p* values of <.05 and <.01, respectively.

Heterogeneity tests (i.e. *Q* and *I* ^2^) showed that the effects of smiling on felt anger, *kama muta*, admiration and approach and avoidance intentions were homogeneous. In contrast, the results for warmth, joy and liking were heterogeneous, indicating that additional factors may account for the variability observed (e.g. cultural meanings of smiling, sample characteristics).

### Pooled indirect effects

A meta‐analytical integration of the indirect effects showed that, across studies, smiling increased approach intentions and reduced avoidance intentions mainly via perceived warmth and felt liking, which consistently emerged as the strongest mediators. Additionally, smaller but reliable effects were observed through perceived competence, admiration and kama muta. However, the indirect effect via kama muta seems to disappear once controlled for admiration, as shown in multivariate analyses reported in each independent study. Reduced anger further contributed to lowering avoidance intentions, whereas joy—though associated with greater approach—appeared less central in multivariate analyses reported in each of the studies once liking was accounted for (Table 8).^5^

**TABLE 8:** Meta‐analytical integration of the indirect effects of smiling depictions on host culture members' cognitive inferences, affective reactions and behavioural intentions across studies 1–4.

Effect	Random Model		Heterogeneity Tests			
	Estimate [95% CI]	*p*	Q	df	*p*	*I* ^2^
Pooled Indirect Effects of Smiling on Approach Intentions via						
Warmth	0.32 [0.25, 0.39]	<.001	31.6	8	<.001	82.30%
Competence	0.09 [0.07, 0.12]	<.001	11.27	8	.190	12.16%
Joy	0.32 [0.23, 0.41]	<.001	0.81	2	.670	0.00%
Liking	0.23 [0.16, 0.29]	<.001	25.8	8	<.001	77.86%
Anger	0.01 [−0.00, 0.01]	.142	4.64	8	.800	0.00%
Kama muta	0.07 [0.03, 0.11]	<.001	11.36	5	.040	56.85%
Admiration	0.11 [0.08, 0.13]	<.001	2.09	5	.840	0.00%
Pooled Indirect Effects of Smiling on Avoidance Intentions via						
Warmth	−0.2 [−0.27, −0.13]	<.001	26.59	8	<.001	83.49%
Competence	−0.04 [−0.05, −0.03]	<.001	14.07	8	.080	0.00%
Joy	−0.06 [−0.15, 0.04]	0.228	0.74	2	.690	0.00%
Liking	−0.07 [−0.09, −0.06]	<.001	9.54	8	.300	0.32%
Anger	−0.05 [−0.07, −0.03]	<.001	10.99	8	.200	26.63%
Kama muta	−0.01 [−0.02, −0.00]	.015	7	5	.220	22.56%
Admiration	−0.02 [−0.03, −0.01]	.001	4.8	5	.440	21.27%

*Note:* *d*_*pooled*_ and [95% CI] represent the pooled Cohen's *d* and the 95% confident interval, respectively. *Q*(*df*) represent the *Q* statistic of heterogeneity and its degrees of freedom. *I* ^2^ represents the percentage of variation across studies due to heterogeneity.

## GENERAL DISCUSSION

Literature underscores that immigrants are frequently depicted through depersonalized collective imagery, lacking individual facial features (Azevedo et al., 2021; Bleiker et al., 2013). Such portrayals contribute to dehumanization, potentially leading to ‘compassion fatigue’ (Kędra & Sommier, 2018). Our investigation, encompassing four experiments across five samples, examined the potential of positive emotional expressions—namely, smiling—as a counter‐strategy to these biases. Our findings reveal that the depictions of smiling immigrant men significantly shifted the perceptions of host culture members, enhancing viewing of these individuals as embodying greater warmth and competence. Moreover, these positive portrayals evoked stronger feelings of liking, joy, admiration and *kama muta* (i.e. feeling moved), while reducing anger. Our research also highlights cognitive and affective pathways through which positive emotional expressions can enhance prosocial intentions and mitigate avoidance intentions towards immigrants.

### Cognitive inferences

Studies illustrate that depersonalized, massified portrayals of immigrants lacking distinguishable facial features tend to diminish perceptions of warmth (Azevedo et al., 2021). Conversely, more nuanced, emotionally resonant depictions highlighting facial expressions can enhance perceived warmth (Bobowik et al., 2023). Our findings reveal that displaying a smile in images of immigrants also serves as a powerful counter‐narrative to negative stereotypes, effectively elevating the perceived warmth of an immigrant individual. This aligns with evidence that smiling individuals are seen as warmer, more sociable and morally upright (Belkin & Rothman, 2017; Hess et al., 2002; Senft et al., 2016). Crucially, our study extends this evidence to groups frequently portrayed in media as threatening and ‘other’ (Martikainen & Sakki, 2021).

Our findings further clarify the complex role of smiling depictions in shaping perceptions of immigrants: such portrayals enhance perceptions of both warmth *and* competence, supporting the idea that smiling communicates agency and capability (Belkin & Rothman, 2017; Hess et al., 2002; Krys et al., 2016), possibly reflecting a more global evaluative process rather than dimension‐specific changes. Such depictions seem to challenge the stereotypical view of migrants as passive or merely conforming to host culture expectations—a narrative that can inadvertently foster social distance (Martikainen & Sakki, 2021). Instead, they express immigrants' openness and proactive agency in navigating the complexities of their new environments. However, it is also important to note that both ingroup and outgroup members in Studies 3 and 4 were evaluated similarly in warmth and competence already in the control condition, disconfirming the idea of ingroup favouritism in evaluations on these dimensions as proposed by the Stereotype Content Model (Fiske et al., 2002).

### Affective reactions

Our research contributes to a better understanding of how positive nonverbal cues—particularly smiles from immigrants—shape the emotional responses of the host culture members. We found that portrayals of smiling immigrant men engendered feelings of joy (Studies 1–2) and liking (Studies 1–4). These results support broader literature suggesting that expressions of happiness can influence observers' emotional states (e.g. Hess & Fischer, 2013). Our findings also extend evidence that smiles enhance perceptions of trustworthiness and likability (e.g. Belkin & Rothman, 2017; Oosterhof & Todorov, 2009; Scharlemann et al., 2001).

Our research also delves into self‐transcendent emotions like admiration and *kama muta*, which were evoked by smiles from immigrants. Recent studies show that emotive portrayals and positively framed visual narratives of immigrants can activate such other‐oriented emotions, ranging from *kama muta* (or compassion) to feelings of admiration and gratitude (Alonso‐Arbiol et al., 2024; Bobowik et al., 2023). Moreover, our data indicated reduced anger towards smiling immigrant figures compared to neutral expressions. This finding prompts a reflection on whether the decreased anger targets immigrants or the morally contentious circumstances they face, highlighting the need for future research into the nuances of moral outrage in response to immigrants' experiences.

### Smiling and behavioural intentions

Our research highlights the impact of expressing positive emotions by immigrants on social interaction intentions. Integrative analyses revealed a consistent effect of exposure to images of a smiling immigrant (versus neutral expression) on increasing host culture members' approach intentions and decreasing avoidance intentions, corroborating earlier findings (e.g. Bernstein et al., 2010; Scharlemann et al., 2001). However, we did not find significant effects of smiling on intentions to offer instrumental help, such as donations or volunteering, contrary to some previous evidence (e.g. Guéguen & De Gail, 2003). However, we did observe some indirect effects on helping intentions, suggesting that the impact of emotional cues on more costly actions is more limited and indirect (these findings are discussed in detail in the following section). In addition, possibly, smiling may motivate direct engagement, such as getting to know immigrants, rather than assistance like monetary donations or volunteering, which would be more expected from someone who is in need. Future research should further clarify how expressing positive emotions influences helping disadvantaged individuals.

### The role of group membership

In Studies 3 and 4, we examined whether group membership moderates the effects of smiling expressions on observers' socio‐cognitive and emotional responses. Across both studies—including a robust, large‐scale online experiment using multiple stimuli and three distinct outgroup categories—we found no consistent evidence that group membership influenced the strength of the effects on key cognitive inferences and emotional responses, such as perceived warmth, felt liking and anger or behavioural intentions like willingness to engage socially or help. Smiling had comparable effects on these dimensions across ingroup and outgroup members, aligning with Paulus and Wentura's (2018) findings that group membership and emotion have independent effects. Contrary to our hypotheses, this challenges prior evidence suggesting that group membership may amplify (Cooley et al., 2018; Hareli et al., 2009; Senft et al., 2016; Ybar & Hess, 2007) or dampen (Gurbuz et al., 2024; Paulus & Wentura, 2014) the impact of emotional expressions in intergroup contexts, suggesting instead that smiles serve as a broadly universal signal of affiliative intent.

In contrast, perceptions of competence appeared more sensitive to group dynamics, aligning with previous research highlighting the role of culture in how smiles are interpreted with respect to competence (Krys et al., 2014, 2016). While Study 3 showed a stronger effect of smiling on perceived competence for outgroup members, Study 4 showed the opposite trend, though the moderation did not reach statistical significance. One possible explanation for this inconsistency lies in the culture‐specific stimuli used. In Study 4, both the Spanish ingroup and the Romanian outgroup members were represented by individuals with relatively ‘White’ phenotypic features, which may have attenuated perceived group distinctions. Notably, the Romanian men in Study 4 were rated positively even in the neutral (non‐smiling) condition—suggesting that the appearance or demeanour of these specific targets may have prompted more favourable baseline evaluations. This means that the Romanian outgroup was not ‘racialized’ by using faces of different ethnocultural origin, because we only changed the textual information about the origin of the target. We believe this might explain the more positive evaluation of the Romanian expresser in Study 4 compared to other outgroup expressers. These results point to the importance of not treating group membership as a fixed or monolithic category; visual cues, racialized phenotypes and associated stereotypes likely interact with nationality and migrant status in complex ways.

We also observed inconsistent effects of smiling on prosocial, self‐transcendent emotions, specifically *kama muta* and admiration. In Study 3, smiles from the Romanian outgroup member significantly elevated these emotions, whereas no such effects emerged for ingroup targets. This finding highlights that self‐transcendent emotional responses may be more readily triggered by expressions from individuals who are perceived as culturally distant or socially marginalized. Smiles from immigrants may be read not only as affiliative but also as morally moving, perhaps reflecting admiration for their resilience or courage. This supports existing literature on the specific social function of self‐transcendent emotions in fostering openness and solidarity across group boundaries (e.g. Stellar et al., 2017). Interestingly, such responses were not observed in Study 4, suggesting that the elicitation of *kama muta* and admiration may depend on both the social identity and phenotypic cues of the expresser, as well as on subtle contextual factors. Further systematic research is required to clarify the interplay between happiness expressions and group membership in eliciting self‐transcendent emotions.

Even though group membership did not consistently moderate effects—aside from the self‐transcendent emotions in Study 3—the meta‐analytical integration of results revealed notable heterogeneity across studies and ethnocultural groups. This heterogeneity was evident in the differential size of effects across several outcomes, including warmth, competence, joy and liking. For instance, the effect of smiling on perceived warmth varied across migrant groups: it was relatively pronounced for non‐immigrant men and for Pakistani and Romanian immigrants, but substantially weaker for Senegalese, Moroccan and Ecuadorian men. Particularly in the case of the Senegalese man, the lower relative gain from smiling may reflect a ceiling effect, as participants attributed high warmth to him even in the neutral condition. This could be due to methodological differences across studies (e.g. types of photos used) or differences in sample composition.

Further, the emotional impact of smiling also varied depending on the expresser's ethnocultural background. Smiles from the Senegalese man elicited nearly twice as much joy as those from other individuals. This pronounced effect may reflect broader societal narratives—for example, Spanish media portrayals of Sub‐Saharan immigrants frequently highlighting hardship and suffering. Against this backdrop, a joyful expression from a Senegalese man may appear especially striking, producing a powerful emotional contrast. This suggests that societal and media contexts influence not only intergroup attitudes but also the affective meaning attached to basic emotional expressions.

We also observed variability in responses related to liking, which may stem in part from differences in how affective terms were operationalized across studies—for example, using ‘liking’ in one case and ‘affectionate’ in another. These linguistic and conceptual differences likely influenced the emotional granularity of participants' responses and highlight the importance of consistency and precision in measurement design, especially when comparing across cultural groups.

These findings illustrate the complex interplay between universal social signals (like smiling) and the culturally and phenotypically situated contexts in which such expressions are perceived. While smiles generally foster affiliative responses across group lines, their effects on competence and self‐transcendent emotions appear more context‐dependent—shaped by phenotype, national identity, stereotypes and the emotional connotations of migration itself. The observed heterogeneity across studies and stimuli underscores the need for more systematic, intersectional and reflexive research on how group membership, cultural narratives and visual cues jointly influence emotional and social perception. Future work should attend more closely to the situated nature of intergroup encounters, the intersectionality of migrant identities and the role of broader societal discourses in shaping even seemingly universal forms of human expression.

### Explanatory mechanisms

Consistent across four studies, the effects of smiling on social intentions operated primarily through indirect pathways involving perceived warmth and felt liking, in line with predictions from the EASI model (van Kleef, 2009), which posits that both cognitive inferences and affective reactions to emotional expressions shape observers' behavioural responses.

Specifically, smiling reliably increased approach intentions through heightened perceptions of warmth (*d* = 0.32) and feelings of liking (*d* = 0.26), a pattern replicated across all studies and for both ingroup and outgroup members. These findings align with the BIAS Map model (Cuddy et al., 2007), which highlights warmth as a fundamental dimension of social perception that guides behavioural tendencies towards cooperation or avoidance. Indeed, research shows that perceiving migrants as warm predicts helping and cooperative intentions (e.g. Becker et al., 2019) and that warmth is a key mechanism in shaping reactions to emotional portrayals of immigrants (Bobowik et al., 2023).

Our results also suggest that positive emotions such as liking expand behavioural openness and affiliative tendencies—consistent with the broaden‐and‐build theory of positive emotions (Fredrickson, 2013) and related work showing that joy and liking can reduce intergroup bias (Johnson & Fredrickson, 2005; Pittinsky & Montoya, 2016). Although emotional contagion of joy initially appeared to be a relevant mechanism, its effect diminished once positive attitudes such as liking were accounted for—suggesting that its apparent influence may be overshadowed by other‐focused emotions like liking, which are more directly linked to interpersonal relations and prosocial behaviour.

Notably, Study 4 also provided evidence that approach intentions towards smilers are also motivated by stronger perceptions of competence (*d* = 0.10) and hence feelings of admiration (*d* = 0.11), which was supported by a meta‐analytical integration of all studies.; Although smaller in magnitude than warmth and liking, these effects indicate that smiling can also elicit respect or esteem and further reinforce prosocial responses. Self‐transcendent emotions such as admiration and *kama muta* are theorized to promote bonding, caregiving and cooperation (Stellar et al., 2017), with admiration, in particular, linked to affiliative intergroup behaviour towards diverse groups (Alonso‐Arbiol et al., 2024; Bobowik et al., 2023; Lizarazo‐Pereira et al., 2022; Oliver et al. (2015); Seger et al. (2017). Kama muta (feeling moved) occasionally mediated effects on approach intentions but did not remain significant once admiration was controlled, suggesting conceptual overlap between both emotions. These effects were generally comparable across ingroup and outgroup targets, although admiration and competence tended to play a somewhat stronger role for outgroup members, suggesting that smiling may motivate perceptions of outgroup agency and elicit respect especially across group boundaries.

Avoidance intentions were in turn reduced by smiling, particularly via attributions of warmth (*d* = −0.19) and feelings of liking (*d* = −0.10) —though these effects were weaker compared to approach intentions and somewhat less consistent across studies. Notably, the data, particularly from Study 4, provided evidence that smiling could reduce avoidance intentions through reduced hostility. This aligns with the notion that smiling can help mitigate negative emotional reactions, particularly anger, by eliciting emotional mimicry and fostering more positive affective states in observers (Hess & Fischer, 2013). In intergroup contexts, a smile may also counteract stereotypes of low warmth and high competitiveness often associated with immigrants (Fiske et al., 2002), thereby lowering anger and perceived threat. Such mechanisms could explain why reductions in avoidance emerged primarily when hostility was diminished, as observed in Study 4.

Across studies, smiling enhanced helping intentions—including both donation and volunteering—but only indirectly, through cognitive and affective mediators rather than direct effects. These pathways were therefore weaker and less immediate than those observed for approach and avoidance intentions. Felt liking emerged as a consistent mediator for both types of helping and regardless of expresser's group membership, underscoring the affiliative function of smiling as a cue that fosters positive affect and prosociality (van Kleef & Côté, 2022).

In Study 4, outgroup smiles in particular produced broader indirect effects on helping intentions via warmth, competence and admiration, suggesting that smiling served as a counter‐stereotypical cue that disconfirmed perceptions of threat or coldness (Bijlstra et al., 2010; Crisp & Nicel, 2004). These mechanisms are consistent with the BIAS Map (Cuddy et al., 2007), linking warmth and competence to facilitative behaviours, while admiration reflects self‐transcendent emotions that expand affiliative motivation towards stigmatized groups (Alonso‐Arbiol et al., 2024; Lizarazo‐Pereira et al., 2022).

For ingroup members, smiling indirectly increased donation intentions via warmth (Study 3) and reduced anger (Study 4), though the latter unexpectedly inhibited donation—possibly because reduced arousal can attenuate moral motivation.

Jointly, our work partially clarifies the role of cognitive judgements and affective responses in how smiling by immigrants influences behavioural intentions towards them, supporting the EASI model (van Kleef, 2009; van Kleef & Côté, 2022) and BIAS Map model (Cuddy et al., 2007).

### Limitations and future research

The findings and implications of this research should be interpreted with its constraints in mind. Our research focused on smiling immigrant men, so conclusions are limited to this group, and future research could explore systematically the role of the expresser's and perceiver's gender. Unfortunately, these analyses were beyond the scope of the current paper due to the lack of a sufficient number of male participants in Studies 1–3, as well as an exclusive focus on men as expressers.

Another limitation is that our samples included a predominance of women and left‐leaning participants—groups less likely to exhibit ingroup favouritism and, in some cases, more inclined towards outgroup favouritism, although a left‐leaning tendency reflects the political preferences of the current Spanish society. It is also possible that women evaluated ingroup male targets less positively, with gender becoming a more salient category than immigrant status in these judgements. Although preliminary analyses suggest that gender and political orientation may influence responses, these effects are beyond the scope of the present paper and warrant examination in future research.

Although our predictor was manipulated experimentally, the mediators and outcomes were correlational. Future research should experimentally manipulate these mediators to clarify the direction of influence. Nevertheless, our statistical analyses were informed by established theoretical frameworks describing how affective and cognitive mechanisms connect smiling with behavioural intentions (Fiedler et al., 2018).

Other contextual cues, beyond ethnicity or immigrant status, might also affect how smiling is interpreted. Our study's focus on a smiling immigrant man does not address why the smile is expressed or its sincerity. Future studies should explore how social context affects the perception of smiles and whether they may backfire if perceived as insincere. For example, smiles might have different implications in professional versus personal contexts.

We acknowledge that our experimental design involves an overlap between immigrant status and ethnicity and that the intersectionality of these categories warrants further exploration in future research. However, this overlap reflects the social reality in many contexts—particularly in Western Europe (e.g. Spain) and North America (e.g. the U.S.)—where immigrant populations are disproportionately racialized. Attempting to fully disentangle immigrant status from racialized appearance risks creating an artificial experimental context with limited ecological validity. In real‐world interactions, these categories are frequently conflated in public discourse, perception and policy. While it remains important to conceptually distinguish ethnicity from migration background, our study is designed to capture how immigrant representations are encountered and interpreted in everyday social perception.

While this investigation focused on positive emotions, future research should explore the effects of negatively framed images of immigrants on support for minority rights and mobilization. Immigrants may be portrayed as either victims or as emancipated citizens, and both types of frames may impact public opinion. Future research could tap other behavioural responses, such as hiring or befriending immigrants, and the impact of smiling on actions like sharing, donating or volunteering, shifting the focus away from intergroup harmony to other dimensions, such as supporting policies and collective actions that address the needs of socially disadvantaged groups.

### Practical implications and a critical perspective

Our work yields several practical implications that are both insightful and necessary. The findings suggest that immigrants' displays of positive emotions in awareness‐raising social campaigns and general mass media could serve as a counter‐strategy to prevalent negative stereotypes. By humanizing migrated individuals, these positive depictions can directly influence how members of the host society perceive and respond to newcomers. Specifically, these images can foster perceptions of not only warmth but also competence, evoke emotions of admiration and reduce feelings of anger towards immigrants. Such strategies can potentially enhance the effectiveness of anti‐racist campaigns that utilize visual depictions of migration (e.g. Crisp & Nicel, 2004; Dasgupta & Greenwald, 2001). For instance, future campaigns should ensure the presence of positive emotional expressions, ideally paired with empowering and agentic narratives of migrants. Previous research has shown that portraying migrant essential workers as empowered exemplars during the COVID‐19 pandemic led to more positive perceptions and activated self‐transcendent emotions, fostering positive attitudes and a greater willingness to help (Alonso‐Arbiol et al., 2024).

However, our research also involves potential risks and less optimistic implications. It suggests that the host society may condition the acceptance and naturalization of human migration on the emotions migrants express, ‘successful’ adaptation or demonstrated agency. This aligns with research showing that the civic inclusion of migrants is often contingent upon cultural assimilation (Kadianaki & Andreouli, 2017). Such conditional acceptance can impose unfair expectations, as immigrants, like any other members of society, should have the right to express a full range of emotions, including frustration and anger, without their acceptance, support and ‘humanness’ being compromised.

In line with previous research, our findings reveal the creation of social hierarchies based on assimilation, exemplified by the expectation to ‘smile’ or otherwise perform positivity, which determines who is deemed acceptable within society (Larin, 2020). This expectation mirrors the broader societal tendency, where the effort made to display positive emotions is disproportionately rewarded and negative emotions are stigmatized (van Zyl et al., 2024). These assimilation pressures can lead to significant psychological stress for immigrants, as they are forced to suppress authentic emotions to fit societal norms, potentially exacerbating feelings of isolation and marginalization (Birman & Simon, 2014).

Therefore, critical intercultural policies and societal attitudes that promote and naturalize diversity are essential, and this includes the recognition and validation of the totality of different emotional experiences of immigrants. This means shifting away from conditional acceptance based on specific behavioural requirements imposed by the host societies and towards a more equitable framework that respects the diverse emotional expressions of all community members.

Finally, we would like to acknowledge our positionality in this research: Three of the authors are immigrants themselves, albeit privileged, White individuals. One is Polish living in Spain, another is Chilean living in Spain, and the third is Polish living in Australia. We stress that future research should involve authentic images and listen to the voices of diverse immigrant individuals, using a more participatory approach. This is crucial for raising awareness about actual lived experiences and fostering a deeper understanding and connection between immigrants and host culture members.

## AUTHOR CONTRIBUTIONS

**Magdalena Bobowik:** Conceptualization; methodology; investigation; validation; supervision; funding acquisition; writing – original draft; project administration. **José J. Pizarro:** Methodology; software; data curation; investigation; validation; formal analysis; writing – original draft. **Patrycja Slawuta:** Conceptualization; methodology; investigation; writing – review and editing. **Nekane Basabe:** Investigation; project administration; resources; writing – review and editing.

## CONFLICT OF INTEREST STATEMENT

The authors declare no conflicts of interest.

## Supporting information


supporting information

## Data Availability

Data and materials from this research are available at https://osf.io/9gh5w/ (non‐anonymous link with access to all components of the project).

## Footnotes

^1^
The selection of the images was based on the criteria of two experts and their subsequent piloting with 17 participants, who evaluated perceived prototypicality of the Moroccan man, emotional attributes (i.e. neutrality, happiness, sorrow, anger) and how credible these expressions were.

^2^
The images were selected by two experts, based on the perceived prototypicality of the target as Pakistani.

^3^
We employed the material and procedure applied inBobowik et al. 2023 to manipulate group membership (i.e. the same individual from The Karolinska directed emotional faces – KDEF; Lundqvist et al., 1998).

^4^
In the original pre‐registration, we indicated that four photographs would be used per group. However, based on the validation results and to enhance ecological validity, we decided to include five photographs for each group category.

^5^
The pooled indirect effect of smiling on approach intentions reflects the effect of each of the mediators alone, not controlling for other mediators.
