# Supplementary material for: Smiling across borders: Host culture members' reactions to happiness expressed by immigrants
Source: Br J Soc Psychol. 2026 Jan 7;65(1):e70030. doi: 10.1111/bjso.70030 (PMC12780317; doi:10.1111/bjso.70030)
Supplement: Supplementary file 1 — supporting information [file BJSO-65-0-s001.docx]

**Smiling Across Borders: Host Culture Members’ Reactions to Happiness Expressed by Immigrants**

[Supplementary Online Material]

This document includes:

Section I^[[1]](#footnote-1)^

- Study 1: Descriptive Data, Bivariate Correlations, and Path Analyses.

Section II

- Study 2ab: Descriptive Data, Bivariate Correlations, and Path Analyses.

Section III

- Study 3: Descriptive Data, Bivariate Correlations, and MG-Path Analyses.

Section IV

- Study 4: Stimuli Validation, Descriptive Data, Bivariate Correlations, and MG-Path Analyses.

# Section I Descriptive Data, Bivariate Correlations, and Path Analyses in Study 1

Section I provides complementary analyses for Study 1, expanding on those in the main manuscript. These include descriptive analyses, bivariate correlations (Supplementary Table S1.1), and path analyses examining the effects of smiling on approach and avoidance intentions via cognitive (Supplementary Table S1.2) and affective mechanisms (Supplementary Table S1.3).

The exploratory path analyses tested the simultaneous effects of multiple mediators on both behavioral intention variables. Two path analyses were conducted, with the condition (neutral display coded as 0, smiling as 1) as the independent variable, and approach and avoidance intentions as the dependent variables. Mediators included cognitive inferences (perceived warmth and competence) and affective responses (joy, liking, anger). All details are presented in the R code available on the OSF project website (<https://osf.io/9gh5w/>).

**Supplementary Table S1.1**

*Descriptive Statistics and Bivariate Correlation Analyses with Main Variables (Study 1).*

| Variable | *M* | *SD* | 1 | 2 | 3 | 4 | 5 | 6 | 7 | 8 |
| --- | --- | --- | --- | --- | --- | --- | --- | --- | --- | --- |
|  |  |  |  |  |  |  |  |  |  |  |
| 1. Gender | - | - |  |  |  |  |  |  |  |  |
|  |  |  |  |  |  |  |  |  |  |  |
| 2. Age | 21.82 | 3.79 | .22* |  |  |  |  |  |  |  |
|  |  |  | [.05, .38] |  |  |  |  |  |  |  |
| 3. Warmth | 4.67 | 2.17 | .05 | .02 |  |  |  |  |  |  |
|  |  |  | [-.12, .23] | [-.16, .20] |  |  |  |  |  |  |
| 4. Competence | 5.36 | 1.73 | .08 | -.01 | .65** |  |  |  |  |  |
|  |  |  | [-.10, .25] | [-.18, .17] | [.53, .74] |  |  |  |  |  |
| 5. Joy | 2.35 | 1.86 | .06 | -.08 | .48** | .41** |  |  |  |  |
|  |  |  | [-.12, .23] | [-.25, .10] | [.33, .60] | [.25, .55] |  |  |  |  |
| 6. Liking | 3.69 | 1.65 | .15 | -.06 | .60** | .50** | .65** |  |  |  |
|  |  |  | [-.02, .32] | [-.23, .12] | [.48, .70] | [.36, .63] | [.53, .74] |  |  |  |
| 7. Anger | 1.72 | 1.21 | .14 | -.01 | -.05 | -.06 | -.20* | -.16 |  |  |
|  |  |  | [-.03, .31] | [-.18, .17] | [-.23, .12] | [-.24, .11] | [-.36, -.02] | [-.32, .02] |  |  |
| 8. Approach | 4.42 | 1.75 | -.00 | .02 | .38** | .26** | .16 | .41** | .13 |  |
|  |  |  | [-.18, .17] | [-.15, .20] | [.22, .52] | [.09, .42] | [-.02, .33] | [.26, .55] | [-.05, .30] |  |
| 9. Avoidance | 1.32 | 0.55 | -.11 | -.02 | -.27** | -.18* | -.15 | -.27** | -.11 | -.46** |
|  |  |  | [-.28, .07] | [-.20, .15] | [-.43, -.10] | [-.35, -.01] | [-.32, .03] | [-.43, -.10] | [-.28, .07] | [-.58, -.30] |

*Note.* Approach = Approach intentions; Avoidance = Avoidance intentions*. M* and *SD* are used to represent mean and standard deviation, respectively. Gender is coded 1 for women and 2 for men. Values in square brackets indicate the 95% confidence interval for each correlation. * indicates *p* < .05. ** indicates *p* < .01.

**Supplementary Table S1.2**

*Path Analyses of the Effects of Smiling on Approach and Avoidance Intentions via Inferences of Warmth and Competence (Study 1).*

| Effect | *B* | *SE* | *p* | 95% CI | | | β |
| --- | --- | --- | --- | --- | --- | --- | --- |
|  |  |  |  | *LL* | *UL* | |  |
| *Approach intentions* | | | | | | | |
| Total effect | 0.204 | 0.441 | .644 | -0.660 | 1.067 | | .060 |
| Direct effects |  |  |  |  |  | |  |
| Condition on approach | -0.464 | 0.464 | .318 | -1.373 | 0.446 | | -.137 |
| Warmth on approach | 0.353 | 0.134 | .009 | 0.090 | 0.616 | | .447 |
| Competence on approach | -0.039 | 0.155 | .799 | -0.343 | 0.264 | | -.041 |
| Condition on warmth | 2.025 | 0.494 | < .001 | 1.057 | 2.992 | | .471 |
| Condition on competence | 1.196 | 0.428 | .005 | 0.358 | 2.035 | | .342 |
| Indirect effects via |  |  |  |  |  | |  |
| Warmth | 0.714 | 0.323 | .027 | 0.082 | 1.347 | | .211 |
| Competence | -0.047 | 0.186 | .800 | -0.412 | 0.317 | | -.014 |
| *Avoidance intentions* | | | | | | | |
| Total effect | -0.083 | 0.108 | .443 | -0.294 | 0.129 | | -.099 |
| Direct effects |  |  |  |  |  | |  |
| Condition on avoidance | -0.011 | 0.121 | .925 | -0.248 | 0.225 | | -.014 |
| Warmth on avoidance | -0.039 | 0.035 | .260 | -0.108 | 0.029 | | -.203 |
| Competence on avoidance | 0.007 | 0.040 | .864 | -0.072 | 0.086 | | .029 |
| Indirect effects via |  |  |  |  |  | |  |
| Warmth | -0.080 | 0.073 | .278 | -0.224 | 0.064 | | -.096 |
| Competence | 0.008 | 0.048 | .864 | -0.086 | 0.103 | | .010 |
| *Model fit* | | | | | |  | |
| *CFI* | 1.000 | | | | | | |
| *TLI* | 1.000 | | | | | | |
| *RMSEA* (90% *CI*) | 0.000 (0.000, 0.000) | | | | | | |
| *SRMR* | 0.000 | | | | | | |

*Note.* Condition = Neutral vs. Smiling Display; Approach = Approach intentions; Avoidance = Avoidance intentions; *B* = Non-standardized estimates; *SE* = Standard error; β = standardized coefficients of the model; *CFI* = Comparative Fit Index; *TLI* = Tucker-Lewis Index; *RMSEA* = Root Mean Square Error of Approximation; *SRMR* = Standardized Root Mean Square Residual.

To analyze the effects of smiling on the host culture members’ behavioral intentions, we conducted a path analysis with two simultaneous dependent variables (i.e., approach and avoidance intentions). The experimental condition (i.e., the smiling display) was tested against the control condition (i.e., the neutral display). The results showed significant indirect effects of smiling (compared to the neutral condition) on approach (but not avoidance) intentions through warmth inferences.

**Supplementary Table S1.3**

*Path Analyses of the Effects of Smiling on Approach and Avoidance Intentions via Host Culture Members’ Affective Reactions (Study 1).*

| Effect | *B* | *SE* | *p* | 95% CI | | β |
| --- | --- | --- | --- | --- | --- | --- |
|  |  |  |  | *LL* | *UL* |  |
| *Approach intentions* | | | | | | |
| Total effect | 0.137 | 0.444 | .758 | -0.734 | 1.008 | .040 |
| Direct effects |  |  |  |  |  |  |
| Condition on approach | -0.467 | 0.576 | .418 | -1.597 | 0.663 | -.136 |
| Joy on approach | -0.208 | 0.157 | .186 | -0.517 | 0.100 | -.252 |
| Liking on approach | 0.829 | 0.154 | < .001 | 0.528 | 1.131 | .772 |
| Anger on approach | 0.269 | 0.173 | .120 | -0.070 | 0.608 | .168 |
| Condition on joy | 3.246 | 0.338 | < .001 | 2.583 | 3.909 | .778 |
| Condition on liking | 1.760 | 0.347 | < .001 | 1.081 | 2.439 | .548 |
| Condition on anger | -0.666 | 0.265 | .012 | -1.184 | -0.147 | -.309 |
| Indirect effects via |  |  |  |  |  |  |
| Joy | -0.676 | 0.516 | .190 | -1.687 | 0.335 | -.196 |
| Liking | 1.459 | 0.395 | < .001 | 0.686 | 2.233 | .424 |
| Anger | -0.179 | 0.135 | .186 | -0.445 | 0.086 | -.052 |
| *Avoidance intentions* | | | | | | |
| Total effect | -0.075 | 0.107 | .481 | -0.285 | 0.134 | -.091 |
| Direct effects |  |  |  |  |  |  |
| Condition on avoidance | -0.121 | 0.167 | .467 | -0.449 | 0.206 | -.146 |
| Anger on avoidance | -0.051 | 0.050 | .305 | -0.150 | 0.047 | -.133 |
| Joy on avoidance | 0.053 | 0.046 | .249 | -0.037 | 0.142 | .263 |
| Liking on avoidance | -0.090 | 0.045 | .043 | -0.177 | -0.003 | -.348 |
| Indirect effects via |  |  |  |  |  |  |
| Joy | 0.171 | 0.149 | .252 | -0.122 | 0.463 | .205 |
| Liking | -0.159 | 0.084 | .060 | -0.324 | 0.007 | -.191 |
| Anger | 0.034 | 0.036 | .342 | -0.036 | 0.105 | .041 |
| *CFI* | 1.000 | | | | | |
| *TLI* | 1.000 | | | | | |
| *RMSEA* (90% *CI*) | 0.000 (0.000, 0.291) | | | | | |
| *SRMR* | 0.019 | | | | | |

*Note.* Condition = Neutral vs. Smiling Display; Approach = Approach intentions; Avoidance = Avoidance intentions; *B* = Non-standardized estimates; *SE* = Standard error; β = standardized coefficients of the model; *CFI* = Comparative Fit Index; *TLI* = Tucker-Lewis Index; *RMSEA* = Root Mean Square Error of Approximation; *SRMR* = Standardized Root Mean Square Residual.

Additionally, we found that smiling had indirect effects on approach but not avoidance intentions through liking.

# Section II Study 2ab: Descriptive Data, Bivariate Correlations, and MG-Path Analyses

Section II presents complementary analyses for Study 2ab, expanding on those in the main manuscript. These include descriptive analyses and bivariate correlations (Supplementary Tables S2.1 and S2.2 for Studies 2a and 2b, respectively) and multi-group path analyses examining the effects of smiling on approach and avoidance intentions via cognitive (Supplementary Table S2.3) and affective mechanisms (Supplementary Table S2.4).

The exploratory multi-group path analyses tested the simultaneous effects of multiple mediators on both behavioral intention variables. Specifically, two path analyses were conducted, with the condition (neutral display coded as 0, smiling as 1) as the independent variable, and approach and avoidance intentions as dependent variables. In the first model, perceived warmth and competence were included as parallel, correlated mediators. In the second, trust, anger, and joy were introduced as parallel, correlated mediators. Bootstrapping was used to calculate confidence intervals.

We used sample/country (Spain vs. the United States) as the grouping factor to simultaneously test predicted effects, comparing a constrained versus an unconstrained model. The unconstrained model showed a better fit (χ²(8, *N* = 151) = 21.58, *p* = .006). Therefore, we present the results for the unconstrained model, where all regression paths were freely estimated across both studies. Additionally, we provide a summary of the findings from the constrained models.

Full details are available in the R code on the OSF project website (<https://osf.io/9gh5w/>).

**Supplementary Table S2.1**

*Descriptive Statistics and Bivariate Correlation Analyses with Main Variables (Study 2a).*

| Variable | *M* | *SD* | 1 | 2 | 3 | 4 | 5 | 6 | 7 | 8 |
| --- | --- | --- | --- | --- | --- | --- | --- | --- | --- | --- |
|  |  |  |  |  |  |  |  |  |  |  |
| 1. Gender | - | - |  |  |  |  |  |  |  |  |
|  |  |  |  |  |  |  |  |  |  |  |
| 2. Age | 22.80 | 1.66 | .07 |  |  |  |  |  |  |  |
|  |  |  | [-.14, .28] |  |  |  |  |  |  |  |
| 3. Warmth | 4.88 | 2.23 | .12 | -.01 |  |  |  |  |  |  |
|  |  |  | [-.09, .32] | [-.22, .20] |  |  |  |  |  |  |
| 4. Competence | 5.14 | 2.04 | .03 | -.02 | .53** |  |  |  |  |  |
|  |  |  | [-.18, .24] | [-.22, .19] | [.36, .66] |  |  |  |  |  |
| 5. Joy | 3.06 | 1.48 | .16 | -.04 | .68** | .54** |  |  |  |  |
|  |  |  | [-.06, .35] | [-.25, .17] | [.55, .78] | [.38, .68] |  |  |  |  |
| 6. Liking | 3.71 | 1.66 | .15 | -.06 | .79** | .52** | .86** |  |  |  |
|  |  |  | [-.06, .35] | [-.27, .15] | [.69, .86] | [.35, .66] | [.79, .90] |  |  |  |
| 7. Anger | 1.24 | 0.62 | -.03 | -.05 | -.15 | -.14 | -.10 | -.18 |  |  |
|  |  |  | [-.24, .18] | [-.25, .17] | [-.35, .06] | [-.34, .07] | [-.31, .11] | [-.38, .03] |  |  |
| 8. Approach | 2.89 | 1.38 | .21* | -.01 | .44** | .38** | .60** | .63** | .02 |  |
|  |  |  | [.00, .40] | [-.22, .20] | [.26, .60] | [.18, .55] | [.45, .72] | [.48, .74] | [-.19, .22] |  |
| 9. Avoidance | 1.86 | 1.13 | .06 | -.02 | -.27* | -.14 | -.23* | -.38** | .29** | -.23* |
|  |  |  | [-.16, .26] | [-.23, .19] | [-.45, -.06] | [-.34, .07] | [-.42, -.02] | [-.55, -.19] | [.08, .47] | [-.42, -.02] |

*Note.* Approach = Approach intentions; Avoidance = Avoidance intentions*. M* and *SD* are used to represent mean and standard deviation, respectively. Gender is coded 1 for women and 2 for men. Values in square brackets indicate the 95% confidence interval for each correlation. * indicates *p* < .05. ** indicates *p* < .01.

**Supplementary Table S2.2**

*Descriptive Statistics and Bivariate Correlation Analyses with Main Variables (Study 2b).*

| Variable | *M* | *SD* | 1 | 2 | 3 | 4 | 5 | 6 | 7 | 8 |
| --- | --- | --- | --- | --- | --- | --- | --- | --- | --- | --- |
|  |  |  |  |  |  |  |  |  |  |  |
| 1. Gender | 1.41 | 0.50 |  |  |  |  |  |  |  |  |
|  |  |  |  |  |  |  |  |  |  |  |
| 2. Age | 36.35 | 12.74 | -.17 |  |  |  |  |  |  |  |
|  |  |  | [-.40, .08] |  |  |  |  |  |  |  |
| 3. Warmth | 5.97 | 2.51 | -.09 | -.05 |  |  |  |  |  |  |
|  |  |  | [-.33, .16] | [-.30, .20] |  |  |  |  |  |  |
| 4. Competence | 6.68 | 2.11 | -.07 | -.11 | .81** |  |  |  |  |  |
|  |  |  | [-.32, .18] | [-.35, .14] | [.70, .88] |  |  |  |  |  |
| 5. Joy | 3.18 | 1.70 | -.03 | -.11 | .61** | .46** |  |  |  |  |
|  |  |  | [-.28, .22] | [-.35, .14] | [.43, .75] | [.24, .63] |  |  |  |  |
| 6. Liking | 3.40 | 1.88 | -.07 | -.03 | .74** | .57** | .86** |  |  |  |
|  |  |  | [-.31, .18] | [-.27, .22] | [.60, .83] | [.37, .71] | [.77, .91] |  |  |  |
| 7. Anger | 1.40 | 0.80 | .28* | -.09 | -.23 | -.30* | .00 | -.01 |  |  |
|  |  |  | [.04, .50] | [-.33, .16] | [-.45, .02] | [-.51, -.06] | [-.25, .25] | [-.26, .24] |  |  |
| 8. Approach | 3.43 | 1.67 | -.11 | -.31* | .60** | .59** | .60** | .68** | -.07 |  |
|  |  |  | [-.35, .14] | [-.52, -.07] | [.41, .74] | [.40, .73] | [.41, .74] | [.52, .80] | [-.31, .19] |  |
| 9. Avoidance | 2.09 | 1.59 | .17 | .10 | -.49** | -.50** | -.22 | -.35** | .42** | -.44** |
|  |  |  | [-.08, .40] | [-.15, .34] | [-.66, -.28] | [-.67, -.29] | [-.44, .03] | [-.55, -.11] | [.19, .60] | [-.62, -.22] |

*Note.* Approach = Approach intentions; Avoidance = Avoidance intentions*. M* and *SD* are used to represent mean and standard deviation, respectively. Gender is coded 1 for women and 2 for men. Values in square brackets indicate the 95% confidence interval for each correlation. * indicates *p* < .05. ** indicates *p* < .01

**Supplementary Table S2.3**

*Multi-group Path Analyses of the Effects of Smiling on Approach and Avoidance Intentions via Inferences of Warmth and Competence (Study 2a and 2b).*

|  | Study 2a | | | | |  |  | Study 2b | | | | |  |
| --- | --- | --- | --- | --- | --- | --- | --- | --- | --- | --- | --- | --- | --- |
| Effect | *B* | *SE* | *p* | 95% CI | | β |  | *B* | *SE* | *p* | 95% CI | | β |
|  |  |  |  | *LL* | *UL* |  |  |  |  |  | *LL* | *UL* |  |
| *Approach Intentions* | | | | | | | | | | | | | |
| Total Effect | 0.456 | 0.290 | .116 | -0.113 | 1.025 | .165 |  | 1.327 | 0.383 | .001 | 0.577 | 2.078 | .400 |
| Direct Effects |  |  |  |  |  |  |  |  |  |  |  |  |  |
| Condition on approach | -0.345 | 0.331 | .298 | -0.994 | 0.304 | -.125 |  | -0.037 | 0.491 | .940 | -0.999 | 0.924 | -.011 |
| Warmth on approach | 0.262 | 0.086 | .002 | 0.093 | 0.431 | .423 |  | 0.242 | 0.146 | .097 | -0.044 | 0.528 | .364 |
| Competence on approach | 0.122 | 0.076 | .111 | -0.028 | 0.271 | .179 |  | 0.235 | 0.137 | .087 | -0.034 | 0.504 | .297 |
| Condition on warmth | 2.704 | 0.376 | < .001 | 1.966 | 3.442 | .608 |  | 3.630 | 0.432 | < .001 | 2.783 | 4.477 | .727 |
| Condition on avoidance | 0.754 | 0.426 | .076 | -0.080 | 1.589 | .186 |  | 2.067 | 0.459 | < .001 | 1.167 | 2.966 | .493 |
| Indirect effects via |  |  |  |  |  |  |  |  |  |  |  |  |  |
| Warmth | 0.709 | 0.253 | .005 | 0.213 | 1.206 | .257 |  | 0.878 | 0.540 | .104 | -0.179 | 1.936 | .265 |
| Competence | 0.092 | 0.077 | .236 | -0.060 | 0.243 | .033 |  | 0.486 | 0.304 | .109 | -0.109 | 1.081 | .147 |
| *Avoid Intentions* | | | | | | | | | | | | | |
| Total effect | -0.731 | 0.227 | .001 | -1.177 | -0.286 | -.324 |  | -0.502 | 0.392 | .200 | -1.269 | 0.266 | -.159 |
| Direct effects |  |  |  |  |  |  |  |  |  |  |  |  |  |
| Condition on avoidance | -0.604 | 0.291 | .038 | -1.173 | -0.034 | -.268 |  | 1.191 | 0.484 | .014 | 0.242 | 2.140 | .378 |
| Warmth on avoidance | -0.039 | 0.076 | .603 | -0.188 | 0.109 | -.078 |  | -0.380 | 0.144 | .008 | -0.662 | -0.098 | -.603 |
| Competence on avoidance | -0.028 | 0.067 | .671 | -0.160 | 0.103 | -.051 |  | -0.151 | 0.136 | .265 | -0.417 | 0.114 | -.201 |
| Indirect effects via |  |  |  |  |  |  |  |  |  |  |  |  |  |
| Warmth | -0.106 | 0.205 | .604 | -0.508 | 0.296 | -.047 |  | -1.380 | 0.548 | .012 | -2.454 | -0.307 | -.438 |
| Competence | -0.021 | 0.052 | .680 | -0.123 | 0.080 | -.010 |  | -0.312 | 0.288 | .279 | -0.878 | 0.253 | -.099 |
| *Model fit* | | | | | | | | | | | | | |
| *CFI* | 1.000 | | | | | | | | | | | | |
| *TLI* | 1.000 | | | | | | | | | | | | |
| *RMSEA* (90% CI) | 0.000 (0.000, 0.000) | | | | | | | | | | | | |
| *SRMR* | 0.000 | | | | | | | | | | | | |

*Note.* Study 2a: a Moroccan target (Spain); Study 2b: a Pakistani target (the US). Condition = Neutral vs. Smiling Display; Approach = Approach intentions; Avoidance = Avoidance intentions; *B* = Non-standardized estimates; *SE* = Standard error; β = standardized coefficients of the model; *CFI* = Comparative Fit Index; *TLI* = Tucker-Lewis Index; *RMSEA* = Root Mean Square Error of Approximation; *SRMR* = Standardized Root Mean Square Residual.

We found significant indirect effects of smiling via warmth on approach intentions in Study 2a, and indirect effects of smiling via competence on avoiding intentions in Study 2b. When testing the fully constraint model of effects, we found indirect effects of smiling via warmth and competence on both approach intentions. However, the overall fit was poorer (*CFI* = .949, *TLI* = .873, *RMSEA* = .150, 90%*CI* [.08, .23], *SRMR* = .116; see R code in SOM).

**Supplementary Table S2.4**

*Path Analyses of the Effects of Smiling on Approach and Avoidance Intentions via Host Culture Members’ Affective Reactions (Study 2a and 2b).*

|  | Study 2a | | | | |  |  | Study 2b | | | | |  |
| --- | --- | --- | --- | --- | --- | --- | --- | --- | --- | --- | --- | --- | --- |
| Effect | *B* | *SE* | *p* | 95% CI | | β |  | *B* | *SE* | *p* | 95% CI | | β |
|  |  |  |  | *LL* | *UL* |  |  |  |  |  | *LL* | *UL* |  |
| *Approach intentions* | | | | | | | | | | | | | |
| Total effect | 0.456 | 0.294 | .121 | -0.120 | 1.032 | .163 |  | 1.327 | 0.384 | .001 | 0.575 | 2.080 | .399 |
| Direct effects |  |  |  |  |  |  |  |  |  |  |  |  |  |
| Condition on approach | -0.725 | 0.256 | .005 | -1.227 | -0.223 | -.259 |  | -0.268 | 0.399 | .502 | -1.051 | 0.515 | -.081 |
| Joy on approach | 0.255 | 0.141 | .072 | -0.023 | 0.532 | .269 |  | 0.062 | 0.175 | .724 | -0.281 | 0.405 | .063 |
| Liking on approach | 0.466 | 0.129 | < .001 | 0.212 | 0.719 | .551 |  | 0.604 | 0.165 | < .001 | 0.279 | 0.928 | .680 |
| Anger on approach | 0.281 | 0.173 | .105 | -0.059 | 0.620 | .124 |  | -0.133 | 0.192 | .487 | -0.509 | 0.243 | -.064 |
| Condition on joy | 1.490 | 0.271 | < .001 | 0.958 | 2.022 | .505 |  | 2.003 | 0.341 | < .001 | 1.335 | 2.671 | .595 |
| Condition on liking | 1.783 | 0.297 | < .001 | 1.202 | 2.365 | .540 |  | 2.412 | 0.360 | < .001 | 1.706 | 3.119 | .645 |
| Condition on anger | -0.102 | 0.132 | .438 | -0.360 | 0.156 | -.082 |  | -0.118 | 0.199 | .553 | -0.508 | 0.272 | -.075 |
| Indirect effects via |  |  |  |  |  |  |  |  |  |  |  |  |  |
| Joy | 0.379 | 0.222 | .087 | -0.055 | 0.814 | .136 |  | 0.124 | 0.351 | .724 | -0.564 | 0.812 | .037 |
| Liking | 0.831 | 0.269 | .002 | 0.303 | 1.358 | .297 |  | 1.456 | 0.455 | .001 | 0.565 | 2.347 | .438 |
| Anger | -0.029 | 0.041 | .484 | -0.109 | 0.052 | -.010 |  | 0.016 | 0.035 | .652 | -0.053 | 0.084 | .005 |
| *Avoid intentions* | | | | | | | | | | | | | |
| Total effect | -0.731 | 0.224 | .001 | -1.171 | -0.291 | -.328 |  | -0.502 | 0.394 | .203 | -1.273 | 0.270 | -.158 |
| Direct effects |  |  |  |  |  |  |  |  |  |  |  |  |  |
| Condition on approach | -0.435 | 0.249 | .081 | -0.923 | 0.053 | -.195 |  | 0.448 | 0.429 | .297 | -0.394 | 1.289 | .141 |
| Joy on approach | 0.276 | 0.138 | .045 | 0.007 | 0.546 | .366 |  | 0.235 | 0.188 | .211 | -0.134 | 0.604 | .250 |
| Liking on approach | -0.375 | 0.126 | .003 | -0.622 | -0.129 | -.557 |  | -0.548 | 0.178 | .002 | -0.897 | -0.199 | -.648 |
| Anger on approach | 0.375 | 0.169 | .026 | 0.045 | 0.706 | .209 |  | 0.836 | 0.206 | < .001 | 0.432 | 1.240 | .419 |
| Indirect effects via |  |  |  |  |  |  |  |  |  |  |  |  |  |
| Joy | 0.412 | 0.218 | .059 | -0.016 | 0.839 | .185 |  | 0.471 | 0.385 | .222 | -0.284 | 1.226 | .149 |
| Liking | -0.670 | 0.251 | .008 | -1.161 | -0.178 | -.300 |  | -1.321 | 0.473 | .005 | -2.247 | -0.395 | -.418 |
| Anger | -0.038 | 0.052 | .464 | -0.141 | 0.064 | -.017 |  | -0.099 | 0.168 | .557 | -0.428 | 0.231 | -.031 |
| *Model fit* | | | | | | | | | | | | | |
| *CFI* | 1.000 | | | | | | | | | | | | |
| *TLI* | 1.000 | | | | | | | | | | | | |
| *RMSEA* (90% CI) | 0.000 (0.000, 0.167) | | | | | | | | | | | | |
| *SRMR* | 0.028 | | | | | | | | | | | | |

*Note.* Study 2a: a Moroccan target (Spain); Study 2b: a Pakistani target (the US). Condition = Neutral vs. Smiling Display; Approach = Approach intentions; Avoidance = Avoidance intentions; *B* = Non-standardized estimates; *SE* = Standard error; β = standardized coefficients of the model; *CFI* = Comparative Fit Index; *TLI* = Tucker-Lewis Index; *RMSEA* = Root Mean Square Error of Approximation; *SRMR* = Standardized Root Mean Square Residual.

We found significant indirect effects of smiling via liking on approach and avoiding intentions in both studies. In addition, we found marginally significant effects through joy on approach (*p* = .087) and avoidance intentions (*p* = .059) in Study 2a. When replicating the models using a fully constraint one (see SOM), we found the same indirect effects of smiling on approach and avoiding intentions via liking, as well as an indirect effect of smiling on less avoidance intentions via joy (*CFI* = .996, *TLI* = .993, *RMSEA* = .035, 90%*CI* [.00, .12], *SRMR* = .068).

# Section III Study 3: Descriptive Data, Bivariate Correlations, and MG-Path Analyses

Section III presents complementary analyses for Study 3, expanding on those in the main manuscript. These include descriptive analyses and bivariate correlations (Supplementary Table S3.1, Supplementary Table S3.2, and Supplementary Table S3.3 for the total sample, ingroup condition and outgroup condition, respectively). We also conducted multi-group path analyses examining the effects of smiling on approach and avoidance intentions via cognitive (Supplementary Table S3.4) and affective mechanisms (Supplementary Table S3.5), and the effects of smiling on donating and volunteering intentions via cognitive (Supplementary Table S3.6) and affective mechanisms (Supplementary Table S3.7).

The exploratory multi-group path analyses tested the simultaneous effects of multiple mediators on both behavioral intention variables. Specifically, two path analyses were conducted, with the condition (neutral display coded as 0, smiling as 1) as the independent variable, and approach and avoidance intentions as dependent variables. In the first model, perceived warmth and competence were included as parallel, correlated mediators. In the second, trust, anger, and joy were introduced as parallel, correlated mediators. Bootstrapping was used to calculate confidence intervals. We used immigrant (outgroup member) vs. host culture member (ingroup member) factor as the grouping factor to simultaneously test predicted effects, comparing a constrained versus an unconstrained model. We found no significant differences among the models (χ^2^(8, *N* = 169) = 13.04, *p* = .110) and decided to report the results of the unconstrained one, where all regression paths were freely estimated across both group categories, due to its better fit indexes Additionally, we provide a summary of the findings from the constrained models.

We additionally tested a MG-path model to test indirect effects of smiling on *kama muta* and admiration via warmth and competence (Supplementary Table S3.8).

Full details are available in the R code on the OSF project website (<https://osf.io/9gh5w/>).

**Supplementary Table S3.1**

*Descriptive Statistics and Correlation Analyses with Main Variables (Study 3, Total Sample).*

| Variable | *M* | *SD* | 1 | 2 | 3 | 4 | 5 | 6 | 7 | 8 | 9 | 10 | 11 |
| --- | --- | --- | --- | --- | --- | --- | --- | --- | --- | --- | --- | --- | --- |
|  |  |  |  |  |  |  |  |  |  |  |  |  |  |
| 1. Gender | - | - |  |  |  |  |  |  |  |  |  |  |  |
|  |  |  |  |  |  |  |  |  |  |  |  |  |  |
| 2. Age | 23.66 | 8.82 | -.12 |  |  |  |  |  |  |  |  |  |  |
|  |  |  | [-.27, .03] |  |  |  |  |  |  |  |  |  |  |
| 3. Warmth | 4.30 | 1.43 | -.14 | -.00 |  |  |  |  |  |  |  |  |  |
|  |  |  | [-.28, .02] | [-.15, .15] |  |  |  |  |  |  |  |  |  |
| 4. Competence | 4.29 | 0.98 | .08 | .03 | .51** |  |  |  |  |  |  |  |  |
|  |  |  | [-.07, .23] | [-.12, .18] | [.38, .61] |  |  |  |  |  |  |  |  |
| 5. Liking | 2.92 | 1.44 | -.07 | .19* | .49** | .39** |  |  |  |  |  |  |  |
|  |  |  | [-.21, .09] | [.04, .33] | [.37, .60] | [.26, .51] |  |  |  |  |  |  |  |
| 6. Kama muta | 2.54 | 1.49 | .02 | .24** | .33** | .18* | .53** |  |  |  |  |  |  |
|  |  |  | [-.13, .17] | [.09, .37] | [.19, .46] | [.03, .33] | [.42, .63] |  |  |  |  |  |  |
| 7. Admiration | 2.72 | 1.79 | -.09 | .06 | .45** | .39** | .60** | .40** |  |  |  |  |  |
|  |  |  | [-.24, .06] | [-.09, .21] | [.32, .56] | [.25, .51] | [.50, .69] | [.27, .52] |  |  |  |  |  |
| 8. Anger | 1.42 | 0.87 | .01 | .09 | -.20* | -.14 | .10 | .29** | .08 |  |  |  |  |
|  |  |  | [-.14, .16] | [-.06, .24] | [-.34, -.05] | [-.28, .01] | [-.05, .25] | [.14, .42] | [-.07, .23] |  |  |  |  |
| 9. Approach | 3.96 | 1.54 | -.04 | -.05 | .54** | .44** | .56** | .33** | .48** | -.03 |  |  |  |
|  |  |  | [-.19, .11] | [-.20, .10] | [.43, .64] | [.31, .56] | [.45, .66] | [.19, .46] | [.35, .59] | [-.18, .12] |  |  |  |
| 10. Avoidance | 1.84 | 1.05 | .01 | .03 | -.35** | -.20** | -.24** | -.03 | -.19* | .26** | -.32** |  |  |
|  |  |  | [-.14, .16] | [-.12, .18] | [-.48, -.21] | [-.35, -.06] | [-.38, -.10] | [-.18, .12] | [-.33, -.04] | [.12, .40] | [-.45, -.18] |  |  |
| 11. Donation | 2.86 | 1.52 | .04 | -.09 | .12 | .10 | .23** | .22** | .26** | .02 | .24** | .05 |  |
|  |  |  | [-.11, .19] | [-.24, .06] | [-.03, .27] | [-.06, .25] | [.08, .37] | [.07, .37] | [.11, .40] | [-.13, .18] | [.09, .38] | [-.11, .20] |  |
| 12. Volunteering | 4.18 | 1.73 | .13 | .06 | .19* | .22** | .25** | .31** | .32** | .07 | .25** | -.20* | .26** |
|  |  |  | [-.02, .28] | [-.10, .21] | [.04, .34] | [.07, .37] | [.10, .39] | [.17, .44] | [.17, .45] | [-.09, .22] | [.10, .39] | [-.34, -.04] | [.11, .40] |

*Note.* Approach = Approach intentions; Avoidance = Avoidance intentions; Donation = Donation intentions; Volunteering = Volunteering intentions*. M* and *SD* are used to represent mean and standard deviation, respectively. Gender is coded 1 for women and 2 for men. Values in square brackets indicate the 95% confidence interval for each correlation. * indicates *p* < .05. ** indicates *p* < .01.

**Supplementary Table S3.2**

*Descriptive Statistics and Correlation Analyses with Main Variables (Study 3, Group membership = Ingroup).*

| Variable | *M* | *SD* | 1 | 2 | 3 | 4 | 5 | 6 | 7 | 8 | 9 | 10 | 11 |
| --- | --- | --- | --- | --- | --- | --- | --- | --- | --- | --- | --- | --- | --- |
|  |  |  |  |  |  |  |  |  |  |  |  |  |  |
| 1. Gender | - | - |  |  |  |  |  |  |  |  |  |  |  |
|  |  |  |  |  |  |  |  |  |  |  |  |  |  |
| 2. Age | 23.41 | 9.11 | -.05 |  |  |  |  |  |  |  |  |  |  |
|  |  |  | [-.26, .17] |  |  |  |  |  |  |  |  |  |  |
| 3. Warmth | 4.12 | 1.36 | -.02 | -.09 |  |  |  |  |  |  |  |  |  |
|  |  |  | [-.23, .20] | [-.30, .13] |  |  |  |  |  |  |  |  |  |
| 4. Competence | 4.12 | 0.88 | .16 | -.04 | .31** |  |  |  |  |  |  |  |  |
|  |  |  | [-.06, .37] | [-.25, .18] | [.10, .49] |  |  |  |  |  |  |  |  |
| 5. Liking | 2.66 | 1.29 | -.03 | .13 | .44** | .34** |  |  |  |  |  |  |  |
|  |  |  | [-.25, .19] | [-.09, .33] | [.25, .60] | [.13, .52] |  |  |  |  |  |  |  |
| 6. Kama muta | 2.46 | 1.41 | .04 | .17 | .19 | .00 | .37** |  |  |  |  |  |  |
|  |  |  | [-.18, .26] | [-.05, .37] | [-.02, .40] | [-.22, .22] | [.16, .54] |  |  |  |  |  |  |
| 7. Admiration | 2.32 | 1.45 | -.03 | .14 | .32** | .33** | .64** | .32** |  |  |  |  |  |
|  |  |  | [-.24, .19] | [-.08, .34] | [.11, .50] | [.13, .51] | [.50, .76] | [.11, .50] |  |  |  |  |  |
| 8. Anger | 1.43 | 0.87 | -.05 | .16 | -.17 | -.01 | .19 | .42** | .25* |  |  |  |  |
|  |  |  | [-.27, .16] | [-.06, .36] | [-.37, .05] | [-.22, .21] | [-.03, .39] | [.22, .58] | [.03, .44] |  |  |  |  |
| 9. Approach | 3.57 | 1.40 | .01 | -.09 | .47** | .45** | .57** | .27* | .46** | -.02 |  |  |  |
|  |  |  | [-.21, .23] | [-.30, .13] | [.29, .63] | [.26, .61] | [.41, .70] | [.05, .46] | [.27, .61] | [-.23, .20] |  |  |  |
| 10. Avoidance | 1.98 | 1.05 | -.00 | .05 | -.32** | -.25* | -.21 | .02 | -.21 | .22 | -.31** |  |  |
|  |  |  | [-.22, .21] | [-.17, .26] | [-.50, -.11] | [-.44, -.03] | [-.41, .01] | [-.20, .24] | [-.41, .01] | [-.00, .41] | [-.49, -.10] |  |  |
| 11. Donation | 2.86 | 1.57 | .12 | -.04 | .13 | .07 | .16 | .27* | .21 | .04 | .33** | .08 |  |
|  |  |  | [-.11, .33] | [-.26, .18] | [-.09, .34] | [-.15, .29] | [-.06, .37] | [.05, .46] | [-.01, .41] | [-.18, .26] | [.12, .52] | [-.15, .29] |  |
| 12. Volunteering | 3.99 | 1.81 | .23* | .05 | .17 | .16 | .30** | .42** | .30** | .21 | .29** | -.20 | .40** |
|  |  |  | [.01, .43] | [-.17, .27] | [-.06, .38] | [-.06, .37] | [.09, .49] | [.22, .58] | [.09, .49] | [-.01, .41] | [.08, .48] | [-.40, .02] | [.20, .57] |

*Note.* Approach = Approach intentions; Avoidance = Avoidance intentions; Donation = Donation intentions; Volunteering = Volunteering intentions*. M* and *SD* are used to represent mean and standard deviation, respectively. Gender is coded 1 for women and 2 for men Values in square brackets indicate the 95% confidence interval for each correlation. * indicates *p* < .05. ** indicates *p* < .01.

**Supplementary Table S3.3**

*Descriptive Statistics and Correlation Analyses with Main Variables (Study 3, Group membership = Outgroup).*

| Variable | *M* | *SD* | 1 | 2 | 3 | 4 | 5 | 6 | 7 | 8 | 9 | 10 | 11 |
| --- | --- | --- | --- | --- | --- | --- | --- | --- | --- | --- | --- | --- | --- |
|  |  |  |  |  |  |  |  |  |  |  |  |  |  |
| 1. Gender | - | - |  |  |  |  |  |  |  |  |  |  |  |
|  |  |  |  |  |  |  |  |  |  |  |  |  |  |
| 2. Age | 23.89 | 8.59 | -.19 |  |  |  |  |  |  |  |  |  |  |
|  |  |  | [-.38, .02] |  |  |  |  |  |  |  |  |  |  |
| 3. Warmth | 4.47 | 1.48 | -.22* | .07 |  |  |  |  |  |  |  |  |  |
|  |  |  | [-.41, -.01] | [-.14, .28] |  |  |  |  |  |  |  |  |  |
| 4. Competence | 4.46 | 1.05 | .05 | .08 | .63** |  |  |  |  |  |  |  |  |
|  |  |  | [-.16, .26] | [-.14, .28] | [.49, .74] |  |  |  |  |  |  |  |  |
| 5. Liking | 3.16 | 1.54 | -.07 | .24* | .51** | .40** |  |  |  |  |  |  |  |
|  |  |  | [-.27, .14] | [.03, .43] | [.33, .65] | [.20, .56] |  |  |  |  |  |  |  |
| 6. Kama muta | 2.60 | 1.56 | .00 | .30** | .43** | .31** | .66** |  |  |  |  |  |  |
|  |  |  | [-.21, .22] | [.09, .48] | [.25, .59] | [.10, .49] | [.52, .76] |  |  |  |  |  |  |
| 7. Admiration | 3.10 | 1.99 | -.10 | .00 | .52** | .38** | .56** | .45** |  |  |  |  |  |
|  |  |  | [-.31, .11] | [-.21, .21] | [.35, .66] | [.19, .55] | [.39, .69] | [.27, .60] |  |  |  |  |  |
| 8. Anger | 1.42 | 0.88 | .07 | .02 | -.23* | -.24* | .03 | .18 | -.02 |  |  |  |  |
|  |  |  | [-.14, .27] | [-.19, .23] | [-.42, -.02] | [-.43, -.04] | [-.18, .24] | [-.03, .38] | [-.23, .19] |  |  |  |  |
| 9. Approach | 4.33 | 1.58 | -.05 | -.04 | .58** | .40** | .52** | .38** | .44** | -.04 |  |  |  |
|  |  |  | [-.26, .16] | [-.25, .17] | [.42, .70] | [.20, .56] | [.35, .66] | [.19, .55] | [.26, .60] | [-.25, .18] |  |  |  |
| 10. Avoidance | 1.71 | 1.04 | .00 | .02 | -.35** | -.14 | -.24* | -.06 | -.13 | .31** | -.29** |  |  |
|  |  |  | [-.21, .21] | [-.19, .23] | [-.53, -.15] | [-.34, .07] | [-.43, -.03] | [-.27, .15] | [-.33, .08] | [.10, .49] | [-.47, -.09] |  |  |
| 11. Donation | 2.86 | 1.49 | -.03 | -.14 | .12 | .12 | .30** | .18 | .32** | .01 | .18 | .01 |  |
|  |  |  | [-.24, .19] | [-.35, .08] | [-.10, .32] | [-.09, .33] | [.09, .48] | [-.03, .38] | [.11, .50] | [-.21, .22] | [-.04, .38] | [-.21, .23] |  |
| 12. Volunteering | 4.35 | 1.65 | .06 | .06 | .20 | .25* | .19 | .21 | .31** | -.08 | .18 | -.17 | .11 |
|  |  |  | [-.16, .27] | [-.16, .27] | [-.02, .40] | [.04, .44] | [-.03, .39] | [-.01, .41] | [.10, .50] | [-.29, .13] | [-.03, .38] | [-.37, .05] | [-.11, .32] |

*Note.* Approach = Approach intentions; Avoidance = Avoidance intentions; Donation = Donation intentions; Volunteering = Volunteering intentions*. M* and *SD* are used to represent mean and standard deviation, respectively. Gender is coded 1 for women and 2 for men Values in square brackets indicate the 95% confidence interval for each correlation. * indicates *p* < .05. ** indicates *p* < .01.

**Supplementary Table S3.4**

*MG−Path Analyses of the Effects of Smiling on Approach and Avoidance Intentions via Inferences of Warmth and Competence (Study 3).*

| Effect | Group = Ingroup | | | | |  |  | Group = Outgroup | | | | |  |
| --- | --- | --- | --- | --- | --- | --- | --- | --- | --- | --- | --- | --- | --- |
|  | *B* | *SE* | *p* | 95% CI | | β |  | *B* | *SE* | *p* | 95% CI | | β |
|  |  |  |  | *LL* | *UL* |  |  |  |  |  | *LL* | *UL* |  |
| *Approach intentions* | | | | | | | | | | | | | |
| Total effect | 0.346 | 0.307 | .260 | -0.256 | 0.947 | .123 |  | 1.099 | 0.317 | .001 | 0.479 | 1.720 | .349 |
| Direct effects |  |  |  |  |  |  |  |  |  |  |  |  |  |
| Condition on approach | -1.062 | 0.357 | .003 | -1.762 | -0.363 | -.379 |  | -0.576 | 0.415 | .165 | -1.389 | 0.236 | -.183 |
| Warmth on approach | 0.680 | 0.137 | < .001 | 0.410 | 0.949 | .660 |  | 0.754 | 0.168 | < .001 | 0.424 | 1.084 | .703 |
| Competence on approach | 0.449 | 0.147 | .002 | 0.160 | 0.737 | .282 |  | 0.030 | 0.171 | .860 | -0.306 | 0.366 | .020 |
| Condition on warmth | 1.969 | 0.207 | < .001 | 1.562 | 2.375 | .724 |  | 2.191 | 0.210 | < .001 | 1.780 | 2.603 | .746 |
| Condition on competence | 0.155 | 0.194 | .423 | -0.224 | 0.535 | .088 |  | 0.798 | 0.207 | < .001 | 0.394 | 1.203 | .383 |
| Indirect Effects via |  |  |  |  |  |  |  |  |  |  |  |  |  |
| Warmth | 1.338 | 0.305 | < .001 | 0.740 | 1.936 | .478 |  | 1.652 | 0.402 | < .001 | 0.865 | 2.439 | .524 |
| Competence | 0.070 | 0.090 | .438 | -0.106 | 0.246 | .025 |  | 0.024 | 0.137 | .861 | -0.244 | 0.292 | .008 |
| *Avoidance intentions* | | | | | | | | | | | | | |
| Total effect | -0.522 | 0.225 | .020 | -0.963 | -0.081 | -.248 |  | -0.576 | 0.214 | .007 | -0.995 | -0.157 | -.278 |
| Direct effects |  |  |  |  |  |  |  |  |  |  |  |  |  |
| Condition on avoidance | -0.168 | 0.321 | .601 | -0.796 | 0.461 | -.080 |  | -0.006 | 0.315 | .986 | -0.622 | 0.611 | -.003 |
| Warmth on avoidance | -0.163 | 0.123 | .186 | -0.405 | 0.079 | -.211 |  | -0.312 | 0.128 | .015 | -0.562 | -0.061 | -.441 |
| Competence on avoidance | -0.211 | 0.132 | .109 | -0.470 | 0.047 | -.177 |  | 0.141 | 0.130 | .279 | -0.114 | 0.395 | .141 |
| Indirect effects via |  |  |  |  |  |  |  |  |  |  |  |  |  |
| Warmth | -0.321 | 0.245 | .190 | -0.802 | 0.159 | -.153 |  | -0.683 | 0.288 | .018 | -1.247 | -0.119 | -.329 |
| Competence | -0.033 | 0.046 | .473 | -0.123 | 0.057 | -.016 |  | 0.112 | 0.108 | .297 | -0.099 | 0.324 | .054 |
| *Model fit* | | | | | | | | | | | | | |
| *CFI* | 1.000 | | | | | | | | | | | | |
| *TLI* | 1.000 | | | | | | | | | | | | |
| *RMSEA* (90% CI) | 0.000 (0.000, 0.000) | | | | | | | | | | | | |
| *SRMR* | 0.000 | | | | | | | | | | | | |

*Note.* Condition = Neutral vs. Smiling Display; Approach = Approach intentions; Avoidance = Avoidance intentions; *B* = Non−standardized estimates; *SE* = Standard error; β = Standardized coefficients of the model; *CFI* = Comparative Fit Index; *TLI* = Tucker−Lewis Index; *RMSEA* = Root Mean Square Error of Approximation; *SRMR* = Standardized Root Mean Square Residual.

In the case of the ingroup member, smiling had indirect effects on approach intentions via higher perceptions of warmth. In the case of the outgroup member (immigrant), smiling had similar indirect effects on approach and avoidance via warmth.

When we evaluated the unconstraint model (*CFI* = .982, *TLI* = .954, *RMSEA* = .086, 90%*CI* [.00, .17], *SRMR* = .066), we observed a highly similar pattern; smiling had effects on approach and avoidance intentions via warmth, and on approach intentions via perceptions of competence (see R code in SOM).

**Supplementary Table S3.5**

*MG−Path Analyses of the Effects of Smiling on Donation and Volunteering Intentions via Inferences of Warmth and Competence (Study 3).*

| Effect | Group = Ingroup | | | | |  |  | Group = Outgroup | | | | |  |
| --- | --- | --- | --- | --- | --- | --- | --- | --- | --- | --- | --- | --- | --- |
|  | *B* | *SE* | *p* | 95% CI | | β |  | *B* | *SE* | *p* | 95% CI | | β |
|  |  |  |  | *LL* | *UL* |  |  |  |  |  | *LL* | *UL* |  |
| *Donation intentions* | | | | | | | | | | | | | |
| Total effect | -0.152 | 0.352 | .665 | -0.843 | 0.538 | -.049 |  | 0.219 | 0.324 | .499 | -0.416 | 0.854 | .074 |
| Direct effects |  |  |  |  |  |  |  |  |  |  |  |  |  |
| Condition on donation | -0.945 | 0.504 | .061 | -1.933 | 0.043 | -.302 |  | -0.023 | 0.490 | .963 | -0.982 | 0.937 | -.008 |
| Warmth on donation | 0.404 | 0.194 | .037 | 0.024 | 0.783 | .352 |  | 0.070 | 0.197 | .722 | -0.315 | 0.455 | .070 |
| Competence on donation | -0.019 | 0.209 | .926 | -0.429 | 0.390 | -.011 |  | 0.119 | 0.202 | .556 | -0.276 | 0.514 | .083 |
| Condition on warmth | 1.973 | 0.213 | < .001 | 1.555 | 2.390 | .721 |  | 2.200 | 0.218 | < .001 | 1.772 | 2.628 | .742 |
| Condition on competence | 0.179 | 0.198 | .365 | -0.209 | 0.566 | .101 |  | 0.743 | 0.213 | < .001 | 0.326 | 1.160 | .358 |
| Indirect effects via |  |  |  |  |  |  |  |  |  |  |  |  |  |
| Warmth | 0.796 | 0.392 | .042 | 0.028 | 1.564 | .254 |  | 0.154 | 0.433 | .723 | -0.695 | 1.002 | .052 |
| Competence | -0.003 | 0.038 | .927 | -0.077 | 0.070 | -.001 |  | 0.088 | 0.152 | .561 | -0.210 | 0.386 | .030 |
| *Volunteering intentions* | | | | | | | | | | | | | |
| Total effect | 0.050 | 0.406 | .903 | -0.747 | 0.846 | .014 |  | 0.894 | 0.346 | .010 | 0.217 | 1.572 | .273 |
| Direct effects |  |  |  |  |  |  |  |  |  |  |  |  |  |
| Condition on volunteering | -0.720 | 0.581 | .216 | -1.859 | 0.420 | -.199 |  | 1.128 | 0.513 | .028 | 0.122 | 2.133 | .344 |
| Warmth on volunteering | 0.373 | 0.224 | .095 | -0.065 | 0.811 | .283 |  | -0.251 | 0.206 | .224 | -0.654 | 0.153 | -.227 |
| Competence on volunteering | 0.184 | 0.241 | .446 | -0.288 | 0.656 | .090 |  | 0.428 | 0.211 | .043 | 0.014 | 0.842 | .271 |
| Indirect effects via |  |  |  |  |  |  |  |  |  |  |  |  |  |
| Warmth | 0.736 | 0.448 | .100 | -0.142 | 1.614 | .204 |  | -0.551 | 0.456 | .227 | -1.446 | 0.343 | -.168 |
| Competence | 0.033 | 0.056 | .560 | -0.078 | 0.143 | .009 |  | 0.318 | 0.181 | .080 | -0.038 | 0.674 | .097 |
| *Model fit* | | | | | | | | | | | | | |
| *CFI* | 1.000 | | | | | | | | | | | | |
| *TLI* | 1.000 | | | | | | | | | | | | |
| *RMSEA* (90% *CI*) | 0.000 (0.000, 0.000) | | | | | | | | | | | | |
| *SRMR* | 0.000 | | | | | | | | | | | | |

*Note.* Condition = Neutral vs. Smiling Display; Donation = Donation intentions; Volunteering = Volunteering intentions; *B* = Non−standardized estimates; *SE* = Standard error; β = Standardized coefficients of the model; *CFI* = Comparative Fit Index; *TLI* = Tucker−Lewis Index; *RMSEA* = Root Mean Square Error of Approximation; *SRMR* = Standardized Root Mean Square Residual.

We found indirect effects of smiling on donation intentions via warmth when the target presented as a member of the ingroup. In the case of the outgroup, conversely, we found only a marginally significant effect of smiling on volunteering via perceptions of competence (*p* = .080).

When replicating these effects using a fully constraint model (*CFI* = .989, *TLI* = .972, *RMSEA* = .058, 90%*CI* [.00, .15], *SRMR* = .058), we found only marginally significant effects of smiling on volunteering via increased perceptions of competence (*p* = .090).

**Supplementary Table S3.6**

*MG−Path Analyses of the Effects of Smiling on Approach and Avoidance Intentions via Host Culture Member’ Affective Reactions (Study 3).*

| Effect | Group = Ingroup | | | | |  |  | Group = Outgroup | | | | |  |
| --- | --- | --- | --- | --- | --- | --- | --- | --- | --- | --- | --- | --- | --- |
|  | *B* | *SE* | *p* | 95% CI | | β |  | *B* | *SE* | *p* | 95% CI | | β |
|  |  |  |  | *LL* | *UL* |  |  |  |  |  | *LL* | *UL* |  |
| *Approach intentions* | | | | | | | | | | | | | |
| Total effect | 0.346 | 0.307 | .260 | -0.256 | 0.947 | .123 |  | 1.099 | 0.317 | .001 | 0.479 | 1.720 | .349 |
| Direct effects |  |  |  |  |  |  |  |  |  |  |  |  |  |
| Condition on approach | -0.154 | 0.263 | .559 | -0.670 | 0.362 | -.055 |  | 0.455 | 0.329 | .166 | -0.189 | 1.099 | .144 |
| Liking on approach | 0.510 | 0.130 | < .001 | 0.255 | 0.766 | .471 |  | 0.371 | 0.131 | .005 | 0.114 | 0.627 | .360 |
| Kama muta on approach | 0.121 | 0.101 | .232 | -0.077 | 0.319 | .121 |  | 0.019 | 0.125 | .877 | -0.226 | 0.265 | .019 |
| Admiration on approach | 0.169 | 0.112 | .132 | -0.051 | 0.389 | .174 |  | 0.140 | 0.088 | .110 | -0.032 | 0.313 | .177 |
| Anger on approach | -0.341 | 0.161 | .034 | -0.656 | -0.025 | -.210 |  | -0.017 | 0.173 | .921 | -0.356 | 0.322 | -.010 |
| Condition on liking | 0.636 | 0.277 | .022 | 0.094 | 1.178 | .246 |  | 1.056 | 0.308 | .001 | 0.453 | 1.660 | .345 |
| Condition on kama muta | -0.204 | 0.311 | .511 | -0.813 | 0.404 | -.072 |  | 1.027 | 0.314 | .001 | 0.412 | 1.642 | .331 |
| Condition on admiration | 0.407 | 0.316 | .198 | -0.212 | 1.027 | .141 |  | 1.603 | 0.390 | < .001 | 0.840 | 2.367 | .404 |
| Condition on anger | -0.384 | 0.186 | .039 | -0.749 | -0.019 | -.222 |  | -0.474 | 0.181 | .009 | -0.829 | -0.120 | -.271 |
| Indirect effects via |  |  |  |  |  |  |  |  |  |  |  |  |  |
| Liking | 0.324 | 0.164 | .047 | 0.004 | 0.645 | .116 |  | 0.391 | 0.179 | .029 | 0.040 | 0.743 | .124 |
| Kama muta | -0.025 | 0.043 | .565 | -0.108 | 0.059 | -.009 |  | 0.020 | 0.129 | .878 | -0.232 | 0.272 | .006 |
| Admiration | 0.069 | 0.070 | .327 | -0.069 | 0.206 | .025 |  | 0.225 | 0.151 | .137 | -0.071 | 0.521 | .071 |
| Anger | 0.131 | 0.089 | .140 | -0.043 | 0.304 | .047 |  | 0.008 | 0.082 | .921 | -0.153 | 0.169 | .003 |
| *Avoidance intentions* | | | | | | | | | | | | | |
| Total effect | -0.522 | 0.225 | .020 | -0.963 | -0.081 | -.248 |  | -0.576 | 0.214 | .007 | -0.995 | -0.157 | -.278 |
| Direct effects |  |  |  |  |  |  |  |  |  |  |  |  |  |
| Condition on avoidance | -0.296 | 0.231 | .201 | -0.749 | 0.158 | -.140 |  | -0.346 | 0.238 | .146 | -0.812 | 0.121 | -.167 |
| Liking on avoidance | -0.086 | 0.114 | .452 | -0.311 | 0.138 | -.106 |  | -0.206 | 0.095 | .030 | -0.391 | -0.020 | -.304 |
| Kama muta on avoidance | 0.003 | 0.089 | .969 | -0.170 | 0.177 | .005 |  | 0.083 | 0.091 | .360 | -0.095 | 0.260 | .124 |
| Admiration on avoidance | -0.134 | 0.099 | .174 | -0.327 | 0.059 | -.184 |  | 0.027 | 0.064 | .671 | -0.098 | 0.152 | .052 |
| Anger on avoidance | 0.303 | 0.142 | .032 | 0.025 | 0.580 | .249 |  | 0.298 | 0.125 | .017 | 0.053 | 0.543 | .252 |
| Indirect effects via |  |  |  |  |  |  |  |  |  |  |  |  |  |
| Liking | -0.055 | 0.077 | .474 | -0.205 | 0.095 | -.026 |  | -0.217 | 0.118 | .066 | -0.449 | 0.015 | -.105 |
| Kama muta | -0.001 | 0.018 | .969 | -0.036 | 0.035 | < .001 |  | 0.085 | 0.097 | .378 | -0.104 | 0.274 | .041 |
| Admiration | -0.055 | 0.058 | .350 | -0.169 | 0.060 | -.026 |  | 0.043 | 0.103 | .673 | -0.158 | 0.244 | .021 |
| Anger | -0.116 | 0.078 | .138 | -0.270 | 0.037 | -.055 |  | -0.141 | 0.080 | .078 | -0.298 | 0.016 | -.068 |
| *Model fit* | | | | | | | | | | | | | |
| *CFI* | 1.000 | | | | | | | | | | | | |
| *TLI* | 1.000 | | | | | | | | | | | | |
| *RMSEA* (90% *CI*) | 0.000 (0.000, 0.000) | | | | | | | | | | | | |
| *SRMR* | 0.000 | | | | | | | | | | | | |

*Note.* Condition = Neutral vs. Smiling Display; Approach = Approach intentions; Avoidance = Avoidance intentions; *B* = Non−standardized estimates; *SE* = Standard error; β = Standardized coefficients of the model; *CFI* = Comparative Fit Index; *TLI* = Tucker−Lewis Index; *RMSEA* = Root Mean Square Error of Approximation; *SRMR* = Standardized Root Mean Square Residual.

There were significant indirect effects of smiling on approach intentions via liking both in response to an ingroup and outgroup member. We also observed a marginally significant effects via anger on avoidance intentions (*p* = .078), but only in the case of the outgroup member.

The same theoretical model when tested constrained (*CFI* = .977, *TLI* = .931, *RMSEA* = .072, 90%*CI* [.00, .14], *SRMR* = .062) revealed indirect effects of smiling on approaching and avoidance. Additionally, we found an indirect effect via anger on avoidance and a marginally significant effect via admiration on approaching (*p* = .061).

**Supplementary Table S3.7**

*MG−Path Analyses of the Effects of Smiling on Donation and Volunteering Intentions via Host Culture Members’ Affective Reactions (Study 3).*

| Effect | Group = Ingroup | | | | |  |  | Group = Outgroup | | | | |  |
| --- | --- | --- | --- | --- | --- | --- | --- | --- | --- | --- | --- | --- | --- |
|  | *B* | *SE* | *p* | 95% CI | | β |  | *B* | *SE* | *p* | 95% CI | | β |
|  |  |  |  | *LL* | *UL* |  |  |  |  |  | *LL* | *UL* |  |
| *Donation intentions* | | | | | | | | | | | | | |
| Total effect | -0.152 | 0.352 | .665 | -0.843 | 0.538 | -.049 |  | 0.219 | 0.324 | .499 | -0.416 | 0.854 | .074 |
| Direct effects |  |  |  |  |  |  |  |  |  |  |  |  |  |
| Condition on donation | -0.140 | 0.359 | .697 | -0.843 | 0.564 | -.045 |  | -0.112 | 0.352 | .750 | -0.802 | 0.578 | -.038 |
| Liking on donation | -0.038 | 0.162 | .813 | -0.356 | 0.279 | -.032 |  | 0.259 | 0.144 | .072 | -0.023 | 0.542 | .268 |
| Kama muta on donation | 0.296 | 0.135 | .028 | 0.032 | 0.561 | .269 |  | -0.013 | 0.140 | .928 | -0.288 | 0.262 | -.013 |
| Admiration on donation | 0.306 | 0.146 | .036 | 0.020 | 0.591 | .257 |  | 0.101 | 0.117 | .387 | -0.128 | 0.330 | .105 |
| Anger on donation | -0.217 | 0.213 | .310 | -0.635 | 0.201 | -.121 |  | 0.004 | 0.198 | .984 | -0.383 | 0.391 | .002 |
| Condition on liking | 0.632 | 0.284 | .026 | 0.076 | 1.189 | .243 |  | 1.013 | 0.317 | .001 | 0.392 | 1.634 | .331 |
| Condition on kama muta | -0.134 | 0.319 | .674 | -0.760 | 0.491 | -.047 |  | 1.005 | 0.315 | .001 | 0.388 | 1.622 | .331 |
| Condition on admiration | -0.120 | 0.297 | .687 | -0.701 | 0.462 | -.045 |  | 0.822 | 0.325 | .011 | 0.186 | 1.459 | .268 |
| Condition on anger | -0.406 | 0.192 | .034 | -0.782 | -0.030 | -.232 |  | -0.437 | 0.180 | .015 | -0.790 | -0.085 | -.258 |
| Indirect effects via |  |  |  |  |  |  |  |  |  |  |  |  |  |
| Liking | -0.024 | 0.103 | .814 | -0.226 | 0.178 | -.008 |  | 0.262 | 0.168 | .117 | -0.066 | 0.591 | .089 |
| Kama muta | -0.040 | 0.096 | .679 | -0.229 | 0.149 | -.013 |  | -0.013 | 0.141 | .928 | -0.289 | 0.264 | -.004 |
| Admiration | -0.037 | 0.092 | .692 | -0.218 | 0.144 | -.012 |  | 0.083 | 0.102 | .413 | -0.116 | 0.282 | .028 |
| Anger | 0.088 | 0.096 | .360 | -0.100 | 0.276 | .028 |  | -0.002 | 0.086 | .984 | -0.171 | 0.168 | -.001 |
| *Volunteering intentions* | | | | | | | | | | | | | |
| Total effect | 0.050 | 0.406 | .903 | -0.747 | 0.846 | .014 |  | 0.894 | 0.346 | .010 | 0.217 | 1.572 | .273 |
| Direct effects |  |  |  |  |  |  |  |  |  |  |  |  |  |
| Condition on volunteering | 0.130 | 0.389 | .738 | -0.632 | 0.893 | .036 |  | 0.594 | 0.380 | .118 | -0.150 | 1.338 | .181 |
| Liking on volunteering | 0.066 | 0.176 | .705 | -0.278 | 0.411 | .048 |  | -0.072 | 0.155 | .644 | -0.377 | 0.233 | -.067 |
| Kama Muta on volunteering | 0.424 | 0.147 | .004 | 0.137 | 0.712 | .333 |  | 0.119 | 0.151 | .431 | -0.177 | 0.416 | .111 |
| Admiration on volunteering | 0.313 | 0.158 | .047 | 0.004 | 0.623 | .229 |  | 0.272 | 0.126 | .031 | 0.025 | 0.519 | .255 |
| Anger on volunteering | 0.070 | 0.231 | .763 | -0.383 | 0.523 | .034 |  | -0.069 | 0.213 | .747 | -0.486 | 0.349 | -.036 |
| Indirect effects via |  |  |  |  |  |  |  |  |  |  |  |  |  |
| Liking | 0.042 | 0.113 | .709 | -0.179 | 0.263 | .012 |  | -0.073 | 0.159 | .647 | -0.385 | 0.239 | -.022 |
| Kama muta | -0.057 | 0.137 | .677 | -0.325 | 0.211 | -.016 |  | 0.120 | 0.157 | .445 | -0.187 | 0.427 | .037 |
| Admiration | -0.037 | 0.095 | .693 | -0.223 | 0.148 | -.010 |  | 0.224 | 0.136 | .101 | -0.043 | 0.490 | .068 |
| Anger | -0.028 | 0.095 | .765 | -0.214 | 0.157 | -.008 |  | 0.030 | 0.094 | .749 | -0.154 | 0.214 | .009 |
| *Model fit* | | | | | | | | | | | | | |
| *CFI* | 1.000 | | | | | | | | | | | | |
| *TLI* | 1.000 | | | | | | | | | | | | |
| *RMSEA* (90% *CI*) | 0.000 (0.000, 0.000) | | | | | | | | | | | | |
| *SRMR* | 0.000 | | | | | | | | | | | | |

*Note.* Condition = Neutral vs. Smiling Display; Donation = Donation intentions; Volunteering = Volunteering intentions; *B* = Non−standardized estimates; *SE* = Standard error; β = Standardized coefficients of the model; *CFI* = Comparative Fit Index. *TLI* = Tucker−Lewis Index; *RMSEA* = Root Mean Square Error of Approximation; *SRMR* = Standardized Root Mean Square Residual.

There were no indirect effects of smiling on donation or volunteering intentions via host culture members’ affective reactions, and neither when testing the constraint model (*CFI* = .959, *TLI* = .876, *RMSEA* = .081, 90%*CI* [.00, .15], *SRMR* = .068).

**Supplementary Table S3.8**

*MG−Path Analyses of the Effects of Smiling on Kama Muta and Admiration via Warmth and Competence (Study 3).*

|  | Group = Ingroup | | | | |  |  | Group = Outgroup | | | | |  |
| --- | --- | --- | --- | --- | --- | --- | --- | --- | --- | --- | --- | --- | --- |
| Effect | *B* | *SE* | *p* | 95% CI | | β |  | *B* | *SE* | *p* | 95% CI | | β |
|  |  |  |  | *LL* | *UL* |  |  |  |  |  | *LL* | *UL* |  |
| *Kama muta* | | | | | | | | | | | | | |
| Total effect | -0.204 | 0.305 | .503 | -0.802 | 0.393 | -.074 |  | 1.027 | 0.309 | .001 | 0.421 | 1.633 | .335 |
| Direct effects |  |  |  |  |  |  |  |  |  |  |  |  |  |
| Condition on warmth | 1.969 | 0.207 | < .001 | 1.562 | 2.375 | .724 |  | 2.191 | 0.210 | < .001 | 1.780 | 2.603 | .746 |
| Condition on kama muta | -1.084 | 0.411 | .008 | -1.890 | -0.278 | -.392 |  | 0.257 | 0.443 | .561 | -0.611 | 1.126 | .084 |
| Warmth on kama muta | 0.447 | 0.148 | .002 | 0.157 | 0.737 | .439 |  | 0.351 | 0.149 | .018 | 0.059 | 0.643 | .337 |
| Indirect Effects via |  |  |  |  |  |  |  |  |  |  |  |  |  |
| Warmth | 0.880 | 0.305 | .004 | 0.281 | 1.479 | .318 |  | 0.769 | 0.334 | .021 | 0.114 | 1.425 | .251 |
| *Admiration* | | | | | | | | | | | | | |
| Total effect | 0.407 | 0.320 | .203 | -0.219 | 1.034 | .139 |  | 1.603 | 0.388 | < .001 | 0.844 | 2.363 | .405 |
| Direct effects |  |  |  |  |  |  |  |  |  |  |  |  |  |
| Condition on competence | 0.155 | 0.194 | .423 | -0.224 | 0.535 | .088 |  | 0.798 | 0.207 | < .001 | 0.394 | 1.203 | .383 |
| Condition on admiration | 0.317 | 0.300 | .291 | -0.272 | 0.905 | .108 |  | 1.228 | 0.404 | .002 | 0.435 | 2.021 | .311 |
| Competence on admiration | 0.583 | 0.163 | < .001 | 0.263 | 0.902 | .351 |  | 0.470 | 0.189 | .013 | 0.100 | 0.841 | .248 |
| Indirect Effects via |  |  |  |  |  |  |  |  |  |  |  |  |  |
| Competence | 0.091 | 0.116 | .434 | -0.136 | 0.317 | .031 |  | 0.375 | 0.179 | .036 | 0.024 | 0.727 | .095 |
| *Model fit* | | | | | | | | | | | | | |
| *CFI* | .968 | | | | | | | | | | | | |
| *TLI* | .839 | | | | | | | | | | | | |
| *RMSEA* (90% CI) | .158 (.63, 1.26) | | | | | | | | | | | | |
| *SRMR* | .039 | | | | | | | | | | | | |

*Note.* Condition = Neutral vs. Smiling Display; Approach = Approach intentions; Avoidance = Avoidance intentions; *B* = Non−standardized estimates; *SE* = Standard error; β = Standardized coefficients of the model; *CFI* = Comparative Fit Index; *TLI* = Tucker−Lewis Index; *RMSEA* = Root Mean Square Error of Approximation; *SRMR* = Standardized Root Mean Square Residual.

The analysis of the multiple and sequential mediation analysis revealed that there are indirect effects of smiling on kama muta via perceived warmth (ingroup & outgroup: β = .32**/.25*) and on admiration via perceived competence (only outgroup: β = .03/.09*). Additionally, warmth and competence always predict kama muta and admiration, respectively.

**Supplementary Figure S3.1**

*Multi-group Parallel and Sequential Mediation Model of the Effects of Smiling through Cognitive Inferences and Emotions (Study 3).*


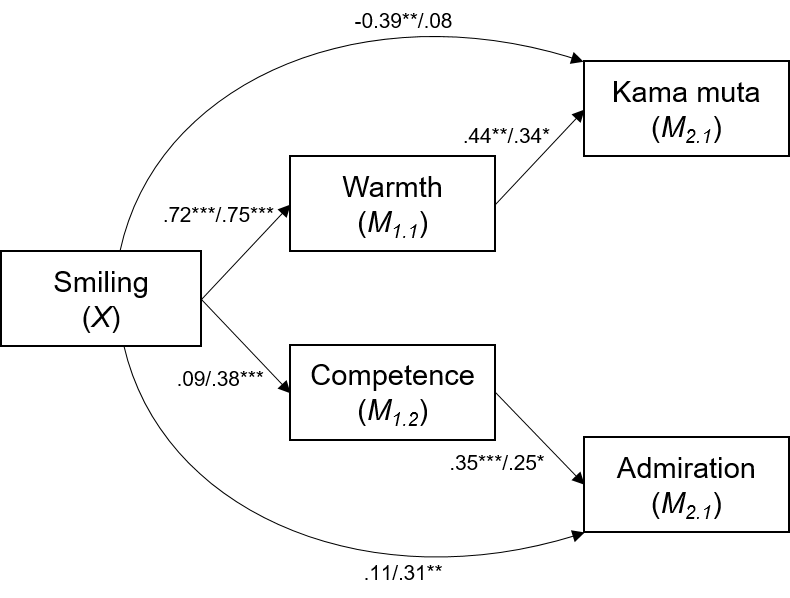


*Note*. All estimates are standardized regression coefficients; the values on the left and the right indicates when the group manipulation was Ingroup (*n* = 82) and Outgroup (*n* = 87), respectively. *, **, and *** indicate *p* values of < .05, < .01, and < .001, respectively.

# Section IV Study 4: Stimuli Validation, Descriptive Data, Bivariate Correlations, and MG-Path Analyses

Section IV presents complementary analyses for Study 4, following the logic and expanding those in Study 3. These include a stimuli validation study to select the experimental stimuli, descriptive analyses and bivariate correlations (Supplementary Table S4.1, Supplementary Table S4.2, and Supplementary Table S4.3 for the total sample, Ingroup condition and Outgroup condition, respectively). Then, we conducted 2 (stimulus) X 4 (country) ANOVA analyses to expand those presented in the article (Supplementary Table S4.4). We also conducted path analyses examining the effects of smiling on approach and avoidance intentions via cognitive (Supplementary Table S4.5) and affective mechanisms (Supplementary Table S4.6), and the effects of smiling on donation and volunteering intentions via cognitive (Supplementary Table S4.7) and affective mechanisms (Supplementary Table S4.8).

The path analyses here are a pre-registered test replicating the model from Study 3, testing simultaneously the effects of smiling on multiple mediators and behavioral intention variables. We conducted the analyses with the condition (neutral display coded as 0, smiling as 1) as the independent variable, and approach and avoidance as dependent ones. We used bootstrapping was used to calculate confidence intervals. We used immigrant (outgroup member) vs. host culture member (ingroup member) factor as the grouping factor to simultaneously test predicted effects, comparing a constrained versus an unconstrained model (before the final ones). We found significant differences among the models (χ^2^(8, *N* = 1629) = 25.079, *p* = .001) and decided to report the results of the unconstrained one, where all regression paths were freely estimated across both group categories, due to its better fit indexes.

Full details are available in the R code on the OSF project website (<https://osf.io/9gh5w/>).

**Stimuli Validation Study**

To validate the images to be used in Study 4, 300 participants (175 women, 116, men and 2 non-binary) aged from 18 to 89 years old (*M* = 50.9, *SD* = 14.7) evaluated the faces (all of them with neutral expression) of 40 individuals and indicated the likelihood they were from 4 different countries: Morocco, Ecuador, Spain and Romania. In this intra-person design study, participants read the following statement together with each face:

*The person of the photograph could be from [name of the country]*

For each country, we used 10 different neutral faces extracted from the Chicago Face Database (Ma et al., 2015). For Morocco, we used Middle East faces; for Ecuador, the Latin faces; and for Spain and Romania, white faces. While each participants evaluated each of the 40 faces (10 for each country), we randomly assigned the country, first, and then, presented the 10 pictures in a random order. With the evaluation of the pictures, we selected the 4 with the highest evaluation for each country which were then used as stimuli for Study 4.

**Supplementary Table S4.0**

*Evaluation of Faces and Comparison with the Mid-Point of the Scale (i.e., 3).*

|  | Descriptives | | | | |  | Comparison | |
| --- | --- | --- | --- | --- | --- | --- | --- | --- |
|  | *M* | *SD* | Median | Min. | Max. |  | *t*(297) | *p* |
| *Middle East faces* | | | | | | | | |
| Morocco2 | 3.54 | 0.91 | 4 | 1 | 5 |  | 10.396 | < .001 |
| Morocco7 | 3.10 | 1.00 | 3 | 1 | 5 |  | 2.939 | .002 |
| Morocco9 | 3.03 | 1.01 | 3 | 1 | 5 |  | 2.748 | .003 |
| Morocco1 | 2.86 | 0.94 | 3 | 1 | 5 |  | -0.061 | .524 |
| Morocco6 | 2.85 | 1.08 | 3 | 1 | 5 |  | -0.940 | .826 |
| Morocco3 | 2.80 | 0.99 | 3 | 1 | 5 |  | -1.068 | .857 |
| Morocco10 | 2.64 | 0.98 | 3 | 1 | 5 |  | -3.640 | < .001 |
| Morocco8 | 2.53 | 0.95 | 2 | 1 | 5 |  | -5.400 | < .001 |
| Morocco4 | 2.39 | 0.94 | 2 | 1 | 5 |  | -7.047 | < .001 |
| Morocco5 | 2.32 | 0.92 | 2 | 1 | 5 |  | -7.944 | < .001 |
| *Latin faces* | | | | | | | | |
| Ecuador5 | 3.75 | 0.93 | 4 | 1 | 5 |  | 14.823 | < .001 |
| Ecuador3 | 3.65 | 1.01 | 4 | 1 | 5 |  | 12.245 | < .001 |
| Ecuador8 | 3.61 | 0.95 | 4 | 1 | 5 |  | 12.922 | < .001 |
| Ecuador1 | 3.54 | 0.96 | 4 | 1 | 5 |  | 11.569 | < .001 |
| Ecuador2 | 3.43 | 1.07 | 4 | 1 | 5 |  | 9.204 | < .001 |
| Ecuador9 | 3.43 | 0.90 | 4 | 1 | 5 |  | 10.067 | < .001 |
| Ecuador6 | 3.42 | 0.94 | 4 | 1 | 5 |  | 9.306 | < .001 |
| Ecuador7 | 3.33 | 0.93 | 3 | 1 | 5 |  | 7.992 | < .001 |
| Ecuador10 | 3.07 | 1.02 | 3 | 1 | 5 |  | 3.902 | < .001 |
| Ecuador4 | 2.70 | 0.90 | 3 | 1 | 5 |  | -1.159 | .876 |
| *White faces* | | | | | | | | |
| Spain1 | 3.78 | 0.91 | 4 | 1 | 5 |  | 14.249 | < .001 |
| Spain8 | 3.53 | 0.80 | 4 | 1 | 5 |  | 11.613 | < .001 |
| Spain10 | 3.13 | 0.97 | 3 | 1 | 5 |  | 4.669 | < .001 |
| Spain6 | 3.05 | 0.99 | 3 | 1 | 5 |  | 2.908 | .002 |
| Spain5 | 3.00 | 1.08 | 3 | 1 | 5 |  | 2.450 | .007 |
| Spain4 | 2.85 | 0.96 | 3 | 1 | 5 |  | 0.953 | .171 |
| Spain9 | 2.84 | 1.02 | 3 | 1 | 5 |  | 0.281 | .389 |
| Spain7 | 2.63 | 0.98 | 2 | 1 | 5 |  | -2.567 | .995 |
| Spain2 | 2.55 | 0.98 | 2 | 1 | 5 |  | -3.604 | < .001 |
| Spain3 | 2.46 | 0.95 | 2 | 1 | 5 |  | -4.814 | < .001 |
| *White faces* | | | | | | | | |
| Romania7 | 3.32 | 0.99 | 4 | 1 | 5 |  | 7.001 | < .001 |
| Romania3 | 3.15 | 1.11 | 3 | 1 | 5 |  | 4.145 | < .001 |
| Romania5 | 3.13 | 1.00 | 3 | 1 | 5 |  | 3.788 | < .001 |
| Romania6 | 3.11 | 1.00 | 3 | 1 | 5 |  | 4.098 | < .001 |
| Romania9 | 3.07 | 0.95 | 3 | 1 | 5 |  | 2.982 | .002 |
| Romania4 | 2.94 | 1.03 | 3 | 1 | 5 |  | 1.799 | .036 |
| Romania8 | 2.94 | 0.85 | 3 | 1 | 5 |  | 1.997 | .023 |
| Romania10 | 2.93 | 0.97 | 3 | 1 | 5 |  | 1.522 | .065 |
| Romania2 | 2.73 | 0.97 | 3 | 1 | 5 |  | -1.118 | .868 |
| Romania1 | 2.58 | 0.87 | 3 | 1 | 5 |  | -3.540 | < .001 |

*Note.* The faces were retrieved from the Chicago Face Database (Ma et al., 2015) and are ordered according to the evaluations (descendent order). The contrast was conducted against the middle-point of the evaluation scale (i.e., 3); t-tests with a positive value indicate mean scores above 3.

**Supplementary Table S4.01**

*Mean Scores on Used Variables for each Face Used Across Experimental Conditions.*

|  |  | Morocco | | | |  | Ecuador | | | |  | Spain | | | |  | Romania | | | |
| --- | --- | --- | --- | --- | --- | --- | --- | --- | --- | --- | --- | --- | --- | --- | --- | --- | --- | --- | --- | --- |
| Variable | Images | Condition | *n* | *M* | *SD* |  | Condition | *n* | *M* | *SD* |  | Condition | *n* | *M* | *SD* |  | Condition | *n* | *M* | *SD* |
| Check: Group | 1 | neutral | 40 | 2.77 | 1.74 |  | neutral | 43 | 2.64 | 1.60 |  | neutral | 40 | 5.76 | 1.10 |  | neutral | 45 | 3.92 | 1.51 |
| Check: Group | 1 | smile | 42 | 2.60 | 1.50 |  | smile | 42 | 2.27 | 1.35 |  | smile | 40 | 5.62 | 1.36 |  | smile | 41 | 4.21 | 1.52 |
| Check: Group | 2 | neutral | 42 | 2.32 | 1.48 |  | neutral | 41 | 2.09 | 1.18 |  | neutral | 41 | 5.55 | 1.51 |  | neutral | 39 | 4.19 | 2.02 |
| Check: Group | 2 | smile | 44 | 1.96 | 1.22 |  | smile | 43 | 2.62 | 1.55 |  | smile | 40 | 5.66 | 1.24 |  | smile | 35 | 4.87 | 1.23 |
| Check: Group | 3 | neutral | 38 | 2.89 | 1.50 |  | neutral | 40 | 2.19 | 1.33 |  | neutral | 43 | 5.35 | 1.15 |  | neutral | 39 | 4.46 | 1.60 |
| Check: Group | 3 | smile | 37 | 3.28 | 1.65 |  | smile | 46 | 2.28 | 1.43 |  | smile | 43 | 5.66 | 1.02 |  | smile | 40 | 4.49 | 1.72 |
| Check: Group | 4 | neutral | 39 | 2.20 | 1.22 |  | neutral | 38 | 2.58 | 1.69 |  | neutral | 43 | 5.60 | 1.15 |  | neutral | 39 | 4.21 | 1.75 |
| Check: Group | 4 | smile | 42 | 2.36 | 1.25 |  | smile | 44 | 2.59 | 1.46 |  | smile | 44 | 5.66 | 1.30 |  | smile | 39 | 4.65 | 1.51 |
| Check: Group | 5 | neutral | 37 | 2.57 | 1.68 |  | neutral | 38 | 2.11 | 1.14 |  | neutral | 40 | 5.74 | 1.03 |  | neutral | 37 | 4.33 | 1.63 |
| Check: Group | 5 | smile | 39 | 2.68 | 1.78 |  | smile | 38 | 2.32 | 1.44 |  | smile | 45 | 5.92 | 1.10 |  | smile | 43 | 4.41 | 1.91 |
| Check: Neutral | 1 | neutral | 40 | 4.98 | 1.36 |  | neutral | 43 | 5.20 | 1.06 |  | neutral | 40 | 5.11 | 1.54 |  | neutral | 45 | 4.87 | 1.23 |
| Check: Neutral | 1 | smile | 42 | 2.68 | 1.26 |  | smile | 42 | 4.35 | 1.54 |  | smile | 40 | 3.06 | 1.29 |  | smile | 41 | 3.32 | 1.26 |
| Check: Neutral | 2 | neutral | 42 | 4.96 | 1.69 |  | neutral | 41 | 5.28 | 1.35 |  | neutral | 41 | 4.76 | 1.61 |  | neutral | 39 | 4.50 | 1.55 |
| Check: Neutral | 2 | smile | 44 | 2.66 | 1.26 |  | smile | 43 | 2.45 | 1.19 |  | smile | 40 | 3.58 | 1.53 |  | smile | 35 | 3.54 | 1.31 |
| Check: Neutral | 3 | neutral | 38 | 4.72 | 1.41 |  | neutral | 40 | 5.00 | 1.35 |  | neutral | 43 | 4.86 | 1.20 |  | neutral | 39 | 4.62 | 1.25 |
| Check: Neutral | 3 | smile | 37 | 2.92 | 1.30 |  | smile | 46 | 3.36 | 1.34 |  | smile | 43 | 3.08 | 1.24 |  | smile | 40 | 2.93 | 1.15 |
| Check: Neutral | 4 | neutral | 39 | 5.09 | 1.13 |  | neutral | 38 | 4.76 | 1.62 |  | neutral | 43 | 4.80 | 1.31 |  | neutral | 39 | 4.95 | 1.31 |
| Check: Neutral | 4 | smile | 42 | 2.81 | 1.29 |  | smile | 44 | 3.97 | 1.24 |  | smile | 44 | 3.02 | 1.40 |  | smile | 39 | 3.12 | 1.10 |
| Check: Neutral | 5 | neutral | 37 | 4.66 | 1.39 |  | neutral | 38 | 4.96 | 1.44 |  | neutral | 40 | 5.34 | 1.29 |  | neutral | 37 | 5.09 | 1.25 |
| Check: Neutral | 5 | smile | 39 | 3.18 | 1.02 |  | smile | 38 | 3.54 | 1.47 |  | smile | 45 | 3.97 | 1.34 |  | smile | 43 | 2.24 | 1.31 |
| Check: Smile | 1 | neutral | 40 | 3.51 | 1.12 |  | neutral | 43 | 2.63 | 1.19 |  | neutral | 40 | 3.01 | 1.35 |  | neutral | 45 | 2.67 | 1.34 |
| Check: Smile | 1 | smile | 42 | 5.56 | 0.84 |  | smile | 42 | 4.37 | 1.24 |  | smile | 40 | 4.86 | 0.95 |  | smile | 41 | 5.06 | 0.94 |
| Check: Smile | 2 | neutral | 42 | 3.02 | 1.08 |  | neutral | 41 | 2.60 | 1.34 |  | neutral | 41 | 3.59 | 1.45 |  | neutral | 39 | 2.83 | 1.26 |
| Check: Smile | 2 | smile | 44 | 5.11 | 1.20 |  | smile | 43 | 5.51 | 0.92 |  | smile | 40 | 4.86 | 1.00 |  | smile | 35 | 4.93 | 0.96 |
| Check: Smile | 3 | neutral | 38 | 2.49 | 1.25 |  | neutral | 40 | 2.69 | 1.21 |  | neutral | 43 | 3.43 | 1.32 |  | neutral | 39 | 3.21 | 1.26 |
| Check: Smile | 3 | smile | 37 | 5.16 | 1.10 |  | smile | 46 | 4.65 | 1.04 |  | smile | 43 | 5.36 | 0.93 |  | smile | 40 | 5.25 | 0.85 |
| Check: Smile | 4 | neutral | 39 | 2.35 | 1.07 |  | neutral | 38 | 2.76 | 1.25 |  | neutral | 43 | 2.35 | 0.90 |  | neutral | 39 | 3.45 | 1.25 |
| Check: Smile | 4 | smile | 42 | 5.11 | 1.28 |  | smile | 44 | 3.84 | 0.91 |  | smile | 44 | 5.48 | 1.02 |  | smile | 39 | 5.00 | 0.85 |
| Check: Smile | 5 | neutral | 37 | 3.54 | 1.40 |  | neutral | 38 | 3.36 | 1.35 |  | neutral | 40 | 2.81 | 1.36 |  | neutral | 37 | 3.31 | 1.16 |
| Check: Smile | 5 | smile | 39 | 4.74 | 1.03 |  | smile | 38 | 4.82 | 1.04 |  | smile | 45 | 4.87 | 0.92 |  | smile | 43 | 5.72 | 1.08 |
| Warmth | 1 | neutral | 40 | 4.12 | 1.20 |  | neutral | 43 | 4.14 | 1.20 |  | neutral | 40 | 4.03 | 1.05 |  | neutral | 45 | 3.99 | 1.28 |
| Warmth | 1 | smile | 42 | 5.40 | 1.05 |  | smile | 42 | 4.97 | 0.88 |  | smile | 40 | 4.98 | 1.09 |  | smile | 41 | 4.82 | 0.88 |
| Warmth | 2 | neutral | 42 | 3.84 | 1.29 |  | neutral | 41 | 4.22 | 1.30 |  | neutral | 41 | 4.23 | 1.24 |  | neutral | 39 | 3.79 | 1.27 |
| Warmth | 2 | smile | 44 | 4.50 | 1.35 |  | smile | 43 | 5.36 | 0.95 |  | smile | 40 | 5.01 | 1.04 |  | smile | 35 | 4.85 | 0.99 |
| Warmth | 3 | neutral | 38 | 3.45 | 1.61 |  | neutral | 40 | 4.00 | 1.11 |  | neutral | 43 | 3.95 | 1.29 |  | neutral | 39 | 4.09 | 0.91 |
| Warmth | 3 | smile | 37 | 4.78 | 0.72 |  | smile | 46 | 4.91 | 1.03 |  | smile | 43 | 5.29 | 0.85 |  | smile | 40 | 5.03 | 0.86 |
| Warmth | 4 | neutral | 39 | 3.69 | 1.29 |  | neutral | 38 | 3.93 | 1.18 |  | neutral | 43 | 3.48 | 1.13 |  | neutral | 39 | 4.11 | 1.03 |
| Warmth | 4 | smile | 42 | 4.96 | 0.97 |  | smile | 44 | 4.77 | 1.06 |  | smile | 44 | 5.15 | 0.99 |  | smile | 39 | 4.95 | 0.81 |
| Warmth | 5 | neutral | 37 | 4.40 | 1.05 |  | neutral | 38 | 4.29 | 1.05 |  | neutral | 40 | 3.62 | 1.03 |  | neutral | 37 | 4.23 | 1.27 |
| Warmth | 5 | smile | 39 | 4.90 | 1.14 |  | smile | 38 | 4.88 | 1.19 |  | smile | 45 | 4.75 | 1.04 |  | smile | 43 | 5.36 | 0.96 |
| Competence | 1 | neutral | 40 | 4.33 | 0.95 |  | neutral | 43 | 4.26 | 0.90 |  | neutral | 40 | 4.37 | 0.94 |  | neutral | 45 | 4.58 | 0.99 |
| Competence | 1 | smile | 42 | 4.90 | 0.97 |  | smile | 42 | 4.55 | 0.92 |  | smile | 40 | 4.71 | 1.04 |  | smile | 41 | 4.76 | 0.71 |
| Competence | 2 | neutral | 42 | 4.24 | 1.07 |  | neutral | 41 | 4.47 | 1.09 |  | neutral | 41 | 4.41 | 1.24 |  | neutral | 39 | 4.18 | 1.24 |
| Competence | 2 | smile | 44 | 4.31 | 1.18 |  | smile | 43 | 5.02 | 1.09 |  | smile | 40 | 4.94 | 1.07 |  | smile | 35 | 4.54 | 1.14 |
| Competence | 3 | neutral | 38 | 4.13 | 1.59 |  | neutral | 40 | 4.16 | 0.88 |  | neutral | 43 | 4.16 | 1.02 |  | neutral | 39 | 4.44 | 0.88 |
| Competence | 3 | smile | 37 | 4.32 | 0.65 |  | smile | 46 | 4.55 | 0.90 |  | smile | 43 | 4.84 | 0.88 |  | smile | 40 | 4.60 | 0.79 |
| Competence | 4 | neutral | 39 | 4.08 | 1.16 |  | neutral | 38 | 4.37 | 1.10 |  | neutral | 43 | 3.98 | 0.83 |  | neutral | 39 | 4.56 | 0.76 |
| Competence | 4 | smile | 42 | 4.64 | 0.93 |  | smile | 44 | 4.55 | 1.08 |  | smile | 44 | 4.79 | 0.96 |  | smile | 39 | 4.56 | 0.82 |
| Competence | 5 | neutral | 37 | 4.55 | 0.89 |  | neutral | 38 | 4.32 | 1.32 |  | neutral | 40 | 4.38 | 0.71 |  | neutral | 37 | 4.49 | 1.02 |
| Competence | 5 | smile | 39 | 4.65 | 1.17 |  | smile | 38 | 4.64 | 1.17 |  | smile | 45 | 4.52 | 1.15 |  | smile | 43 | 4.81 | 0.97 |
| Anger | 1 | neutral | 40 | 2.14 | 1.24 |  | neutral | 43 | 1.95 | 1.29 |  | neutral | 40 | 2.19 | 1.34 |  | neutral | 45 | 2.02 | 1.19 |
| Anger | 1 | smile | 42 | 1.50 | 0.96 |  | smile | 42 | 1.49 | 0.72 |  | smile | 40 | 1.35 | 0.65 |  | smile | 41 | 1.54 | 0.76 |
| Anger | 2 | neutral | 42 | 1.90 | 0.99 |  | neutral | 41 | 1.64 | 1.23 |  | neutral | 41 | 1.76 | 1.19 |  | neutral | 39 | 2.13 | 1.59 |
| Anger | 2 | smile | 44 | 2.16 | 1.75 |  | smile | 43 | 1.33 | 0.80 |  | smile | 40 | 1.52 | 0.92 |  | smile | 35 | 1.62 | 1.06 |
| Anger | 3 | neutral | 38 | 2.11 | 1.56 |  | neutral | 40 | 1.89 | 1.09 |  | neutral | 43 | 2.06 | 1.12 |  | neutral | 39 | 2.21 | 1.35 |
| Anger | 3 | smile | 37 | 2.03 | 1.33 |  | smile | 46 | 1.62 | 1.20 |  | smile | 43 | 1.52 | 0.96 |  | smile | 40 | 1.48 | 0.86 |
| Anger | 4 | neutral | 39 | 2.12 | 1.67 |  | neutral | 38 | 1.81 | 1.26 |  | neutral | 43 | 1.91 | 1.07 |  | neutral | 39 | 2.03 | 1.41 |
| Anger | 4 | smile | 42 | 1.72 | 1.15 |  | smile | 44 | 1.89 | 1.26 |  | smile | 44 | 1.36 | 0.91 |  | smile | 39 | 1.64 | 1.09 |
| Anger | 5 | neutral | 37 | 1.80 | 1.18 |  | neutral | 38 | 1.55 | 0.91 |  | neutral | 40 | 1.93 | 1.46 |  | neutral | 37 | 1.81 | 0.98 |
| Anger | 5 | smile | 39 | 1.50 | 0.93 |  | smile | 38 | 1.40 | 0.84 |  | smile | 45 | 1.56 | 0.96 |  | smile | 43 | 1.71 | 1.16 |
| Kamamuta | 1 | neutral | 40 | 2.51 | 1.44 |  | neutral | 43 | 2.84 | 1.40 |  | neutral | 40 | 2.60 | 1.23 |  | neutral | 45 | 2.93 | 1.47 |
| Kamamuta | 1 | smile | 42 | 3.35 | 1.31 |  | smile | 42 | 2.92 | 1.06 |  | smile | 40 | 3.08 | 1.64 |  | smile | 41 | 2.98 | 1.31 |
| Kamamuta | 2 | neutral | 42 | 2.32 | 1.19 |  | neutral | 41 | 2.54 | 1.38 |  | neutral | 41 | 2.55 | 1.50 |  | neutral | 39 | 2.79 | 1.42 |
| Kamamuta | 2 | smile | 44 | 3.01 | 1.42 |  | smile | 43 | 3.14 | 1.57 |  | smile | 40 | 2.80 | 1.33 |  | smile | 35 | 2.91 | 1.38 |
| Kamamuta | 3 | neutral | 38 | 2.52 | 1.52 |  | neutral | 40 | 2.55 | 1.43 |  | neutral | 43 | 2.74 | 1.35 |  | neutral | 39 | 2.82 | 1.29 |
| Kamamuta | 3 | smile | 37 | 3.36 | 1.54 |  | smile | 46 | 3.04 | 1.44 |  | smile | 43 | 3.12 | 1.44 |  | smile | 40 | 2.94 | 1.32 |
| Kamamuta | 4 | neutral | 39 | 2.85 | 1.59 |  | neutral | 38 | 2.54 | 1.39 |  | neutral | 43 | 2.33 | 1.31 |  | neutral | 39 | 2.90 | 1.46 |
| Kamamuta | 4 | smile | 42 | 3.00 | 1.48 |  | smile | 44 | 3.14 | 1.40 |  | smile | 44 | 2.98 | 1.66 |  | smile | 39 | 3.57 | 1.35 |
| Kamamuta | 5 | neutral | 37 | 2.75 | 1.18 |  | neutral | 38 | 2.45 | 1.31 |  | neutral | 40 | 2.53 | 1.36 |  | neutral | 37 | 3.09 | 1.35 |
| Kamamuta | 5 | smile | 39 | 3.00 | 1.45 |  | smile | 38 | 3.05 | 1.48 |  | smile | 45 | 2.94 | 1.44 |  | smile | 43 | 3.55 | 1.61 |
| Admiration | 1 | neutral | 40 | 2.58 | 1.33 |  | neutral | 43 | 2.63 | 1.31 |  | neutral | 40 | 2.40 | 1.13 |  | neutral | 45 | 2.62 | 1.48 |
| Admiration | 1 | smile | 42 | 3.49 | 1.40 |  | smile | 42 | 2.98 | 1.17 |  | smile | 40 | 3.03 | 1.65 |  | smile | 41 | 3.17 | 1.53 |
| Admiration | 2 | neutral | 42 | 2.27 | 1.31 |  | neutral | 41 | 2.37 | 1.34 |  | neutral | 41 | 2.57 | 1.51 |  | neutral | 39 | 2.56 | 1.34 |
| Admiration | 2 | smile | 44 | 2.92 | 1.51 |  | smile | 43 | 3.04 | 1.53 |  | smile | 40 | 2.65 | 1.55 |  | smile | 35 | 2.91 | 1.35 |
| Admiration | 3 | neutral | 38 | 2.40 | 1.49 |  | neutral | 40 | 2.30 | 1.22 |  | neutral | 43 | 2.41 | 1.34 |  | neutral | 39 | 2.80 | 1.32 |
| Admiration | 3 | smile | 37 | 3.24 | 1.49 |  | smile | 46 | 2.72 | 1.39 |  | smile | 43 | 3.12 | 1.35 |  | smile | 40 | 2.74 | 1.26 |
| Admiration | 4 | neutral | 39 | 2.62 | 1.60 |  | neutral | 38 | 2.46 | 1.45 |  | neutral | 43 | 2.05 | 1.28 |  | neutral | 39 | 2.97 | 1.47 |
| Admiration | 4 | smile | 42 | 2.98 | 1.53 |  | smile | 44 | 3.07 | 1.38 |  | smile | 44 | 2.94 | 1.68 |  | smile | 39 | 3.50 | 1.30 |
| Admiration | 5 | neutral | 37 | 2.55 | 1.26 |  | neutral | 38 | 2.45 | 1.29 |  | neutral | 40 | 2.60 | 1.42 |  | neutral | 37 | 2.85 | 1.41 |
| Admiration | 5 | smile | 39 | 2.73 | 1.37 |  | smile | 38 | 2.96 | 1.39 |  | smile | 45 | 2.84 | 1.41 |  | smile | 43 | 3.50 | 1.66 |
| Liking | 1 | neutral | 40 | 3.20 | 1.63 |  | neutral | 43 | 3.23 | 1.36 |  | neutral | 40 | 3.12 | 1.39 |  | neutral | 45 | 2.98 | 1.38 |
| Liking | 1 | smile | 42 | 4.33 | 1.52 |  | smile | 42 | 3.87 | 1.17 |  | smile | 40 | 3.85 | 1.65 |  | smile | 41 | 3.71 | 1.46 |
| Liking | 2 | neutral | 42 | 2.65 | 1.42 |  | neutral | 41 | 3.12 | 1.45 |  | neutral | 41 | 3.00 | 1.50 |  | neutral | 39 | 3.15 | 1.42 |
| Liking | 2 | smile | 44 | 3.21 | 1.61 |  | smile | 43 | 4.05 | 1.48 |  | smile | 40 | 3.70 | 1.43 |  | smile | 35 | 3.68 | 1.41 |
| Liking | 3 | neutral | 38 | 2.77 | 1.60 |  | neutral | 40 | 2.78 | 1.48 |  | neutral | 43 | 2.99 | 1.51 |  | neutral | 39 | 3.31 | 1.33 |
| Liking | 3 | smile | 37 | 3.82 | 1.25 |  | smile | 46 | 3.71 | 1.38 |  | smile | 43 | 4.13 | 1.40 |  | smile | 40 | 3.69 | 1.32 |
| Liking | 4 | neutral | 39 | 3.10 | 1.74 |  | neutral | 38 | 2.91 | 1.51 |  | neutral | 43 | 2.47 | 1.17 |  | neutral | 39 | 3.41 | 1.37 |
| Liking | 4 | smile | 42 | 3.75 | 1.61 |  | smile | 44 | 3.76 | 1.32 |  | smile | 44 | 3.89 | 1.84 |  | smile | 39 | 4.24 | 1.30 |
| Liking | 5 | neutral | 37 | 3.28 | 1.40 |  | neutral | 38 | 3.11 | 1.28 |  | neutral | 40 | 3.17 | 1.32 |  | neutral | 37 | 3.54 | 1.50 |
| Liking | 5 | smile | 39 | 3.57 | 1.59 |  | smile | 38 | 3.55 | 1.66 |  | smile | 45 | 3.61 | 1.64 |  | smile | 43 | 4.18 | 1.68 |
| Approach | 1 | neutral | 40 | 3.34 | 1.58 |  | neutral | 43 | 3.57 | 1.29 |  | neutral | 40 | 3.41 | 1.47 |  | neutral | 45 | 3.57 | 1.57 |
| Approach | 1 | smile | 42 | 4.14 | 1.51 |  | smile | 42 | 3.86 | 1.16 |  | smile | 40 | 3.79 | 1.53 |  | smile | 41 | 4.07 | 1.42 |
| Approach | 2 | neutral | 42 | 2.73 | 1.49 |  | neutral | 41 | 3.88 | 1.57 |  | neutral | 41 | 2.99 | 1.72 |  | neutral | 39 | 3.28 | 1.64 |
| Approach | 2 | smile | 44 | 3.40 | 1.64 |  | smile | 43 | 4.06 | 1.58 |  | smile | 40 | 3.86 | 1.56 |  | smile | 35 | 3.46 | 1.45 |
| Approach | 3 | neutral | 38 | 3.06 | 1.64 |  | neutral | 40 | 2.97 | 1.57 |  | neutral | 43 | 3.05 | 1.41 |  | neutral | 39 | 3.90 | 1.24 |
| Approach | 3 | smile | 37 | 3.80 | 1.24 |  | smile | 46 | 3.67 | 1.31 |  | smile | 43 | 4.09 | 1.40 |  | smile | 40 | 3.70 | 1.25 |
| Approach | 4 | neutral | 39 | 3.53 | 2.00 |  | neutral | 38 | 3.30 | 1.68 |  | neutral | 43 | 3.06 | 1.44 |  | neutral | 39 | 3.63 | 1.33 |
| Approach | 4 | smile | 42 | 4.06 | 1.57 |  | smile | 44 | 3.89 | 1.35 |  | smile | 44 | 3.84 | 1.66 |  | smile | 39 | 4.31 | 1.23 |
| Approach | 5 | neutral | 37 | 3.57 | 1.57 |  | neutral | 38 | 3.49 | 1.59 |  | neutral | 40 | 3.44 | 1.40 |  | neutral | 37 | 3.73 | 1.65 |
| Approach | 5 | smile | 39 | 3.74 | 1.70 |  | smile | 38 | 3.70 | 1.74 |  | smile | 45 | 3.56 | 1.50 |  | smile | 43 | 4.04 | 1.59 |
| Avoidance | 1 | neutral | 40 | 2.68 | 1.55 |  | neutral | 43 | 2.60 | 1.30 |  | neutral | 40 | 2.86 | 1.42 |  | neutral | 45 | 2.66 | 1.63 |
| Avoidance | 1 | smile | 42 | 2.21 | 1.44 |  | smile | 42 | 2.26 | 1.43 |  | smile | 40 | 2.03 | 1.09 |  | smile | 41 | 2.10 | 1.14 |
| Avoidance | 2 | neutral | 42 | 3.04 | 1.60 |  | neutral | 41 | 2.12 | 1.43 |  | neutral | 41 | 2.85 | 1.80 |  | neutral | 39 | 2.65 | 1.64 |
| Avoidance | 2 | smile | 44 | 2.67 | 1.84 |  | smile | 43 | 2.12 | 1.40 |  | smile | 40 | 2.46 | 1.35 |  | smile | 35 | 2.59 | 1.39 |
| Avoidance | 3 | neutral | 38 | 2.78 | 1.82 |  | neutral | 40 | 2.74 | 1.41 |  | neutral | 43 | 2.58 | 1.27 |  | neutral | 39 | 2.47 | 1.15 |
| Avoidance | 3 | smile | 37 | 2.44 | 1.27 |  | smile | 46 | 2.12 | 1.38 |  | smile | 43 | 2.28 | 1.30 |  | smile | 40 | 2.19 | 1.40 |
| Avoidance | 4 | neutral | 39 | 2.63 | 1.82 |  | neutral | 38 | 2.68 | 1.49 |  | neutral | 43 | 2.76 | 1.25 |  | neutral | 39 | 2.47 | 1.25 |
| Avoidance | 4 | smile | 42 | 2.14 | 1.38 |  | smile | 44 | 2.26 | 1.31 |  | smile | 44 | 2.40 | 1.35 |  | smile | 39 | 2.28 | 1.36 |
| Avoidance | 5 | neutral | 37 | 2.57 | 1.68 |  | neutral | 38 | 2.20 | 1.50 |  | neutral | 40 | 2.48 | 1.43 |  | neutral | 37 | 2.11 | 1.45 |
| Avoidance | 5 | smile | 39 | 2.31 | 1.49 |  | smile | 38 | 1.95 | 1.31 |  | smile | 45 | 2.55 | 1.31 |  | smile | 43 | 2.07 | 1.20 |
| Donating | 1 | neutral | 40 | 2.40 | 1.51 |  | neutral | 43 | 2.70 | 1.54 |  | neutral | 40 | 2.76 | 1.26 |  | neutral | 45 | 2.33 | 1.32 |
| Donating | 1 | smile | 42 | 3.07 | 1.55 |  | smile | 42 | 2.45 | 1.26 |  | smile | 40 | 2.08 | 1.12 |  | smile | 41 | 2.55 | 1.33 |
| Donating | 2 | neutral | 42 | 2.12 | 1.38 |  | neutral | 41 | 2.85 | 1.74 |  | neutral | 41 | 2.65 | 1.74 |  | neutral | 39 | 2.83 | 1.78 |
| Donating | 2 | smile | 44 | 2.43 | 1.54 |  | smile | 43 | 2.51 | 1.54 |  | smile | 40 | 2.66 | 1.43 |  | smile | 35 | 2.39 | 1.17 |
| Donating | 3 | neutral | 38 | 2.72 | 1.59 |  | neutral | 40 | 2.13 | 1.37 |  | neutral | 43 | 2.78 | 1.37 |  | neutral | 39 | 2.81 | 1.72 |
| Donating | 3 | smile | 37 | 2.51 | 1.34 |  | smile | 46 | 2.51 | 1.51 |  | smile | 43 | 3.05 | 1.62 |  | smile | 40 | 2.63 | 1.35 |
| Donating | 4 | neutral | 39 | 2.68 | 1.94 |  | neutral | 38 | 2.46 | 1.65 |  | neutral | 43 | 2.30 | 1.23 |  | neutral | 39 | 2.79 | 1.34 |
| Donating | 4 | smile | 42 | 2.52 | 1.67 |  | smile | 44 | 2.69 | 1.30 |  | smile | 44 | 2.55 | 1.74 |  | smile | 39 | 3.19 | 1.42 |
| Donating | 5 | neutral | 37 | 2.78 | 1.63 |  | neutral | 38 | 2.50 | 1.40 |  | neutral | 40 | 2.61 | 1.29 |  | neutral | 37 | 3.15 | 1.67 |
| Donating | 5 | smile | 39 | 2.36 | 1.41 |  | smile | 38 | 2.24 | 1.37 |  | smile | 45 | 2.50 | 1.38 |  | smile | 43 | 2.89 | 1.93 |
| Volunteering | 1 | neutral | 40 | 3.65 | 2.02 |  | neutral | 43 | 3.57 | 1.63 |  | neutral | 40 | 3.78 | 1.46 |  | neutral | 45 | 3.34 | 1.58 |
| Volunteering | 1 | smile | 42 | 3.96 | 1.82 |  | smile | 42 | 3.60 | 1.50 |  | smile | 40 | 3.05 | 1.37 |  | smile | 41 | 3.91 | 1.41 |
| Volunteering | 2 | neutral | 42 | 3.09 | 1.74 |  | neutral | 41 | 3.94 | 1.85 |  | neutral | 41 | 4.02 | 1.95 |  | neutral | 39 | 3.42 | 1.90 |
| Volunteering | 2 | smile | 44 | 3.48 | 1.76 |  | smile | 43 | 3.40 | 1.81 |  | smile | 40 | 3.50 | 1.70 |  | smile | 35 | 3.39 | 1.58 |
| Volunteering | 3 | neutral | 38 | 3.47 | 1.67 |  | neutral | 40 | 2.94 | 1.63 |  | neutral | 43 | 3.68 | 1.55 |  | neutral | 39 | 4.23 | 1.69 |
| Volunteering | 3 | smile | 37 | 3.34 | 1.41 |  | smile | 46 | 3.50 | 1.58 |  | smile | 43 | 4.00 | 1.53 |  | smile | 40 | 3.57 | 1.70 |
| Volunteering | 4 | neutral | 39 | 3.56 | 1.99 |  | neutral | 38 | 3.75 | 1.83 |  | neutral | 43 | 3.40 | 1.51 |  | neutral | 39 | 3.83 | 1.52 |
| Volunteering | 4 | smile | 42 | 3.69 | 1.93 |  | smile | 44 | 3.86 | 1.68 |  | smile | 44 | 3.55 | 1.68 |  | smile | 39 | 4.32 | 1.52 |
| Volunteering | 5 | neutral | 37 | 3.49 | 1.70 |  | neutral | 38 | 3.46 | 1.71 |  | neutral | 40 | 3.73 | 1.74 |  | neutral | 37 | 3.94 | 1.67 |
| Volunteering | 5 | smile | 39 | 3.42 | 1.87 |  | smile | 38 | 3.31 | 1.68 |  | smile | 45 | 3.57 | 1.65 |  | smile | 43 | 3.87 | 1.74 |

*Note.* *M* and *SD* are used to represent mean and standard deviation, respectively.

**Supplementary Table S4.1**

*Descriptive Statistics and Correlation Analyses with Main Variables (Study 4, Total Sample).*

| Variable | *M* | *SD* | 1 | 2 | 3 | 4 | 5 | 6 | 7 | 8 | 9 | 10 | 11 |
| --- | --- | --- | --- | --- | --- | --- | --- | --- | --- | --- | --- | --- | --- |
| 1. Gender | - | - |  |  |  |  |  |  |  |  |  |  |  |
|  |  |  |  |  |  |  |  |  |  |  |  |  |  |
| 2. Age | 47.17 | 13.13 | -.27** |  |  |  |  |  |  |  |  |  |  |
|  |  |  | [-.31, -.22] |  |  |  |  |  |  |  |  |  |  |
| 3. Warmth | 4.49 | 1.23 | -.04 | .14** |  |  |  |  |  |  |  |  |  |
|  |  |  | [-.09, .01] | [.09, .18] |  |  |  |  |  |  |  |  |  |
| 4. Competence | 4.49 | 1.04 | -.01 | .07** | .76** |  |  |  |  |  |  |  |  |
|  |  |  | [-.06, .04] | [.02, .12] | [.74, .78] |  |  |  |  |  |  |  |  |
| 5. Liking | 3.44 | 1.52 | -.08** | .18** | .67** | .58** |  |  |  |  |  |  |  |
|  |  |  | [-.13, -.03] | [.13, .22] | [.64, .69] | [.54, .61] |  |  |  |  |  |  |  |
| 6. Kama muta | 2.88 | 1.43 | -.02 | .11** | .47** | .43** | .74** |  |  |  |  |  |  |
|  |  |  | [-.07, .03] | [.06, .15] | [.43, .51] | [.39, .47] | [.71, .76] |  |  |  |  |  |  |
| 7. Admiration | 2.78 | 1.44 | -.05* | .08** | .50** | .47** | .78** | .87** |  |  |  |  |  |
|  |  |  | [-.10, -.00] | [.03, .13] | [.46, .53] | [.43, .50] | [.76, .79] | [.86, .88] |  |  |  |  |  |
| 8. Anger | 1.77 | 1.18 | -.01 | -.04 | -.30** | -.21** | -.04 | .15** | .14** |  |  |  |  |
|  |  |  | [-.06, .04] | [-.09, .01] | [-.34, -.26] | [-.26, -.16] | [-.09, .00] | [.10, .19] | [.10, .19] |  |  |  |  |
| 9. Approach | 3.62 | 1.54 | -.09** | .13** | .61** | .57** | .71** | .57** | .61** | -.12** |  |  |  |
|  |  |  | [-.14, -.04] | [.08, .18] | [.58, .64] | [.54, .60] | [.68, .73] | [.54, .60] | [.58, .64] | [-.16, -.07] |  |  |  |
| 10. Avoidance | 2.43 | 1.44 | .00 | -.07** | -.44** | -.34** | -.32** | -.13** | -.14** | .46** | -.40** |  |  |
|  |  |  | [-.04, .05] | [-.12, -.02] | [-.48, -.40] | [-.39, -.30] | [-.37, -.28] | [-.18, -.09] | [-.19, -.10] | [.42, .50] | [-.44, -.36] |  |  |
| 11. Donation | 2.60 | 1.51 | -.01 | .03 | .28** | .31** | .45** | .40** | .42** | .00 | .47** | -.23** |  |
|  |  |  | [-.05, .04] | [-.02, .08] | [.23, .32] | [.26, .35] | [.41, .48] | [.36, .44] | [.38, .46] | [-.05, .05] | [.43, .51] | [-.27, -.18] |  |
| 12. Volunteering | 3.61 | 1.69 | .01 | .07** | .39** | .39** | .49** | .39** | .40** | -.13** | .60** | -.38** | .66** |
|  |  |  | [-.04, .06] | [.02, .11] | [.35, .43] | [.35, .43] | [.45, .53] | [.35, .43] | [.36, .44] | [-.18, -.09] | [.57, .63] | [-.42, -.33] | [.64, .69] |

*Note.* Approach = Approach intentions; Avoidance = Avoidance intentions; Donation = Donation intentions; Volunteering = Volunteering intentions*. M* and *SD* are used to represent mean and standard deviation, respectively. Gender is coded 1 for female and 2 for male participants. Values in square brackets indicate the 95% confidence interval for each correlation. * indicates *p* < .05. ** indicates *p* < .01.

**Supplementary Table S4.2**

*Descriptive Statistics and Correlation Analyses with Main Variables (Study 4, Group membership = Ingroup).*

| Variable | *M* | *SD* | 1 | 2 | 3 | 4 | 5 | 6 | 7 | 8 | 9 | 10 | 11 |
| --- | --- | --- | --- | --- | --- | --- | --- | --- | --- | --- | --- | --- | --- |
| 1. Gender | - | - |  |  |  |  |  |  |  |  |  |  |  |
|  |  |  |  |  |  |  |  |  |  |  |  |  |  |
| 2. Age | 47.74 | 13.38 | -.25** |  |  |  |  |  |  |  |  |  |  |
|  |  |  | [-.34, -.16] |  |  |  |  |  |  |  |  |  |  |
| 3. Warmth | 4.45 | 1.24 | -.13** | .22** |  |  |  |  |  |  |  |  |  |
|  |  |  | [-.22, -.03] | [.13, .31] |  |  |  |  |  |  |  |  |  |
| 4. Competence | 4.51 | 1.03 | -.13* | .12* | .74** |  |  |  |  |  |  |  |  |
|  |  |  | [-.22, -.03] | [.03, .22] | [.69, .78] |  |  |  |  |  |  |  |  |
| 5. Liking | 3.39 | 1.56 | -.19** | .24** | .68** | .59** |  |  |  |  |  |  |  |
|  |  |  | [-.28, -.10] | [.14, .32] | [.62, .73] | [.53, .65] |  |  |  |  |  |  |  |
| 6. Kama muta | 2.77 | 1.44 | -.12* | .15** | .46** | .44** | .73** |  |  |  |  |  |  |
|  |  |  | [-.22, -.03] | [.06, .24] | [.38, .53] | [.36, .52] | [.68, .77] |  |  |  |  |  |  |
| 7. Admiration | 2.66 | 1.46 | -.16** | .13** | .49** | .50** | .77** | .88** |  |  |  |  |  |
|  |  |  | [-.25, -.07] | [.03, .22] | [.42, .56] | [.42, .56] | [.73, .81] | [.86, .90] |  |  |  |  |  |
| 8. Anger | 1.71 | 1.10 | .01 | -.11* | -.24** | -.10* | -.02 | .18** | .19** |  |  |  |  |
|  |  |  | [-.09, .11] | [-.20, -.01] | [-.33, -.15] | [-.19, -.00] | [-.12, .07] | [.09, .27] | [.09, .28] |  |  |  |  |
| 9. Approach | 3.51 | 1.54 | -.14** | .20** | .60** | .58** | .70** | .57** | .62** | -.05 |  |  |  |
|  |  |  | [-.23, -.04] | [.11, .29] | [.53, .65] | [.51, .64] | [.65, .75] | [.50, .63] | [.56, .68] | [-.15, .04] |  |  |  |
| 10. Avoidance | 2.53 | 1.37 | .05 | -.17** | -.34** | -.18** | -.23** | -.06 | -.06 | .40** | -.27** |  |  |
|  |  |  | [-.05, .14] | [-.26, -.07] | [-.42, -.25] | [-.27, -.09] | [-.32, -.14] | [-.15, .04] | [-.15, .04] | [.32, .48] | [-.36, -.18] |  |  |
| 11. Donation | 2.59 | 1.44 | .04 | .01 | .13** | .14** | .29** | .28** | .29** | .17** | .30** | -.05 |  |
|  |  |  | [-.06, .13] | [-.09, .10] | [.03, .22] | [.05, .24] | [.20, .37] | [.19, .36] | [.20, .37] | [.07, .26] | [.21, .39] | [-.14, .05] |  |
| 12. Volunteering | 3.63 | 1.63 | .04 | .10* | .22** | .19** | .33** | .27** | .25** | -.01 | .42** | -.21** | .61** |
|  |  |  | [-.05, .14] | [.00, .19] | [.13, .31] | [.10, .28] | [.24, .42] | [.18, .36] | [.16, .34] | [-.11, .08] | [.33, .49] | [-.30, -.12] | [.55, .67] |

*Note.* Approach = Approach intentions; Avoidance = Avoidance intentions; Donation = Donation intentions; Volunteering = Volunteering intentions*. M* and *SD* are used to represent mean and standard deviation, respectively. Gender is coded 1 for female and 2 for male participants. Values in square brackets indicate the 95% confidence interval for each correlation. * indicates *p* < .05. ** indicates *p* < .01.

**Supplementary Table S4.3**

*Descriptive Statistics and Correlation Analyses with Main Variables (Study 4, Group membership = Outgroup).*

| Variable | *M* | *SD* | 1 | 2 | 3 | 4 | 5 | 6 | 7 | 8 | 9 | 10 | 11 |
| --- | --- | --- | --- | --- | --- | --- | --- | --- | --- | --- | --- | --- | --- |
| 1. Gender | - | - |  |  |  |  |  |  |  |  |  |  |  |
|  |  |  |  |  |  |  |  |  |  |  |  |  |  |
| 2. Age | 46.97 | 13.04 | -.27** |  |  |  |  |  |  |  |  |  |  |
|  |  |  | [-.33, -.22] |  |  |  |  |  |  |  |  |  |  |
| 3. Warmth | 4.50 | 1.22 | -.01 | .11** |  |  |  |  |  |  |  |  |  |
|  |  |  | [-.06, .05] | [.05, .16] |  |  |  |  |  |  |  |  |  |
| 4. Competence | 4.49 | 1.04 | .03 | .05 | .77** |  |  |  |  |  |  |  |  |
|  |  |  | [-.02, .09] | [-.01, .11] | [.75, .79] |  |  |  |  |  |  |  |  |
| 5. Liking | 3.46 | 1.51 | -.04 | .15** | .66** | .57** |  |  |  |  |  |  |  |
|  |  |  | [-.09, .02] | [.10, .21] | [.63, .69] | [.53, .61] |  |  |  |  |  |  |  |
| 6. Kama muta | 2.91 | 1.42 | .02 | .09** | .47** | .42** | .74** |  |  |  |  |  |  |
|  |  |  | [-.04, .08] | [.04, .15] | [.43, .52] | [.38, .47] | [.71, .76] |  |  |  |  |  |  |
| 7. Admiration | 2.82 | 1.43 | -.01 | .06* | .50** | .46** | .78** | .87** |  |  |  |  |  |
|  |  |  | [-.07, .04] | [.01, .12] | [.45, .54] | [.41, .50] | [.75, .80] | [.85, .88] |  |  |  |  |  |
| 8. Anger | 1.79 | 1.20 | -.02 | -.02 | -.32** | -.25** | -.05 | .13** | .13** |  |  |  |  |
|  |  |  | [-.07, .04] | [-.07, .04] | [-.37, -.27] | [-.30, -.19] | [-.11, .00] | [.08, .19] | [.07, .18] |  |  |  |  |
| 9. Approach | 3.65 | 1.54 | -.07* | .11** | .62** | .57** | .71** | .57** | .60** | -.14** |  |  |  |
|  |  |  | [-.13, -.01] | [.05, .16] | [.58, .65] | [.53, .61] | [.68, .74] | [.53, .61] | [.57, .64] | [-.19, -.08] |  |  |  |
| 10. Avoidance | 2.40 | 1.47 | -.01 | -.04 | -.48** | -.40** | -.35** | -.16** | -.17** | .48** | -.44** |  |  |
|  |  |  | [-.07, .04] | [-.10, .02] | [-.52, -.43] | [-.44, -.35] | [-.40, -.30] | [-.21, -.10] | [-.23, -.12] | [.43, .52] | [-.48, -.39] |  |  |
| 11. Donation | 2.60 | 1.53 | -.02 | .04 | .33** | .36** | .50** | .44** | .46** | -.05 | .53** | -.28** |  |
|  |  |  | [-.08, .04] | [-.02, .09] | [.27, .38] | [.31, .41] | [.46, .54] | [.40, .49] | [.42, .51] | [-.10, .01] | [.48, .57] | [-.33, -.23] |  |
| 12. Volunteering | 3.61 | 1.72 | -.01 | .05 | .44** | .46** | .55** | .43** | .46** | -.17** | .66** | -.43** | .68** |
|  |  |  | [-.06, .05] | [-.00, .11] | [.40, .49] | [.41, .50] | [.51, .58] | [.38, .48] | [.41, .50] | [-.23, -.12] | [.63, .69] | [-.47, -.38] | [.65, .71] |

*Note.* Approach = Approach intentions; Avoidance = Avoidance intentions; Donation = Donation intentions; Volunteering = Volunteering intentions*. M* and *SD* are used to represent mean and standard deviation, respectively. Gender is coded 1 for female and 2 for male participants. Values in square brackets indicate the 95% confidence interval for each correlation. * indicates *p* < .05. ** indicates *p* < .01.

**Supplementary Table S4.4**

*Means, Standard Deviations, and ANOVAs Results: Impact of Smiling Depictions of Immigrants of three Different Countries (Outgroups) and Spain (Ingroup) on Host Culture Members’ Cognitive Inferences, Affective Reactions, and Behavioral Intentions (Study 4, N = 1629).*

|  |  | Stimulus | |  | Comparison | | | |
| --- | --- | --- | --- | --- | --- | --- | --- | --- |
| Variable | Group | Non-smiling display  *M*(*SD*) | Smiling display  *M*(*SD*) |  | Test^1^ | *F*_(3, 1621)_ | *p* | η_p_^2^ [90% CI] |
| *Manipulation check* | | | | | | | | |
| Group Membership | Morocco | 2.54(1.54)^a^ | 2.55(1.53)^a^ |  | GE | 473.70 | < .001 | .47[.44, .49] |
|  | Ecuador | 2.33(1.41)^a^ | 2.42(1.44)^a^ |  | CE | 3.15 | .076 | .00[.00, .01] |
|  | Spain | 5.60(1.20)^c^ | 5.71(1.20)^c^ |  | IE | 0.74 | .528 | .00[.00, .00] |
|  | Romania | 4.21(1.70)^b^ | 4.52(1.61)^b^ |  |  |  |  |  |
| Neutral | Morocco | 4.89(1.41) | 2.84(1.24)^a^ |  | GE | 9.18 | < .001 | .02[.01, .03] |
|  | Ecuador | 5.05(1.37) | 3.53(1.49)^c^ |  | CE | 661.27 | < .001 | .29[.26, .32] |
|  | Spain | 4.97(1.40) | 3.35(1.40)^b,c^ |  | IE | 2.89 | .034 | .01[.00, .01] |
|  | Romania | 4.80(1.32) | 3.01(1.29)^a,b^ |  |  |  |  |  |
| Happy | Morocco | 2.98(1.27) | 5.14(1.12)^b^ |  | GE | 10.82 | < .001 | .02[.01, .03] |
|  | Ecuador | 2.80(1.28) | 4.63(1.17)^a^ |  | CE | 1205.49 | < .001 | .43[.40, .45] |
|  | Spain | 3.04(1.35) | 5.09(0.99)^b^ |  | IE | 1.57 | .195 | .00[.00, .01] |
|  | Romania | 3.08(1.28) | 5.21(0.98)^b^ |  |  |  |  |  |
| *Cognitive Inferences* | | | | | | | | |
| Warmth | Morocco | 3.90(1.33) | 4.91(1.10) |  | GE | 1.44 | .230 | .00[.00, .01] |
|  | Ecuador | 4.12(1.17) | 4.98(1.04) |  | CE | 329.63 | < .001 | .17[.14, .20] |
|  | Spain | 3.86(1.17) | 5.03(1.01) |  | IE | 1.43 | .232 | .00[.00, .01] |
|  | Romania | 4.04(1.16) | 5.01(0.92) |  |  |  |  |  |
| Competence | Morocco | 4.26(1.16) | 4.57(1.02) |  | GE | 1.31 | .270 | .00[.00, .01] |
|  | Ecuador | 4.31(1.06) | 4.66(1.04) |  | CE | 45.79 | < .001 | .03[.02, .04] |
|  | Spain | 4.25(0.97) | 4.75(1.03) |  | IE | 1.47 | .222 | .00[.00, .01] |
|  | Romania | 4.45(0.99) | 4.66(0.89) |  |  |  |  |  |
| *Affective Reactions* | | | | | | | | |
| Liking | Morocco | 2.99(1.57) | 3.73(1.56) |  | GE | 1.87 | .132 | .00[.00, .01] |
|  | Ecuador | 3.03(1.41) | 3.79(1.40) |  | CE | 107.77 | < .001 | .06[.04, .08] |
|  | Spain | 2.94(1.39) | 3.84(1.60) |  | IE | 0.52 | .668 | .00[.00, .00] |
|  | Romania | 3.27(1.40) | 3.91(1.45) |  |  |  |  |  |
| Anger | Morocco | 2.02(1.34) | 1.78(1.28)^b^ |  | GE | 3.36 | .018 | .01[.00, .01] |
|  | Ecuador | 1.77(1.17) | 1.55(1.01)^a,b^ |  | CE | 37.17 | < .001 | .02[.01, .04] |
|  | Spain | 1.97(1.24) | 1.46(0.89)a |  | IE | 1.64 | .179 | .00[.00, .01] |
|  | Romania | 2.04(1.31) | 1.60(0.99)^a,b^ |  |  |  |  |  |
| Kama muta | Morocco | 2.58(1.39) | 3.14(1.44) |  | GE | 3.08 | .026 | .01[.00, .01] |
|  | Ecuador | 2.59(1.38) | 3.06(1.39) |  | CE | 39.75 | < .001 | .02[.01, .04] |
|  | Spain | 2.55(1.35) | 2.99(1.50) |  | IE | 0.58 | .629 | .00[.00, .00] |
|  | Romania | 2.90(1.39) | 3.20(1.42) |  |  |  |  |  |
| Admiration | Morocco | 2.48(1.40)^a,b^ | 3.07(1.47) |  | GE | 3.77 | .010 | .01[.00, .01] |
|  | Ecuador | 2.44(1.31)^a,b^ | 2.95(1.37) |  | CE | 52.70 | < .001 | .03[.02, .05] |
|  | Spain | 2.40(1.34)^a^ | 2.92(1.53) |  | IE | 0.25 | .861 | .00[.00, .00] |
|  | Romania | 2.76(1.40)^b^ | 3.17(1.45) |  |  |  |  |  |
| *Behavioral Intentions* | | | | | | | | |
| Approach | Morocco | 3.23(1.68)^a,b^ | 3.82(1.56) |  | GE | 2.63 | .049 | .00[.00, .01] |
|  | Ecuador | 3.45(1.56)^a,b^ | 3.84(1.43) |  | CE | 41.24 | < .001 | .02[.01, .04] |
|  | Spain | 3.19(1.49)^a^ | 3.82(1.53) |  | IE | 1.09 | .354 | .00[.00, .01] |
|  | Romania | 3.62(1.50)^b^ | 3.93(1.41) |  |  |  |  |  |
| Avoidance | Morocco | 2.75(1.69) | 2.36(1.50) |  | GE | 2.93 | .032 | .01[.00, .01] |
|  | Ecuador | 2.47(1.43) | 2.15(1.36) |  | CE | 21.56 | < .001 | .01[.01, .02] |
|  | Spain | 2.71(1.44) | 2.35(1.29) |  | IE | 0.18 | .909 | .00[.00, .00] |
|  | Romania | 2.48(1.44) | 2.23(1.30) |  |  |  |  |  |
| Donation | Morocco | 2.53(1.62) | 2.58(1.52) |  | GE | 1.95 | .119 | .00[.00, .01] |
|  | Ecuador | 2.53(1.55) | 2.49(1.40) |  | CE | 0.06 | .804 | .00[.00, .00] |
|  | Spain | 2.62(1.39) | 2.57(1.50) |  | IE | 0.10 | .961 | .00[.00, 1.00] |
|  | Romania | 2.77(1.58) | 2.74(1.49) |  |  |  |  |  |
| Volunteering | Morocco | 3.45(1.82) | 3.58(1.77) |  | GE | 2.01 | .111 | .00[.00, .01] |
|  | Ecuador | 3.53(1.75) | 3.54(1.65) |  | CE | 0.01 | .907 | .00[.00, .00] |
|  | Spain | 3.72(1.65) | 3.54(1.61) |  | IE | 0.68 | .567 | .00[.00, .00] |
|  | Romania | 3.74(1.69) | 3.82(1.61) |  |  |  |  |  |

*Note.* Approach = Approach intentions; Avoidance = Avoidance intentions; Donation = Donation intentions; Volunteering = Volunteering intentions; Ingroup = the Spanish man; Outgroup =  the Romanian man. *M*(*SD*) represent means and standard deviations, respectively. η_p_^2^ [90% CI] represent eta squared and the lower- and upper-limit of its confidence interval. ^1^, shows the ANOVA contrasts: GE = Group membership effects; CE = Condition effects (smiling vs. non-smiling display); and IE = Interaction effects (Group membership * Condition). The subscripts indicate post-hoc (Tukey) tests compared within the levels of the stimulus variable; different letters indicate a difference of at least *p* < .05. Number of participants by condition: Neutral-Morocco: *n =* 196; Neutral-Ecuador: *n =* 200; Neutral-Spain: *n =* 207; Neutral-Romania: *n =* 199;
Smiling-Morocco: *n =* 204; Smiling-Ecuador: *n =* 213; Smiling-Spain: *n =* 212; Smiling-Romania: *n =* 198.

**Supplementary Figure S4.1**

*Impact of Smiling Depictions of Immigrants (four Countries) on Host Culture Members’ Cognitive Inferences, Affective Reactions, and Behavioral Intentions (Study 4).*


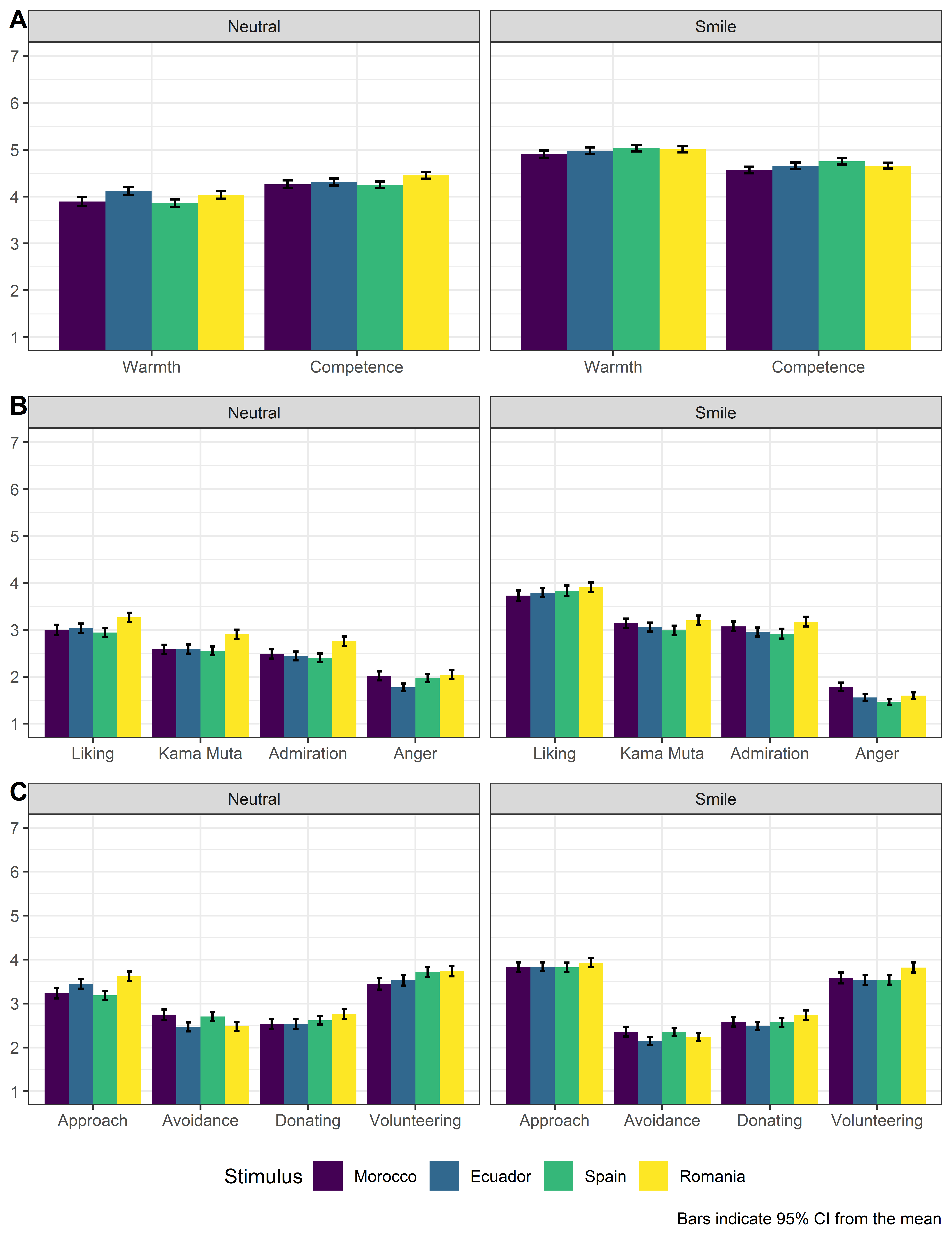


**Supplementary Table S4.5**

*MG−Path Analyses of the Effects of Smiling on Approach and Avoidance Intentions via Inferences of Warmth and Competence (Study 4).*

| Effect | Group = Ingroup | | | | |  |  | Group = Outgroup | | | | |  |
| --- | --- | --- | --- | --- | --- | --- | --- | --- | --- | --- | --- | --- | --- |
|  | *B* | *SE* | *p* | 95% CI | | β |  | *B* | *SE* | *p* | 95% CI | | β |
|  |  |  |  | *LL* | *UL* |  |  |  |  |  | *LL* | *UL* |  |
| *Approach intentions* | | | | | | | | | | | | | |
| Total effect | 0.428 | 0.088 | < .001 | 0.256 | 0.600 | .139 |  | 0.638 | 0.147 | < .001 | 0.350 | 0.927 | .207 |
| Direct effects |  |  |  |  |  |  |  |  |  |  |  |  |  |
| Condition on approach | -0.175 | 0.135 | .195 | -0.438 | 0.089 | -.057 |  | -0.242 | 0.077 | .002 | -0.392 | -0.091 | -.079 |
| Warmth on approach | 0.504 | 0.078 | < .001 | 0.350 | 0.657 | .406 |  | 0.616 | 0.049 | < .001 | 0.520 | 0.712 | .489 |
| Competence on approach | 0.439 | 0.086 | < .001 | 0.270 | 0.607 | .292 |  | 0.303 | 0.054 | < .001 | 0.198 | 0.408 | .205 |
| Condition on warmth | 1.176 | 0.107 | < .001 | 0.967 | 1.386 | .474 |  | 0.946 | 0.065 | < .001 | 0.820 | 1.073 | .388 |
| Condition on competence | 0.502 | 0.097 | < .001 | 0.311 | 0.693 | .244 |  | 0.286 | 0.059 | < .001 | 0.170 | 0.402 | .138 |
| Indirect Effects via |  |  |  |  |  |  |  |  |  |  |  |  |  |
| Warmth | 0.593 | 0.107 | < .001 | 0.384 | 0.802 | .192 |  | 0.583 | 0.061 | < .001 | 0.463 | 0.703 | 0.190 |
| Competence | 0.220 | 0.061 | < .001 | 0.101 | 0.339 | .071 |  | 0.087 | 0.024 | < .001 | 0.040 | 0.133 | 0.028 |
| *Avoidance intentions* | | | | | | | | | | | | | |
| Total effect | -0.321 | 0.084 | < .001 | -0.485 | -0.157 | -.109 |  | -0.356 | 0.133 | .007 | -0.617 | -0.096 | -.130 |
| Direct effects |  |  |  |  |  |  |  |  |  |  |  |  |  |
| Condition on avoidance | 0.168 | 0.145 | .246 | -0.116 | 0.452 | .061 |  | 0.239 | 0.083 | .004 | 0.076 | 0.402 | .082 |
| Warmth on avoidance | -0.540 | 0.084 | < .001 | -0.705 | -0.375 | -.489 |  | -0.575 | 0.053 | < .001 | -0.679 | -0.471 | -.478 |
| Competence on avoidance | 0.222 | 0.092 | .016 | 0.041 | 0.403 | .166 |  | -0.056 | 0.058 | .338 | -0.170 | 0.058 | -.039 |
| Indirect effects via |  |  |  |  |  |  |  |  |  |  |  |  |  |
| Warmth | -0.636 | 0.115 | < .001 | -0.860 | -0.411 | -.232 |  | -0.544 | 0.063 | < .001 | -0.667 | -0.421 | -.186 |
| Competence | 0.111 | 0.051 | .029 | 0.011 | 0.211 | .041 |  | -0.016 | 0.017 | .347 | -0.049 | 0.017 | -.005 |
| *Model fit* | | | | | | | | | | | | | |
| *CFI* | 1.000 | | | | | | | | | | | | |
| *TLI* | 1.000 | | | | | | | | | | | | |
| *RMSEA* (90% CI) | 0.000 (0.000, 0.000) | | | | | | | | | | | | |
| *SRMR* | 0.000 | | | | | | | | | | | | |

*Note.* Condition = Neutral vs. Smiling Display; Approach = Approach intentions; Avoidance = Avoidance intentions; *B* = Non−standardized estimates; *SE* = Standard error; β = Standardized coefficients of the model; *CFI* = Comparative Fit Index; *TLI* = Tucker−Lewis Index; *RMSEA* = Root Mean Square Error of Approximation; *SRMR* = Standardized Root Mean Square Residual.

In both cases, when participants saw the face of an ingroup member or any of the outgroups, smiling had indirect effects on approach intentions via higher perceptions of warmth and competence. Considering avoidance intentions, smiling had indirect effects on avoidance intentions (decreasing them) via greater perceptions of warmth (both ingroup and outgroup) and competence (only for the ingroup).

**Supplementary Table S4.6**

*MG−Path Analyses of the Effects of Smiling on Donation and Volunteering Intentions via Inferences of Warmth and Competence (Study 4).*

| Effect | Group = Ingroup | | | | |  |  | Group = Outgroup | | | | |  |
| --- | --- | --- | --- | --- | --- | --- | --- | --- | --- | --- | --- | --- | --- |
|  | *B* | *SE* | *p* | 95% CI | | β |  | *B* | *SE* | *p* | 95% CI | | β |
|  |  |  |  | *LL* | *UL* |  |  |  |  |  | *LL* | *UL* |  |
| *Donation intentions* | | | | | | | | | | | | | |
| Total effect | -0.011 | 0.088 | .898 | -0.183 | 0.161 | -.004 |  | -0.048 | 0.141 | .735 | -0.323 | 0.228 | -.017 |
| Direct effects |  |  |  |  |  |  |  |  |  |  |  |  |  |
| Condition on donation | -0.249 | 0.160 | .121 | -0.563 | 0.065 | -.086 |  | -0.336 | 0.092 | < .001 | -0.515 | -0.156 | -.110 |
| Warmth on donation | 0.116 | 0.093 | .212 | -0.066 | 0.299 | .100 |  | 0.242 | 0.059 | < .001 | 0.127 | 0.357 | .193 |
| Competence on donation | 0.128 | 0.102 | .211 | -0.073 | 0.328 | .091 |  | 0.333 | 0.064 | < .001 | 0.208 | 0.458 | .227 |
| Condition on warmth | 1.176 | 0.107 | < .001 | 0.967 | 1.386 | .474 |  | 0.946 | 0.065 | < .001 | 0.820 | 1.073 | .388 |
| Condition on competence | 0.502 | 0.097 | < .001 | 0.311 | 0.693 | .244 |  | 0.286 | 0.059 | < .001 | 0.170 | 0.402 | .138 |
| Indirect effects via |  |  |  |  |  |  |  |  |  |  |  |  |  |
| Warmth | 0.137 | 0.110 | .215 | -0.079 | 0.353 | .048 |  | 0.229 | 0.058 | < .001 | 0.116 | 0.342 | .075 |
| Competence | 0.064 | 0.053 | .224 | -0.039 | 0.168 | .022 |  | 0.095 | 0.027 | < .001 | 0.043 | 0.148 | .031 |
| *Volunteering intentions* | | | | | | | | | | | | | |
| Total effect | 0.071 | 0.099 | .469 | -0.122 | 0.265 | .021 |  | -0.177 | 0.158 | .263 | -0.488 | 0.133 | -.055 |
| Direct effects |  |  |  |  |  |  |  |  |  |  |  |  |  |
| Condition on volunteering | -0.663 | 0.176 | < .001 | -1.007 | -0.319 | -.204 |  | -0.456 | 0.097 | < .001 | -0.646 | -0.267 | -.133 |
| Warmth on volunteering | 0.404 | 0.102 | < .001 | 0.204 | 0.605 | .309 |  | 0.439 | 0.062 | < .001 | 0.318 | 0.560 | .312 |
| Competence on volunteering | 0.020 | 0.112 | .859 | -0.200 | 0.239 | .013 |  | 0.393 | 0.068 | < .001 | 0.260 | 0.525 | .238 |
| Indirect effects via |  |  |  |  |  |  |  |  |  |  |  |  |  |
| Warmth | 0.476 | 0.128 | < .001 | 0.226 | 0.726 | .146 |  | 0.415 | 0.065 | < .001 | 0.288 | 0.543 | .121 |
| Competence | 0.010 | 0.056 | .859 | -0.100 | 0.120 | .003 |  | 0.112 | 0.030 | < .001 | 0.053 | 0.172 | .033 |
| *Model fit* | | | | | | | | | | | | | |
| *CFI* | 1.000 | | | | | | | | | | | | |
| *TLI* | 1.000 | | | | | | | | | | | | |
| *RMSEA* (90% *CI*) | 0.000 (0.000, 0.000) | | | | | | | | | | | | |
| *SRMR* | 0.000 | | | | | | | | | | | | |

*Note.* Condition = Neutral vs. Smiling Display; Donation = Donation intentions; Volunteering = Volunteering intentions; *B* = Non−standardized estimates; *SE* = Standard error; β = Standardized coefficients of the model; *CFI* = Comparative Fit Index; *TLI* = Tucker−Lewis Index; *RMSEA* = Root Mean Square Error of Approximation; *SRMR* = Standardized Root Mean Square Residual.

We found indirect effects of smiling on donation intentions via warmth and competence, but only when the target presented as a member of the outgroup. The same pattern was observed when predicting volunteering intentions; we found indirect effects of smiling on volunteering intentions via increased warmth and competence for the outgroup member and only via increased warmth for ingroup members.

**Supplementary Table S4.7**

*MG−Path Analyses of the Effects of Smiling on Approach and Avoidance Intentions via Host Culture Member’ Affective Reactions (Study 4).*

| Effect | Group = Ingroup | | | | |  |  | Group = Outgroup | | | | |  |
| --- | --- | --- | --- | --- | --- | --- | --- | --- | --- | --- | --- | --- | --- |
|  | *B* | *SE* | *p* | 95% CI | | β |  | *B* | *SE* | *p* | 95% CI | | β |
|  |  |  |  | *LL* | *UL* |  |  |  |  |  | *LL* | *UL* |  |
| *Approach intentions* | | | | | | | | | | | | | |
| Total effect | 0.638 | 0.147 | < .001 | 0.350 | 0.927 | .207 |  | 0.428 | 0.088 | < .001 | 0.256 | 0.600 | .139 |
| Direct effects |  |  |  |  |  |  |  |  |  |  |  |  |  |
| Condition on approach | -0.008 | 0.113 | .947 | -0.229 | 0.213 | -.002 |  | -0.135 | 0.063 | .032 | -0.258 | -0.012 | -.044 |
| Liking on approach | 0.509 | 0.057 | < .001 | 0.398 | 0.621 | .516 |  | 0.562 | 0.034 | < .001 | 0.495 | 0.629 | .551 |
| Kama muta on approach | -0.017 | 0.079 | .824 | -0.172 | 0.137 | -.016 |  | 0.065 | 0.044 | .138 | -0.021 | 0.152 | .060 |
| Admiration on approach | 0.266 | 0.085 | .002 | 0.100 | 0.433 | .252 |  | 0.162 | 0.047 | .001 | 0.070 | 0.254 | .150 |
| Anger on approach | -0.120 | 0.052 | .021 | -0.221 | -0.018 | -.086 |  | -0.182 | 0.027 | < .001 | -0.234 | -0.131 | -.143 |
| Condition on liking | 0.894 | 0.146 | < .001 | 0.608 | 1.181 | .286 |  | 0.710 | 0.084 | < .001 | 0.544 | 0.875 | .235 |
| Condition on kama muta | 0.435 | 0.139 | .002 | 0.163 | 0.707 | .151 |  | 0.439 | 0.081 | < .001 | 0.281 | 0.597 | .155 |
| Condition on admiration | 0.516 | 0.140 | < .001 | 0.241 | 0.791 | .177 |  | 0.502 | 0.081 | < .001 | 0.344 | 0.660 | .176 |
| Condition on anger | -0.506 | 0.105 | < .001 | -0.711 | -0.300 | -.229 |  | -0.299 | 0.069 | < .001 | -0.433 | -0.165 | -.124 |
| Indirect effects via |  |  |  |  |  |  |  |  |  |  |  |  |  |
| Liking | 0.456 | 0.090 | < .001 | 0.279 | 0.632 | .148 |  | 0.399 | 0.053 | < .001 | 0.294 | 0.503 | .130 |
| Kama muta | -0.008 | 0.034 | .825 | -0.075 | 0.060 | -.002 |  | 0.029 | 0.020 | .152 | -0.011 | 0.068 | .009 |
| Admiration | 0.137 | 0.058 | .017 | 0.025 | 0.250 | .045 |  | 0.081 | 0.027 | .003 | 0.028 | 0.134 | .026 |
| Anger | 0.061 | 0.029 | .037 | 0.004 | 0.118 | .020 |  | 0.055 | 0.015 | < .001 | 0.026 | 0.084 | .018 |
| *Avoidance intentions* | | | | | | | | | | | | | |
| Total effect | -0.356 | 0.133 | .007 | -0.617 | -0.096 | -.130 |  | -0.321 | 0.084 | < .001 | -0.485 | -0.157 | -.109 |
| Direct effects |  |  |  |  |  |  |  |  |  |  |  |  |  |
| Condition on avoidance | 0.079 | 0.127 | .537 | -0.171 | 0.328 | .029 |  | 0.078 | 0.071 | .273 | -0.061 | 0.217 | .027 |
| Liking on avoidance | -0.278 | 0.064 | < .001 | -0.404 | -0.152 | -.316 |  | -0.382 | 0.039 | < .001 | -0.458 | -0.307 | -.393 |
| Kama muta on avoidance | -0.007 | 0.089 | .935 | -0.181 | 0.167 | -.008 |  | 0.042 | 0.050 | .396 | -0.055 | 0.140 | .041 |
| Admiration on avoidance | 0.112 | 0.096 | .240 | -0.075 | 0.300 | .119 |  | 0.036 | 0.053 | .498 | -0.068 | 0.140 | .035 |
| Anger on avoidance | 0.476 | 0.059 | < .001 | 0.362 | 0.591 | .383 |  | 0.549 | 0.030 | < .001 | 0.490 | 0.608 | .450 |
| Indirect effects via |  |  |  |  |  |  |  |  |  |  |  |  |  |
| Liking | -0.249 | 0.070 | < .001 | -0.387 | -0.111 | -.091 |  | -0.271 | 0.042 | < .001 | -0.354 | -0.188 | -.092 |
| Kama muta | -0.003 | 0.039 | .935 | -0.079 | 0.073 | -.001 |  | 0.019 | 0.022 | .402 | -0.025 | 0.062 | .006 |
| Admiration | 0.058 | 0.052 | .263 | -0.044 | 0.160 | .021 |  | 0.018 | 0.027 | .501 | -0.034 | 0.071 | .006 |
| Anger | -0.241 | 0.058 | < .001 | -0.355 | -0.127 | -.088 |  | -0.164 | 0.039 | < .001 | -0.240 | -0.088 | -.056 |
| *Model fit* | | | | | | | | | | | | | |
| *CFI* | 1.000 | | | | | | | | | | | | |
| *TLI* | 1.000 | | | | | | | | | | | | |
| *RMSEA* (90% *CI*) | 0.000 (0.000, 0.000) | | | | | | | | | | | | |
| *SRMR* | 0.000 | | | | | | | | | | | | |

*Note.* Condition = Neutral vs. Smiling Display; Approach = Approach intentions; Avoidance = Avoidance intentions; *B* = Non−standardized estimates; *SE* = Standard error; β = Standardized coefficients of the model; *CFI* = Comparative Fit Index; *TLI* = Tucker−Lewis Index; *RMSEA* = Root Mean Square Error of Approximation; *SRMR* = Standardized Root Mean Square Residual.

There were significant indirect effects of smiling on approach intentions via liking, admiration and anger both in response to an ingroup and outgroup member. Concerning avoidance intentions, there were also indirect effects through liking and anger.

**Supplementary Table S4.8**

*MG−Path Analyses of the Effects of Smiling on Donation and Volunteering Intentions via Host Culture Members’ Affective Reactions (Study 4).*

| Effect | Group = Ingroup | | | | |  |  | Group = Outgroup | | | | |  |
| --- | --- | --- | --- | --- | --- | --- | --- | --- | --- | --- | --- | --- | --- |
|  | *B* | *SE* | *p* | 95% CI | | β |  | *B* | *SE* | *p* | 95% CI | | β |
|  |  |  |  | *LL* | *UL* |  |  |  |  |  | *LL* | *UL* |  |
| *Donation intentions* | | | | | | | | | | | | | |
| Total effect | -0.177 | 0.158 | .263 | -0.488 | 0.133 | -.055 |  | 0.071 | 0.099 | .469 | -0.122 | 0.265 | .021 |
| Direct effects |  |  |  |  |  |  |  |  |  |  |  |  |  |
| Condition on donation | -0.196 | 0.142 | .167 | -0.474 | 0.082 | -.068 |  | -0.412 | 0.077 | < .001 | -0.562 | -0.261 | -.135 |
| Liking on donation | 0.221 | 0.072 | .002 | 0.081 | 0.362 | .240 |  | 0.350 | 0.042 | < .001 | 0.268 | 0.431 | .346 |
| Kama muta on donation | 0.041 | 0.099 | .676 | -0.153 | 0.235 | .041 |  | 0.090 | 0.054 | .094 | -0.015 | 0.195 | .084 |
| Admiration on donation | 0.050 | 0.107 | .638 | -0.159 | 0.259 | .051 |  | 0.166 | 0.057 | .004 | 0.053 | 0.278 | .155 |
| Anger on donation | 0.185 | 0.065 | .005 | 0.057 | 0.313 | .141 |  | -0.099 | 0.032 | .002 | -0.162 | -0.035 | -.078 |
| Condition on liking | 0.894 | 0.146 | < .001 | 0.608 | 1.181 | .286 |  | 0.710 | 0.084 | < .001 | 0.544 | 0.875 | .235 |
| Condition on kama muta | 0.435 | 0.139 | .002 | 0.163 | 0.707 | .151 |  | 0.439 | 0.081 | < .001 | 0.281 | 0.597 | .155 |
| Condition on admiration | 0.516 | 0.140 | < .001 | 0.241 | 0.791 | .177 |  | 0.502 | 0.081 | < .001 | 0.344 | 0.660 | .176 |
| Condition on anger | -0.506 | 0.105 | < .001 | -0.711 | -0.300 | -.229 |  | -0.299 | 0.069 | < .001 | -0.433 | -0.165 | -.124 |
| Indirect effects via |  |  |  |  |  |  |  |  |  |  |  |  |  |
| Liking | 0.198 | 0.072 | .006 | 0.057 | 0.339 | .069 |  | 0.248 | 0.042 | < .001 | 0.166 | 0.330 | .081 |
| Kama muta | 0.018 | 0.043 | .679 | -0.067 | 0.103 | .006 |  | 0.039 | 0.025 | .110 | -0.009 | 0.088 | .013 |
| Admiration | 0.026 | 0.055 | .641 | -0.083 | 0.135 | .009 |  | 0.083 | 0.032 | .009 | 0.021 | 0.146 | .027 |
| Anger | -0.094 | 0.038 | .014 | -0.168 | -0.019 | -.032 |  | 0.026 | 0.055 | .641 | -0.083 | 0.135 | .009 |
| *Volunteering intentions* | | | | | | | | | | | | | |
| Total effect |  |  |  |  |  |  |  |  |  |  |  |  |  |
| Direct effects |  |  |  |  |  |  |  |  |  |  |  |  |  |
| Condition on volunteering | -0.559 | 0.158 | < .001 | -0.868 | -0.249 | -.172 |  | -0.461 | 0.083 | < .001 | -0.623 | -0.299 | -.134 |
| Liking on volunteering | 0.386 | 0.080 | < .001 | 0.229 | 0.542 | .371 |  | 0.499 | 0.045 | < .001 | 0.411 | 0.587 | .438 |
| Kama Muta on volunteering | 0.157 | 0.110 | .154 | -0.059 | 0.373 | .139 |  | 0.052 | 0.058 | .370 | -0.062 | 0.165 | .043 |
| Admiration on volunteering | -0.131 | 0.119 | .269 | -0.364 | 0.101 | -.118 |  | 0.152 | 0.062 | .014 | 0.031 | 0.273 | .127 |
| Anger on volunteering | -0.071 | 0.073 | .331 | -0.213 | 0.072 | -.048 |  | -0.267 | 0.035 | < .001 | -0.335 | -0.198 | -.187 |
| Indirect effects via |  |  |  |  |  |  |  |  |  |  |  |  |  |
| Liking | 0.345 | 0.091 | < .001 | 0.167 | 0.523 | .106 |  | 0.354 | 0.053 | < .001 | 0.250 | 0.457 | .103 |
| Kama muta | 0.068 | 0.053 | .195 | -0.035 | 0.172 | .021 |  | 0.023 | 0.026 | .376 | -0.028 | 0.073 | .007 |
| Admiration | -0.068 | 0.064 | .290 | -0.193 | 0.058 | -.021 |  | 0.077 | 0.033 | .022 | 0.011 | 0.142 | .022 |
| Anger | 0.036 | 0.037 | .341 | -0.038 | 0.109 | .011 |  | 0.080 | 0.021 | < .001 | 0.038 | 0.121 | .023 |
| *Model fit* | | | | | | | | | | | | | |
| *CFI* | 1.000 | | | | | | | | | | | | |
| *TLI* | 1.000 | | | | | | | | | | | | |
| *RMSEA* (90% *CI*) | 0.000 (0.000, 0.000) | | | | | | | | | | | | |
| *SRMR* | 0.000 | | | | | | | | | | | | |

*Note.* Condition = Neutral vs. Smiling Display; Donation = Donation intentions; Volunteering = Volunteering intentions; *B* = Non−standardized estimates; *SE* = Standard error; β = Standardized coefficients of the model; *CFI* = Comparative Fit Index. *TLI* = Tucker−Lewis Index; *RMSEA* = Root Mean Square Error of Approximation; *SRMR* = Standardized Root Mean Square Residual.

We found that smiling had indirect effects on donation intentions via liking (both ingroup and outgroup), increased admiration (only outgroup) and decreased anger (only ingroup). Finally, in the case of volunteering, we also found the effects through liking and additionally, in the case of outgroup members, admiration and anger.

# References

Ma, D.S., Correll, J. & Wittenbrink, B. (2015). The Chicago face database: A free stimulus set of faces and norming data. *Behav Res 47*, 1122–1135. https://doi.org/10.3758/s13428-014-0532-5

R Core Team. (2014). *R: A language and environment for statistical computing*. Vienna, Austria: R Foundation for Statistical Computing.

Rosseel, Y. (2014). lavaan: an R package for structural equation modeling. *Journal of Statistical Software, 48*(2), 1–21. <https://doi.org/10.18637/jss.v048.i02>

Stanley, D. (2018). *Package “apaTables”: Create American Psychological Association (APA) Style Tables*. R package version 2.0.5. Retrieved from <https://github.com/dstanley4/apaTables>

1. The information in the tables with path analyses shows all the regression coefficients and effects sizes. The significant indirect effects are described after each table. Correlation analyses were conducted in R (R Core Team, 2014) with the package *apaTables* (Stanley, 2018) and path analyses with the package *lavaan* (Roseel, 2014). [↑](#footnote-ref-1)
